# Helical Bilayer Nonbenzenoid Nanographene Bearing a [10]Helicene with Two Embedded Heptagons

**DOI:** 10.1002/anie.202216193

**Published:** 2022-12-20

**Authors:** Lin Yang, Yang‐Yang Ju, Miguel A. Medel, Yubin Fu, Hartmut Komber, Evgenia Dmitrieva, Jin‐Jiang Zhang, Sebastian Obermann, Araceli G. Campaña, Ji Ma, Xinliang Feng

**Affiliations:** ^1^ Center for Advancing Electronics Dresden (cfaed) & Faculty of Chemistry and Food Chemistry Technische Universität Dresden 01062 Dresden Germany; ^2^ State Key Laboratory for Physical Chemistry of Solid Surfaces Department of Chemistry College of Chemistry and Chemical Engineering Xiamen University Xiamen 361005 China; ^3^ Departamento de Química Orgánica, Unidad de Excelencia de Química (UEQ). Facultad de Ciencias. Universidad de Granada 18071 Granada Spain; ^4^ Leibniz-Institut für Polymerforschung Dresden e.V. Hohe Straße 6 01069 Dresden Germany; ^5^ Leibniz Institute for Solid State and Materials Research Helmholtzstr. 20 01069 Dresden Germany; ^6^ Max Planck Institute of Microstructure Physics Weinberg 2 06120 Halle Germany

**Keywords:** Bilayer, Chirality, Helicene, Heptagon, Nanographene

## Abstract

The precision synthesis of helical bilayer nanographenes (NGs) with new topology is of substantial interest because of their exotic physicochemical properties. However, helical bilayer NGs bearing non‐hexagonal rings remain synthetically challenging. Here we present the efficient synthesis of the first helical bilayer nonbenzenoid nanographene (**HBNG1**) from a tailor‐made azulene‐embedded precursor, which contains a novel [10]helicene backbone with two embedded heptagons. Single‐crystal X‐ray analysis reveals its highly twisted bilayer geometry with a record small interlayer distance of 3.2 Å among the reported helical bilayer NGs. Notably, the close interlayer distance between the two layers offers intramolecular through‐space conjugation as revealed by in situ spectroelectrochemistry studies together with DFT simulations. Furthermore, the chiroptical properties of the P/M enantiomers of **HBNG1** are also evaluated by circular dichroism and circularly polarized luminescence.

## Graphical abstract

A helical bilayer nonbenzenoid nanographene has been synthesized with a novel [10]helicene backbone bearing two heptagons. Single‐crystal X‐ray analysis revealed its highly twisted bilayer geometry with a small interlayer distance of 3.2 Å, in which the intramolecular through‐space conjugation between the two layers is determined. Furthermore, the chiroptical properties of the *P*/*M* enantiomers of this helical nanographene have also been evaluated.

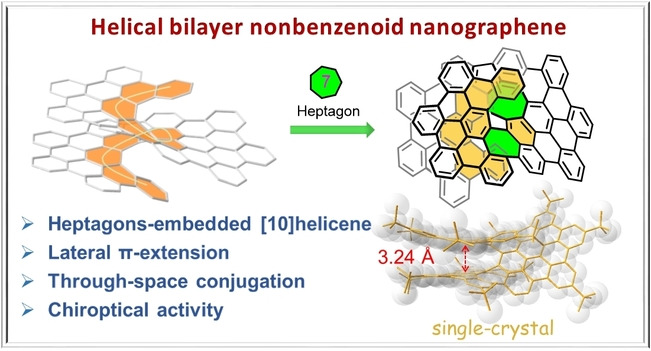

The study of helical nanographenes (NGs) has attracted increasing attention in recent years due to their aesthetically chiral structures, dynamic behavior as well as intriguing physicochemical properties.^[1]^ Recent studies have demonstrated that embedding non‐hexagonal rings (i.e., pentagon, heptagon or octagon) into the helicene backbone of helical NGs is a feasible strategy to tune their molecular geometries, enantiomerization barrier, and chiroptical properties.^[2]^ For example, the replacement of the six‐membered ring in the [5]‐ or [7]helicene backbone of NGs by a heptagonal (**I** and **II**, Figure 1a),^[3]^ octagonal^[4]^ or nonagonal^[5]^ ring significantly increases the configurational stability and enhances chiroptical properties, which renders their promising applications in spin filters or chiroptical sensors.[^1a^, ^6^] However, the preparation of heptagon‐embedded [n]helicene with *n*>7 is still quite challenging due to the incremental stabilization energy in higher [n]helicenes, thus limiting the study of structure–property relationship in this helicene family.^[7]^ In addition to the embedded non‐hexagonal rings, the incorporation of a folding layer into helical NGs offers unique intramolecular π–π overlap and electronic communication within the molecule, creating the possibility of using it as twistronic or spintronic material.^[8]^ For instance, Martín's group reported the helical bilayer nanographene (NG) with pristine [10]helicene backbone bearing two hexa‐*peri*‐hexabenzocoronene as end‐units (**III**, Figure 1b). The rigidity of the helicene linker forces the two NG layers to adopt a bilayer geometry,^[9]^ which not only maintains the π‐conjugation through the molecule but also bestows the resultant π‐system with unique chirality. Larger NG helicoids bearing central π‐extended helicenes or an increasing number of NG blades leads to enhanced π‐delocalization in novel aromatics.[^8b^, ^10^] Despite the very few examples of helicene‐bridged bilayer NGs that have been reported,[^9^, ^11^] the helical bilayer NG containing nonbenzenoid substructure is essentially unknown so far mostly due to the lack of a proper synthetic strategy to incorporate non‐hexagonal rings into the higher helicene backbone.

**Figure 1:**
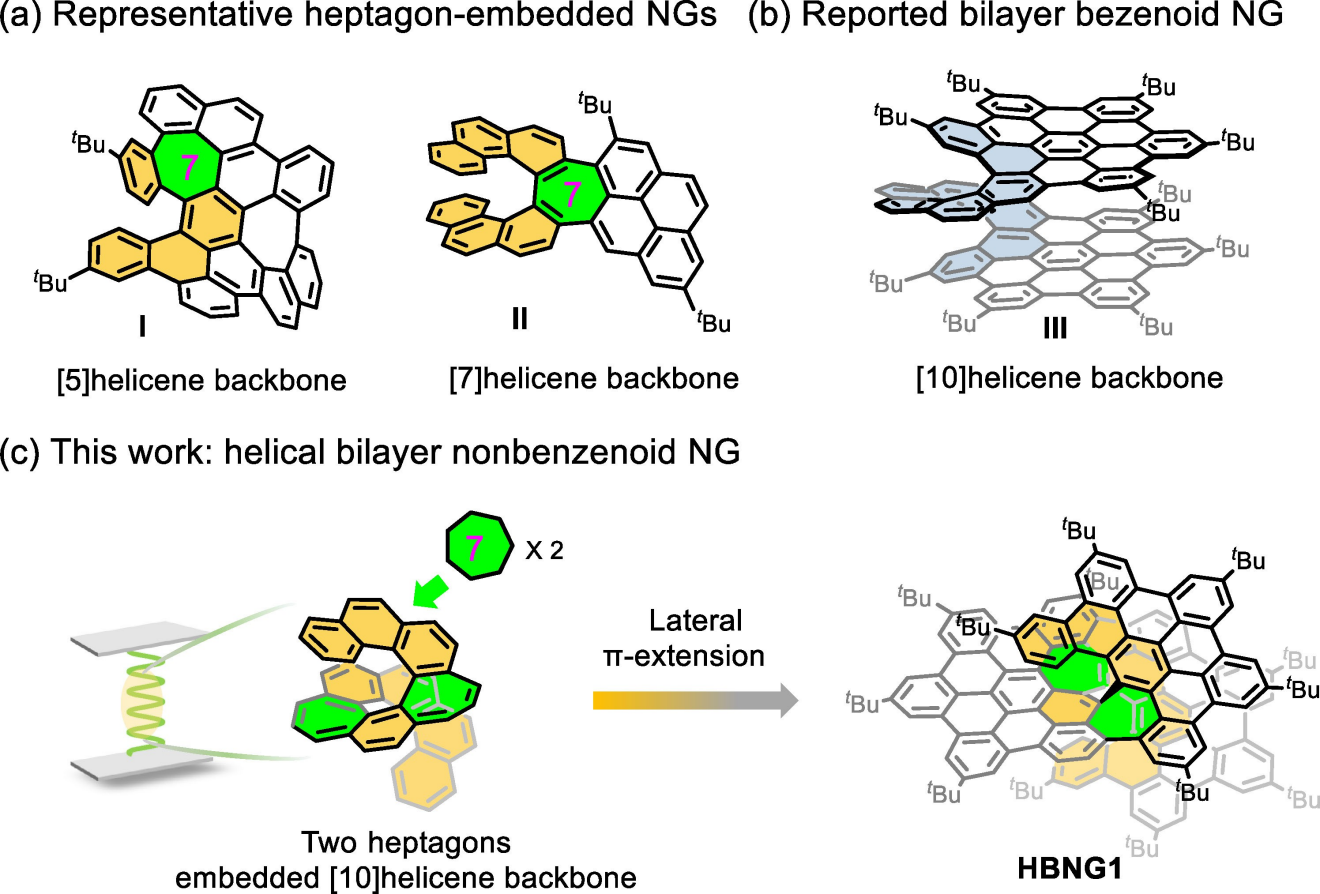
(a) Representative heptagon‐embedded NGs. (b) Example of reported helical bilayer benzenoid NG. (c) **HBNG1** reported in this work.

Herein, we report the synthesis of the first example of a helical bilayer nonbenzenoid nanographene (**HBNG1**) containing a [10]helicene with two embedded heptagons as a novel chiral moiety (Figure 1c). The second layer in **HBNG1** is well constructed based on the predesigned precursor **5** with defined conformation by an efficient Scholl reaction. **HBNG1** is composed of 35 fused rings with one pentagon and two heptagons. The introduction of the second heptagon largely overcomes the steric hindrance within the inner helical rim to form this highly congested bilayer structure. Single‐crystal X‐ray diffraction (SCXRD) analysis of **HBNG1** clearly elucidates its highly twisted bilayer structure with a close interlayer distance of 3.2 Å. The extensive intramolecular interaction between the two layers is demonstrated by 2D NMR spectroscopy and density functional theory (DFT) simulation. The embedded heptagons exhibit large nonplanarity values and strong antiaromatic characters. Cyclic voltammetry (CV) analysis indicates its amphoteric redox properties and its oxidized states are fully investigated by in situ spectroelectrochemistry (SEC). More interestingly, the small interlayer distance in **HBNG1** induces the unique intramolecular through‐space interaction between the two layers, which is clearly revealed by the SEC measurements together with the calculations. Furthermore, the *P*/*M* enantiomers of **HBNG1** are resolved by chiral high‐performance liquid chromatography (HPLC), and their chiroptical properties are studied by electronic circular dichroism (ECD) and circularly polarized luminescence (CPL). This study will stimulate the design and synthesis of other novel helical bilayer or multilayer NGs incorporated with nonbenzenoid substructures.

The synthesis of **HBNG1** starts from heptagon‐embedded super[6]helicene **2** that has been described in our previous report (Scheme 1).^[12]^ First, the iodine‐substituted compound **2** was coupled with 4‐*tert*‐butylphenylacetylene through a Sonogashira reaction to give the intermediate **3** in 82 % yield. Subsequent Diels–Alder reaction between **3** and 2,3,4,5‐tetrakis(4‐*tert*‐butyl‐phenyl)cyclopentadienone (**4**) afforded the key precursor **5** in 52 % yield. After that, the Scholl reaction of **5** using DDQ/TfOH at 0 °C for 30 minutes provided the **HBNG1**, together with partially cyclized and oxidized byproducts, resulting in difficult purification (Figure S1). Based on this result, we reduced the reaction time to 15 minutes, yielding a mixture of **HBNG1** and partially cyclized compounds without the presence of unwanted oxidized byproducts. After a simple work‐up, the obtained crude residue was further treated under the same reaction conditions. To our delight, the partially cyclized byproducts were fully converted and the target **HBNG1** was isolated as a red solid with a yield of 31 % over two steps.

**Scheme 1:**
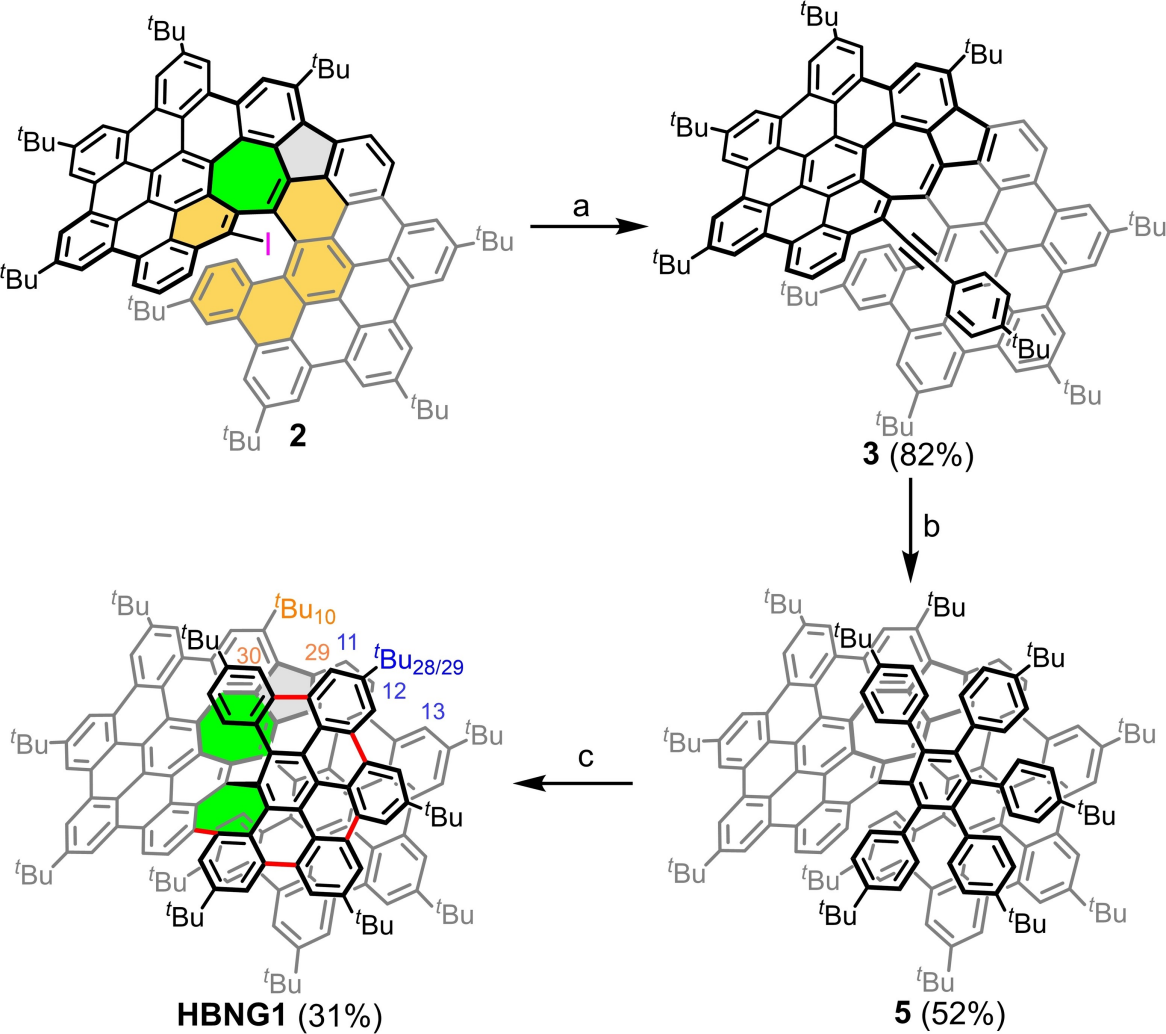
Synthetic route to the **HBNG1**. Reagents and conditions: (a) CuI, PdCl_2_(PPh_3_)_2_, NEt_3_, 90 °C, 12 h; (b) 2,3,4,5‐tetrakis(*p*‐*tert*‐butylphenyl)cyclopentadienone (**4**), Ph_2_O, 265 °C, 2 days; (c) DDQ, TfOH, DCM, 0 °C, 15 min, repeat two times. DDQ: 2,3‐dichloro‐5,6‐dicyano‐1,4‐benzoquinone, TfOH: trifluoromethanesulfonic acid, DCM: dichloromethane.

The formation of **HBNG1** from precursor **5** was first confirmed by matrix‐assisted laser desorption/ionization‐time‐of‐flight mass spectrometry (MALDI‐TOF MS). The experimental mass spectrum shows the desired ion [M]^+^ with an error of +1.32 ppm to the theoretical value and matches the simulated isotopic distribution pattern perfectly (Figure 2a). Notably, **HBNG1** exhibits good solubility in common organic solvents, such as n‐hexane, DCM, THF and toluene, owing to its highly twisted geometry. The structure of **HBNG1** was then fully characterized by means of ^1^H, ^13^C and 2D NMR experiments (Figure S3–11). The proton sequence was determined by evaluating long‐range correlations (lr‐COSY) and through‐space correlations observed by rotating‐frame nuclear Overhauser effect spectroscopy (ROESY). Interestingly, ROESY correlations between both layers were observed (Figure 2b and Figure S7). For example, the phenyl‐hydrogens H_11_ (8.31 ppm), H_12_ (8.22 ppm) and H_13_ (8.61 ppm) located on the plane of centroid rings C and D (Figure 3c) show ROESY correlations to the hydrogens of the *tert*‐butyl group ^*t*^Bu_28/29_ (1.95 ppm) on the plane of centroid ring I, indicating the close distance between the two layers (Scheme 1 and Figure 2b).

**Figure 2:**
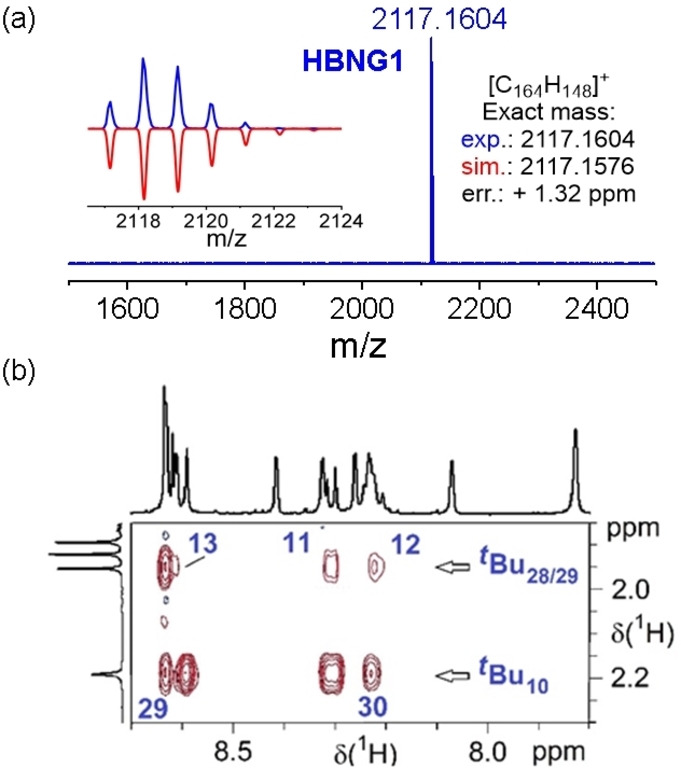
(a) HR MALDI‐TOF mass spectra of **HBNG1**. (b) The region from the ROESY spectrum of **HBNG1** with assigned correlations between protons of different layers due to spatial proximity. For the details see the Supporting Information.

**Figure 3:**
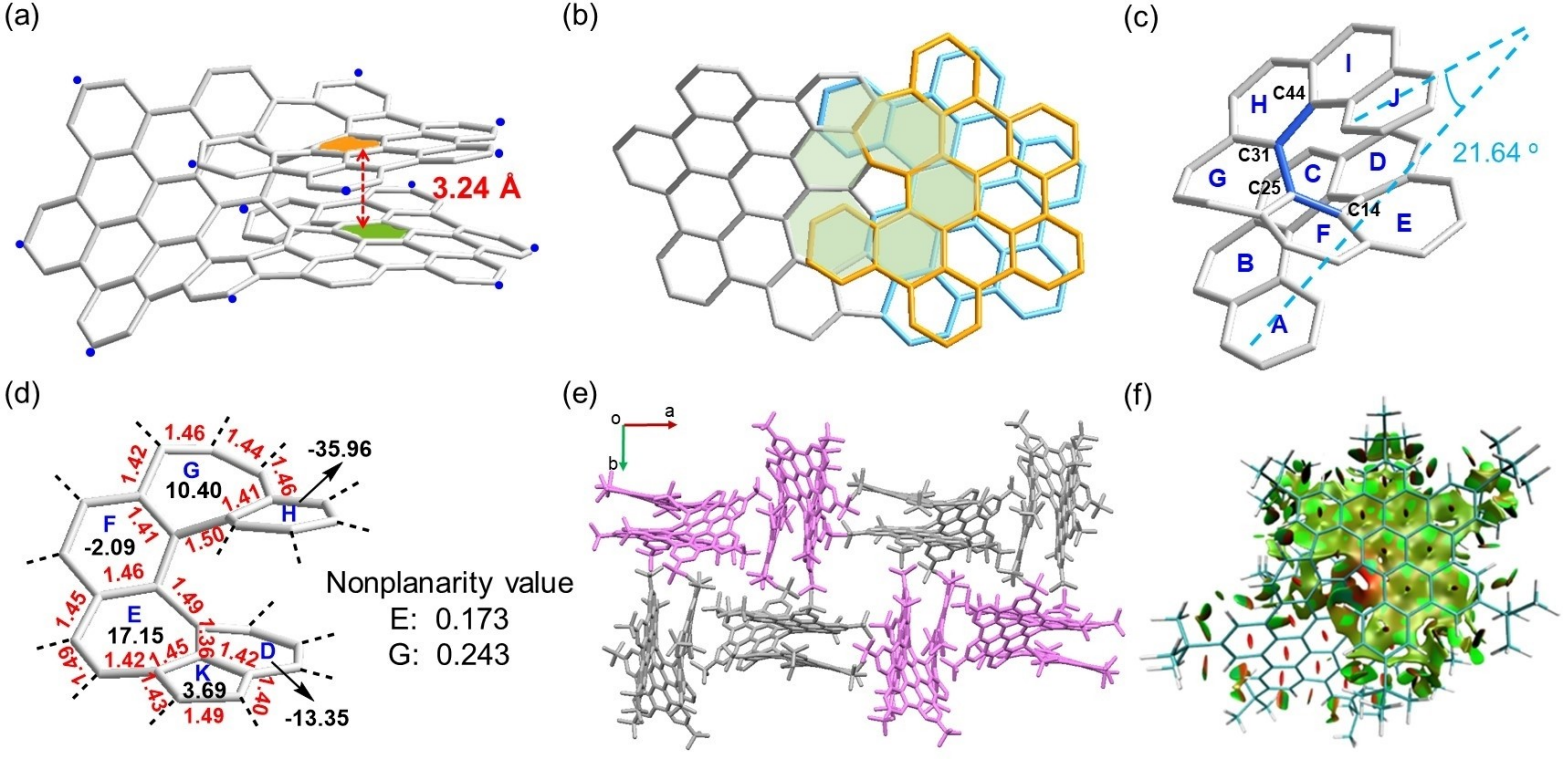
Single‐crystal structure of **HBNG1** (a) side view, (b) top view. Hydrogen atoms and ^*t*^Bu groups are omitted for clarity (blue dot, ^*t*^Bu). (c) Single‐crystal structure of the embedded [10] helicene with two heptagons. (d) The nonplanarity value of the heptagons, the bond lengths (in Å) of the embedded pentagon and heptagons as well as their NICS(1)_zz_ values, calculated at the GIAO‐B3LYP/6‐31^+^G(2d,p) level. (e) Molecular packing of **HBNG1**. *M*‐ and *P*‐enantiomers are colored in light grey and pink, respectively. (f) Plots of the RDG isosurfaces for **HBNG1**

A suitable crystal was grown by slow vapor diffusion of methanol into a solution of **HBNG1** in chlorobenzene.^[13]^ SCXRD analysis revealed its unique helical bilayer geometry bearing two heptagons in the [10]helicene of the inner rim (Figure 3a and Figure S12). Three distinct NG blades were laterally fused to a [10]helicene, forming a twisted bilayer nonbenzenoid structure (Figure 3b). The average perpendicular distance between the centroid rings C and H in **HBNG1** equals 3.24 Å (Figure 3a and Figure S13),^[14]^ which is a record small distance among those of the reported helical bilayer NGs, as a result of the embedded heptagons. The angle between the terminal rings A and J in the inner [10]helicene (*θ*=21.64°) indicates a more parallel arrangement compared with the reported **III** (*θ*=42.76°) (Figure 3c),^[9]^ suggesting the highly distorted geometry of **HBNG1**. In addition, the angle between the two planes of the centroid rings C and H is determined as 9.9° (Figure S12), much larger than the value in molecule **III** (2.04°).^[9]^ Remarkably, an average torsion angle of 29.6° is found in the inner rim of **HBNG1**, and the highest torsion angle is 49.5° near the heptagonal ring G (C14−C25−C31−C44, marked in blue in Figure 3c), which is one of the largest angles among the reported heptagon‐embedded helical nanographene.[^3^, ^12^, ^15^] The deformation of C−C−C bond angles away from the natural value of 120° also illustrates the highly twisted geometry, ranging from 106.2° to 132.4° in the azulene and heptagon units (Figure S14). The nonplanarity value^[16]^ of the embedded heptagonal ring G equals 0.243 Å (Figure 3d), revealing a highly distorted ring conformation. Furthermore, the C−C bond lengths in the heptagonal ring G lie within the range of 1.41–1.50 Å, which are significantly longer than that of typical C(sp^2^)−C(sp^2^) bond in benzene (1.39 Å). In addition, **HBNG1** crystallizes in the I 4_1_/a space group with a pair of *M*‐ and *P*‐helicenes. In the packing model, the two adjacent molecules are almost located at a perpendicular angle to each other due to the C−H−π interactions (the surrounding *tert*‐butyl groups block the interactions between π‐faces, Figure 3e).

To determine the attractive interlayer interaction in **HBNG1**, reduced density gradient (RDG) simulations were performed. As shown in Figure 3f, there are strong non‐covalent van der Waals interactions (green surface) between the two layers (see details in Figure S22). In addition, the strong steric hindrance in the helical inner rim (red color in Figure 3f) is also revealed by RDG analysis. The nucleus‐independent chemical shift (NICS) and anisotropy of the induced current density (ACID) analyses are used to get further insight into the aromaticity of **HBNG1**. NICS(1)_zz_ calculations indicate that the pentagon exhibits a slight antiaromatic feature (ring K: 3.69 ppm) and the heptagons show strong anti‐aromatic character (ring E: 17.15 ppm; ring G: 10.40 ppm) (Figure 3d and Figure S23). Furthermore, the ACID simulations also exhibit counter‐clockwise ring current for the embedded heptagon and azulene subunit, demonstrating their antiaromatic behavior (Figure S24).

The UV/Vis spectrum of **HBNG1** shows a broad absorption together with a hump peak from 503 to 561 nm (Figure 4a). According to the time‐dependent density functional theory (TD‐DFT) calculations, the broad hump absorption bands are attributed to the combination of HOMO→LUMO+1, HOMO−1→LUMO and HOMO−2→LUMO transitions (Figure S25 and Table S3). The longest wavelength absorption band of **HBNG1** is centered at 628 nm and absorbs up to 680 nm due to the extended π‐conjugation. The optical energy gap of **HBNG1** is determined to be 1.86 eV from the onset of its UV/Vis absorption. The emission spectrum of **HBNG1** in DCM solution displays a maximum at 602 nm with a shoulder at 665 nm (Figure S16) and its photoluminescence quantum yield in DCM is estimated to be 0.32 %.[^15a^, ^17^] In addition, the electrochemical behavior of **HBNG1** was investigated by CV and square wave voltammetry (SWV). **HBNG1** exhibits four reversible oxidation with half‐wave potentials at 0.20, 0.49, 0.97 and 1.25 V, and one reversible reduction wave at −1.93 V (vs. Fc^+^/Fc) was identified in the CV curve. SWV measurements provided an apparent observation of the highest oxidation event (Figure 4b). The CV data at different scan rates reveal the good electrochemical reversibility of the first two‐oxidative waves of **HBNG1** (Figure S20). The HOMO/LUMO levels are thus estimated to be −4.9/−3.01 eV based on the onset potentials of the first oxidation/reduction waves. The electrochemical energy gap is thus calculated to be 1.89 eV, which is in good accordance with its optical energy gap.

**Figure 4:**
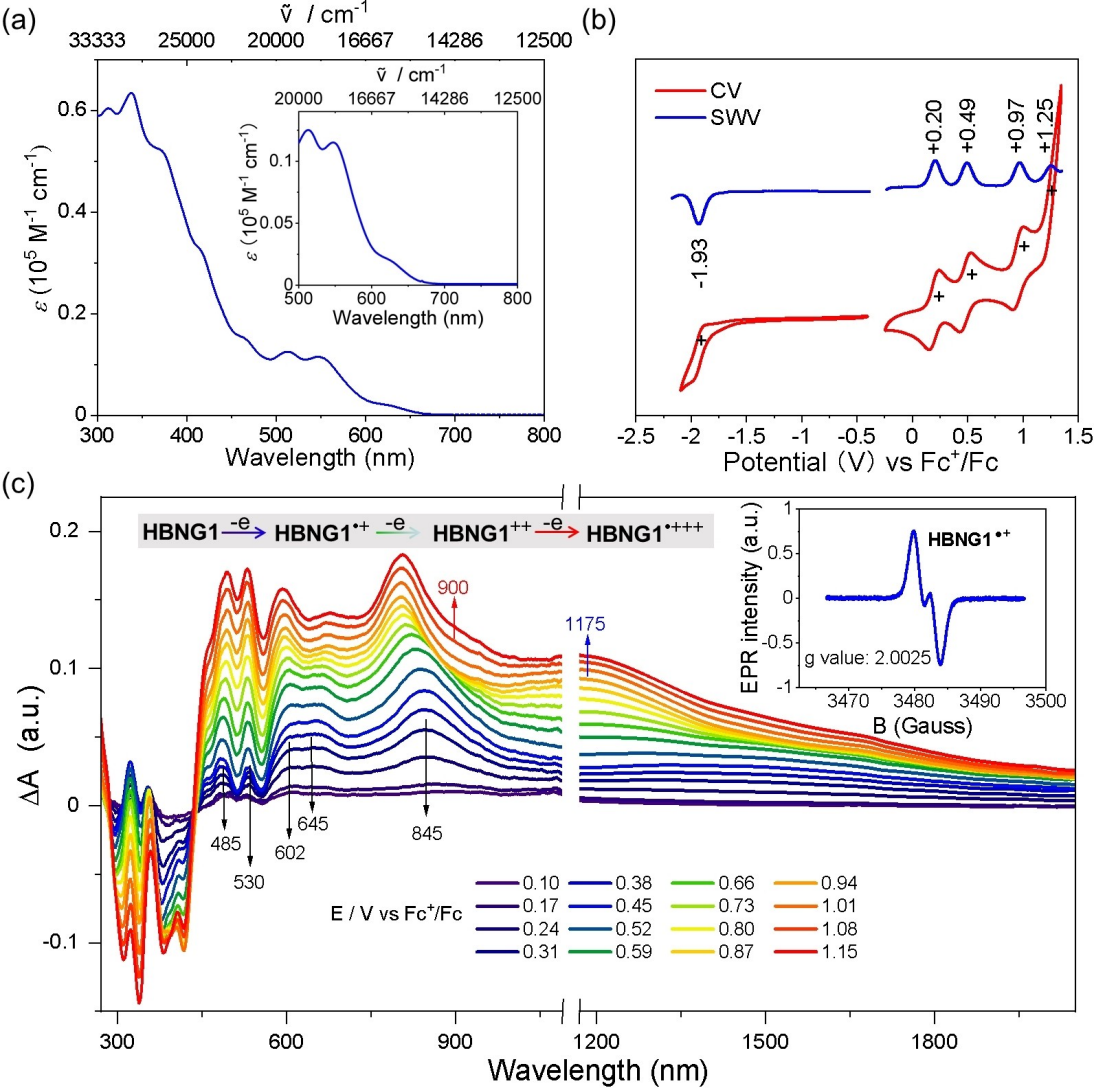
(a) UV/Vis absorption spectrum of **HBNG1** in DCM (1×10^−5^ M). (b) CV and SWV scans of **HBNG1** in DCM containing 0.1 m *n*Bu_4_NPF_6_ against Fc^+^/Fc at a scan rate of 50 mV s^−1^ at room temperature. (c) In situ UV/Vis‐NIR spectra measured in DCM during the oxidation of **HBNG1**. Inset: EPR spectrum of the radical cation of **HBNG1**.

Considering the lower oxidation potentials of **HBNG1**, its redox behavior was further studied by in situ electron paramagnetic resonance (EPR)/UV/Vis‐NIR SEC. During the first oxidation, the absorption bands at 485, 530, 602, 645 and 845 nm dominate in the UV/Vis‐NIR spectra (Figure 4c). Simultaneously, an EPR signal with a *g* value of 2.0025 was obtained, indicating the formation of radical cation species (Figure 4c). The second oxidation leads to the appearance of the absorption band at 1175 nm, while the bands at 485 and 845 nm are red and blue shifted, respectively. The EPR intensity decreases during the second oxidation indicating that the dication specie has a diamagnetic dication character (Figure S21). The formation of the radical trication during further oxidation is indicated by the increase in the EPR intensity and appearance of the band around 900 nm (Figure 4c). Interestingly, the observation of an intervalence charge transfer band in the NIR region for the one‐oxidized species (Figure 4c) as well as the corresponding TD‐DFT calculations reveal the through‐space conjugation among the two layers in **HBNG1** (Table S2). The calculated electronic coupling (*V*_12_) value is larger than half of the reorganization energy (λ/2), indicating that it is a Robin‐Day class III mixed‐valence compound.^[18]^ In addition, the spin density of **HNBG1**⋅^**+**^ reflects the electronic communication between these two layers based on the calculation (Figure 5).

**Figure 5:**
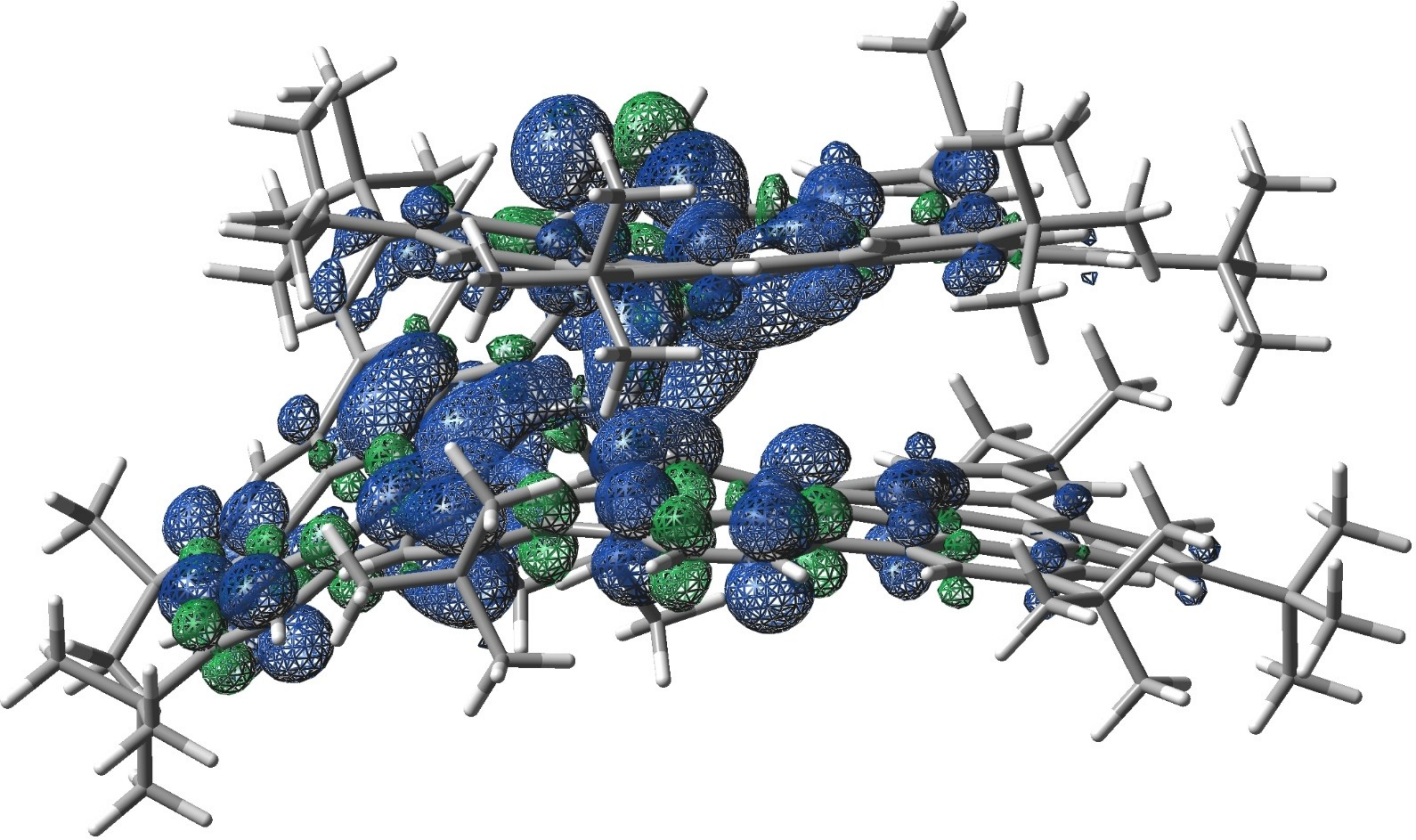
Calculated spin density of **HBNG1**⋅^+^ (isosurface value of 0.0005 au).

The resolution of the racemic **HBNG1** into its two enantiomers was achieved by chiral stationary phase HPLC (CSP‐HPLC). A full CSP‐HPLC optimization study allowed the identification of the best chromatographic conditions for its enantioresolution (Figure S15 and Table S1). A cellulose‐based stationary phase and a gradient of n‐hexane/DCM mixture as mobile phase at room temperature were used for this purpose. After enantioresolution, their chiroptical properties were studied (Figure 6). The ECD response was measured for the two collected fractions. Both enantiomers displayed mirror images with several opposite Cotton effects in the UV/Vis region. The first eluted fraction showed two bands of major intensity with negative Cotton effect; the first one at 360 nm (|Δϵ|=95.9 M^−1^ cm^−1^, *g*_abs_=|Δϵ|/ϵ=1.9×10^−3^), the second one at 553 nm (|Δϵ|=70.1 M^−1^ cm^−1^, *g*_abs_=6.6×10^−3^), which is comparable with the reported heptagon‐embedded helicenes.[^3b^, ^15d^] Besides, two minor bands with opposite Cotton effect at 318 nm (|Δϵ|=41.3 M^−1^ cm^−1^, *g*_abs_=7.3×10^−4^) and 435 nm (|Δϵ|=22.6 M^−1^ cm^−1^, *g*_abs_=1.0×10^−3^) were observed (Figure 6).

**Figure 6:**
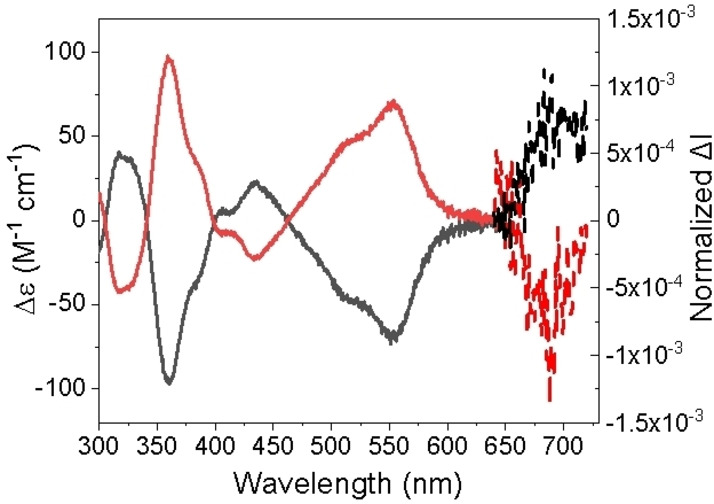
Experimental ECD spectra (solid line) and experimental CPL spectra (dashed line, irradiation at 525 nm) of *P*‐ (red) and *M‐* (black) **HBNG1** (DCM at ca. 1.6×10^−5^ and 1.2×10^−5^ M of *P*‐ and *M‐* **HBNG1**, respectively).

Aiming to assign the absolute configuration of the two collected fractions, a comparison between the experimental ECD spectra and TD‐DFT simulated spectrum for **HBNG1** was done (Figure S26). In this sense, the first fraction was assigned to the *M*‐enantiomer and the second one to the *P*‐enantiomer. Furthermore, the CPL spectra showed a maximum centered at 688 nm with a similar profile to the corresponding fluorescence spectrum. The enantiomeric forms gave CPL spectra of opposite signs, which are in agreement with the corresponding one of the last bands in the ECD spectrum. Thus, from the TD‐DFT calculation of *M*‐**HBNG1**, the first transition shows a low oscillatory strength and a positive rotatory strength (Figure S25), in agreement with the positive CPL spectrum obtained (Figure 6, dashed line in black). This first ECD band of the opposite sign to the main band located at 553 nm is not experimentally observed in the CPL spectrum due to its low oscillatory strength. The luminescence dissymmetry factor (*g*_lum_) reaches up to 1.3 ×10^−3^ (Figure S18b). The observed *g*_lum_ values are in the range of other heptagon‐embedded helical nanographenes that have been reported.[^3b^, ^8b^] Remarkably, the highest value of the dissymmetric factor of **HBNG1** is higher than the aza[10]helicenes,^[19]^ which attributes to the rigid geometry and separated molecular orbitals via the azulene unit.^[20]^ We studied the racemization of **HBNG1** by means of variable temperature ECD kinetic measurements. Notably, neither racemization nor decomposition was observed after 4 h of heating a solution of *P*‐**HBNG1** in toluene at 373 K, indicating the high configurational stability of **HBNG1**. Unfortunately, heating at higher temperatures (473 K, for 2 h) led to decomposition, which prevents the calculation of the racemization barrier.

In summary, we have demonstrated a strategy to synthesize a novel chiral bilayer nonbenzenoid NG containing a [10]helicene backbone with two embedded heptagons. The embedded heptagons in the helical backbone result in the highly twisted and rigid bilayer geometry of **HBNG1**, as clearly demonstrated by X‐ray diffraction analysis and DFT calculations. Remarkably, **HBNG1** exhibits a close interlayer distance of 3.2 Å and intramolecular through‐space interaction between the two layers. **HBNG1** shows amphoteric redox properties, and in situ SEC was used to evaluate its stable oxidized states. Moreover, enantioresolution of **HBNG1** was achieved by chiral HPLC, with the enantiomers showing good ECD and CPL responses, reaching values of *g*_abs_ up to 6.6×10^−3^ and *g*_lum_ up to 1.3×10^−3^. Therefore, this work facilitates the design of the π‐extended helical nonbenzenoid NGs with multilayer structures. Currently, related studies are ongoing in our laboratories.

## Conflict of interest

The authors declare no conflict of interest.

## Supporting information

As a service to our authors and readers, this journal provides supporting information supplied by the authors. Such materials are peer reviewed and may be re‐organized for online delivery, but are not copy‐edited or typeset. Technical support issues arising from supporting information (other than missing files) should be addressed to the authors.

Supporting Information

Supporting Information

## Data Availability

The data that support the findings of this study are available from the corresponding author upon reasonable request.
