# Supplementary material for: Helical Bilayer Nonbenzenoid Nanographene Bearing a [10]Helicene with Two Embedded Heptagons
Source: Angew Chem Int Ed Engl. 2022 Dec 20;62(4):e202216193. doi: 10.1002/anie.202216193 (PMC10107200; doi:10.1002/anie.202216193)
Supplement: Supplementary file 1 — Supporting Information [file ANIE-62-0-s001.pdf]

Angewandte Chemie
Eine Zeitschrift der Gesellschaft Deutscher Chemiker

Supporting Information

# Helical Bilayer Nonbenzenoid Nanographene Bearing a [10]Helicene with Two Embedded Heptagons

*L. Yang, Y.-Y. Ju, M. A. Medel, Y. Fu, H. Komber, E. Dmitrieva, J.-J. Zhang, S. Obermann, A. G. Campaña, J. Ma*, X. Feng**

# Table of contents


1. General methods and materials S3

2. Detailed synthetic procedure and characterization data of **HBNG1** S5

3. High resolution (HR) MALDI-TOF mass spectrum of **HBNG1** S10

4. NMR spectra of **HBNG1** S12

5. X-ray crystallographic analysis of **HBNG1** S19

6. Determination of enantiopurity **HBNG1** by HPLC S21

7. Photophysical study S23

8. Configurational stability study of **HBNG1** by VT-ECD and HPLC S25

9. CV and *in situ* spectroelectrochemistry of **HBNG1** S26

10. DFT calculations S27

11. Structure and property comparison of **2** and **HBNG1** S31

12. NMR spectra S33

13. High-resolution mass spectrometry (HR-MS) S44

14. References S45

## 1. General methods and materials

All the reagents were obtained from Sigma Aldrich, TCI, abcr, Alfa Aesar, Strem, fluorochem, and chempur. All these chemicals were used as received without further purification. All reactions dealing with air- or moisture-sensitive compounds were carried out in a dry reaction vessel under argon (Ar) atmosphere by using standard vacuum-line and Schlenk techniques. Anhydrous dichloromethane and tetrahydrofuran were obtained from MBRAUN MB-SPS-5 solvent purification system.

Thin layer chromatography (TLC) was performed on silica-coated aluminium sheets with a fluorescence indicator (TLC silica gel 60 F254, purchased from Merck KGaA).

Column chromatography was performed on silica ($SiO_2$, particle size 0.063-0.200 mm, purchased from VWR).

NMR spectra were recorded on a Bruker Avance III 500 spectrometer operating at 500.13 MHz for $^{1}H$ and at 125.77 MHz for $^{13}C$ at 30°C (unless otherwise stated). The 1D and 2D NMR spectra were recorded using the standard Bruker pulse programs. $CD_2Cl_2$ ($\delta(^{1}H)$ = 5.33 ppm, $\delta(^{13}C)$ = 53.7 ppm) and $C_2D_2Cl_4$ ($\delta(^{1}H)$ = 5.98 ppm) were used as solvents and as internal chemical shift reference. Chemical shifts ($\delta$) are reported in ppm. The following abbreviations are used to describe peak patterns as appropriate: s = singlet, d = doublet, t = triplet, q = quartet, and m = multiplet.

The high-resolution matrix-assisted laser desorption/ionization time-of-flight (MALDI-TOF) mass spectrometry was performed on a Bruker Autoflex Speed MALDI TOF MS (Bruker Daltonics, Bremen, Germany) using *trans*-2-[3-(4-*tert*-butylphenyl)-2-methyl-2-propenylidene]malononitrile (DCTB) or dithranol as matrix.

HPLC separation were carried out on an Agilent 1260 series equipped with the following modules: quaternary pump (G7111B 1260 Quat Pump), automatic sample injector (G2258A 1260 DL ALS), column thermostat (G1316A 1260 TCC), DAD detector (G7115A 1260 DADWR) and an automatic sample collector (G1364C 1260 FC-AS).

Electronic circular dichroism (ECD) and circularly polarized luminescence (CPL) were recorded in an Olis DSM172 spectrophotometer equipped with a xenon lamp of 150 W. Variable temperature ECD kinetic measurements were recorded in a Jasco J-816 equipped with a peltier module system.

UV–visible spectra were measured on an Agilent Cary 5000 UV–vis–NIR spectrophotometer by using 10 mm optical-path quartz cell at room temperature. Photoluminescence spectra were measured on PerkinElmer fluorescence spectrometer LS 55. Fluorescence quantum yield values measured using Rhodamine 6G as reference.

Cyclic voltammetry (CV) was carried out on a PARSTAT4000 potentiostat (Princeton Applied Research, Ametek, Germany) in a three-electrode cell in degassed anhydrous dichloromethane solution containing 0.1 M of tetra-*n*-butylammonium hexafluorophosphate ($n\mathrm{Bu_4NPF_6}$) at a scan rate of 50 $\mathrm{mVs^{-1}}$ at room temperature. A platinum wire, silver chloride covered silver wire, and platinum sheet were used as the working electrode, the pseudo reference electrode, and counter electrode, respectively. Ferrocene/ferrocenium ($\mathrm{Fc/Fc^+}$) redox couple was used as an internal standard.

The spectroelectrochemical experiments were performed in the optical EPR cavity (ER 4104OR, Bruker Germany). EPR spectra were recorded by the EMX X-band CW spectrometer (Bruker, Germany). UV-Vis-NIR spectra were measured using the Avantes spectrometer AvaSpec-2048x14-USB2 with the CCD detector and AvaSpec-NIR256-2.2 with the InGaAs detector applying the AvaSoft 7.5 software (Avantes, The Netherlands). A light source AvantesS3 Avalight-DH-S-BAL was used. Both, the EPR spectrometer and the UV-Vis-NIR spectrometer are linked to a HEKA potentiostat PG 390 which triggers both spectrometers. Triggering was performed by the software package PotMaster v2x90 (HEKA Electronik, Germany). Each UV-Vis-NIR spectrum was collected relative to that of the neutral (non-charged) compound at the initial potential. For standard *in situ* EPR/UV-Vis-NIR spectroelectrochemical experiments an EPR flat cell was used. A laminated gold mesh as working electrode, an AgCl-coated silver wire as pseudo reference electrode, and a platinum wire as counter electrode were used in spectroelectrochemical experiments.

## 2. Detailed synthetic procedure and characterization data of HBNG1

The precursor **2** was prepared according to literature method.[1]

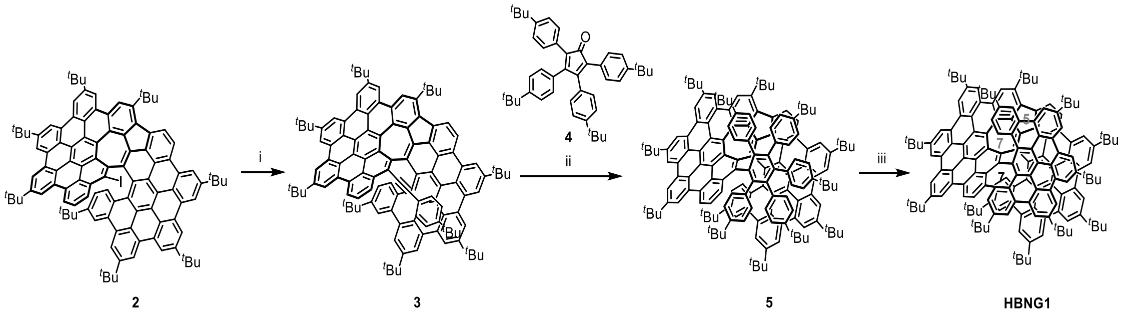

**Scheme S1**. Synthetic route of the **HBNG1**. Reagents and conditions: (i) CuI, $PdCl_2(PPh_3)_2$, $NEt_3$, 90°C, 12 h, 82%; (ii) $Ph_2O$, 265 °C, 2 d, 52%; (iii) DDQ, TfOH, DCM, 0 °C, 15 min, 2 times. DDQ: 2,3-dichloro-5,6-dicyano-1,4-benzoquinone, TfOH: trifluoromethanesulfonic acid, DCM: dichloromethane.

### Synthesis of the intermediate 3

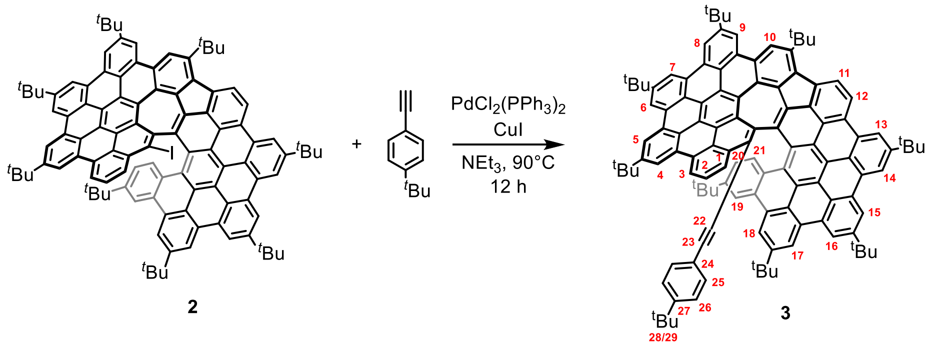

A mixture of compound **2** (152.0 mg, 0.1 mmol), $PdCl_2(PPh_3)_2$ (7.0 mg, 0.01 mmol) and CuI (5.7 mg, 0.03 mmol) in a 50 mL Schlenk flask was degassed with Ar for 30 min. Then, degassed $Et_3N$ (12 mL) and 4-*tert*-butylphenylacetylene (0.09 mL, 0.5 mmol) were added via syringe. The reaction mixture was stirred at 90 °C for 12 h. After being cooled to room temperature, the solvent was removed under reduced pressure, and the residue was subjected to column chromatography (silica gel, isohexane/DCM=6/1) to afford the product **3** (127.4 mg, 82%, red solid). The signal assignment for **3** is complicated by the presence of a second compound in the mixture that could not be separated using different techniques.

$^1$H NMR (500 MHz, $CD_2Cl_2$): δ 9.45 (s, 1H; 13), 9.43 (d, 8.4 Hz, 1H; 12), 9.36 (1H; 6), 9.32 (s, 1H; 5), 9.29 (s, 1H; 14), 9.28 (s, 1H; 7), 9.24 (1H; 10), 9.22 (s, 1H; 8), 9.21 (d, 8.4 Hz, 1H; 11), 9.20 (2 x s, 2H; 9, 15), 9.14 (2 x s, 2H; 16, 17), 9.10 (s, 1H; 4), 8.86 (d, 7.7 Hz, 1H; 3),

8.70 (s, 1H; 18), 8.58 (d, 8.3 Hz, 1H; 21), 8.00 (d, 2.0 Hz, 1H; 19), 7.95 (d, 7.8 Hz, 1H; 1), 7.75 (t, 7.8 Hz, 1H; 2), 6.06 (d, 8.4 Hz, 2H; 26), 5.65 (dd, 8.3 Hz, 2.0 Hz, 1H; 20), 5.49 (d, 8.4 Hz, 2H; 25), 2.22 (s, 9H; $t\mathrm{Bu}_{10}$), 1.89 (s, 9H; $t\mathrm{Bu}_{6/7}$), 1.88 (s, 9H; $t\mathrm{Bu}_{13/14}$), 1.85 (2 x s, 18H; $t\mathrm{Bu}_{4/5}$, $t\mathrm{Bu}_{8/9}$), 1.79 (s, 9H; $t\mathrm{Bu}_{15/16}$), 1.76 (s, 9H; $t\mathrm{Bu}_{17/18}$), 0.92 (s, 9H; $t\mathrm{Bu}_{29}$), 0.47 ppm (s, 9H; $t\mathrm{Bu}_{19/20}$).

*Note:* The signal assignment of aromatic proton signals is mainly based on sequences of ROESY and long-range COSY correlations. The ROESY correlations of the *tert*-butyl group signals to assigned aromatic proton signals allow their assignment.

$^{13}$C NMR (125 MHz, $CD_2Cl_2$): δ 150.4 (2C), 150.2 (27), 150.1, 149.6, 149.5, 149.3, 149.1, 147.2, 141.2, 139.8, 138.9, 138.8, 136.9, 133.8, 132.3, 131.5, 131.4, 131.3, 131.2 (2 C), 131.1, 131.0 (21, C), 130.9, 130.8 (2C), 130.7, 130.6, 130.4, 130.1, 129.9, 129.8, 129.6 (2C), 129.5 (25), 129.1, 128.1, 128.0, 127.6 (11), 126.7 (2), 126.3, 125.4, 125.2, 124.5, 124.2 (2C), 124.1 (2C), 123.9, 123.6 (1), 123.7, 123.4, 123.3, 123.2, 123.0 (20, C), 122.8, 122.5, 122.4, 121.8 (10), 121.7, 121.6, 121.5 (12), 121.4 (3), 120.2 (4, 8, C), 120.1 (13), 119.9 (14), 119.5 (7), 119.3 (16, 19), 119.2 (5, 17), 119.1 (6, 15), 118.9 (9, 24), 118.6 (18), 98.7 (23), 87.8 (22), 36.4, 36.0 (3C), 35.9 (2C), 35.8, 34.3 (28), 34.0, ~32.0 (6 x $CH_3$; $t\mathrm{Bu}_{4/5}$, $t\mathrm{Bu}_{6/7}$, $t\mathrm{Bu}_{8/9}$, $t\mathrm{Bu}_{13/14}$, $t\mathrm{Bu}_{15/16}$, $t\mathrm{Bu}_{17/18}$), 31.0 ($CH_3$; $t\mathrm{Bu}_{29}$), 30.7 ($CH_3$; $t\mathrm{Bu}_{10}$), 30.4 ppm ($CH_3$; $t\mathrm{Bu}_{19/20}$). *Note:* The assignment of CH and $CH_3$ signals is based on the DEPT and HSQC spectra. The signals of carbons 22 – 24 and 27 were assigned by their chemical shift (22, 23) and HMBC correlations (23, 24, 27). No further signals were assigned due to the complexity of the $^{13}$C and HMBC spectra. Several (x) overlapping signals are identified as xC.

HR-MS MALDI-TOF (m/z): calculated for $C_{120}H_{106}$ $[M]^+$, 1546.8289; found, 1546.8274, error = -0.97 ppm.

**Synthesis of the intermediate 5**

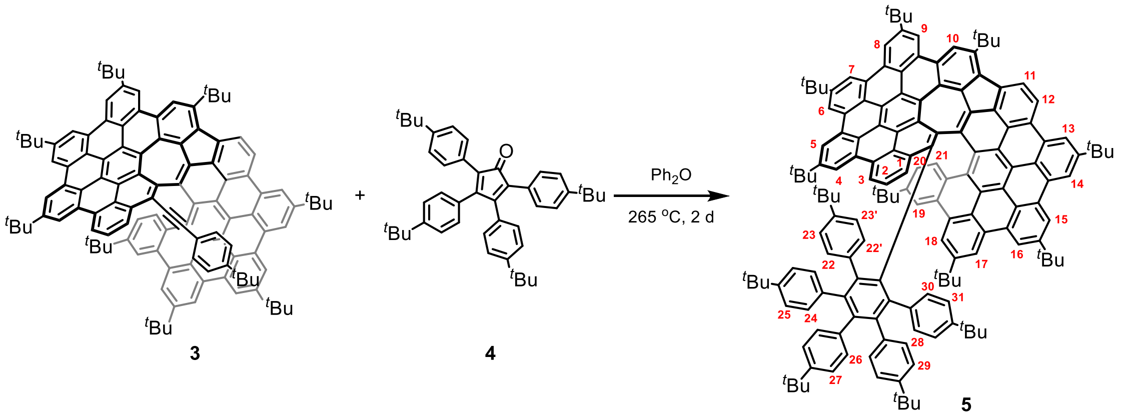

A degassed solution of **3** (30.0 mg, 0.06 mmol) and **4** (79.1 mg, 0.125 mmol) in 0.8 mL diphenyl ether was refluxed for 2 d using a heating mantle. After cooling down to room temperature, the mixture was subjected to column chromatography (silica gel, isohexane to remove the diphenyl ether, then, isohexane/DCM=8/1) to afford the product **5** (71.4 mg, 52%, red solid).

$^{1}$H NMR (500 MHz, $CD_2Cl_2$): δ 9.40 (s, 1H; 13), 9.39 (s, 1H; 15), 9.34 (s, 1H; 14), 9.33 (s, 1H; 6), 9.25 (2H; 5, 17), 9.23 (d, 8.6 Hz, 1H; 12), 9.21 (s, 1H; 16), 9.19 (s, 1H; 7), 9.11 (s, 1H; 8), 9.03 (d, 8.2 Hz, 1H; 21), 9.02 (s, 1H; 9), 9.01 (d, 8.6 Hz, 1H; 11), 9.00 (s, 1H; 10), 8.69 (s, 1H; 4), 8.61 (s, 1H; 18), 8.31 (d, 1.8 Hz, 1H; 19), 8.10 (d, 7.8 Hz, 1H; 3), 7.24 (d, 7.8 Hz, 1H; 1), 6.93 (t, 7.8 Hz, 1H; 2), 6.79 (dd, 1H; 22'), 6.66 (dd, 1H; 30'), 6.48 (dd, 1H; 30), 6.40 (dd, 1H; 28), 6.36 (dd, 1H; 23), 6.27 (dd, 1H; 26), 6.21 (2H; 23', 24), 6.14 (dd, 1H; 28'), 6.02 (dd, 1H; 26'), 6.00 (dd, 1H; 29), 5.91 (dd, 1H; 27), 5.81 (dd, 1H; 31), 5.75 (dd, 1H; 25), 5.44 (dd, 1H; 24'), 5.34 (dd, 8.2 Hz, 1.8 Hz, 1H; 20), 5.07 (dd, 1H; 25'), 4.98 (dd, 1H; 31'), 4.57 (dd, 1H; 22), 4.37 (dd, 1H; 27'), 4.34 (dd, 1H; 29'), 2.18 (s, 9H; $t\mathrm{Bu}_{10}$), 1.87 (s, 9H; $t\mathrm{Bu}_{6/7}$), 1.81 (s, 9H; $t\mathrm{Bu}_{13/14}$), 1.78 (s, 9H; $t\mathrm{Bu}_{8/9}$), 1.77 (s, 9H; $t\mathrm{Bu}_{4/5}$), 1.76 (s, 9H; $t\mathrm{Bu}_{15/16}$), 1.57 (s, 9H; $t\mathrm{Bu}_{17/18}$), 1.22 (s, 9H; $t\mathrm{Bu}_{31}$), 1.06 (s, 9H; $t\mathrm{Bu}_{25}$), 1.05 (s, 9H; $t\mathrm{Bu}_{29}$), 0.98 (s, 9H; $t\mathrm{Bu}_{27}$), 0.71 (s, 9H; $t\mathrm{Bu}_{23}$), 0.42 ppm (s, 9H; $t\mathrm{Bu}_{19/20}$). *Note:* The signal assignment is mainly based on sequences of ROESY and long-range COSY correlations. The signals of the five phenyl rings have been assigned by their COSY (e.g. 22–23 and 22'–23') and EXSY (e.g. 22–22' and 23–23') correlations. The phenyl signals showing ROESY correlations to the core are denoted as 22' (ROESY to 21), 24' (ROESY to 18 and 19), 26' (ROESY to 16 and 17), 28' (ROESY to 14 and 15) and 30' (ROESY to 12 and 13). These ROESY correlations to the core determine also the sequence of the phenyl groups within the penta(4-*tert* butylphenyl)phenyl moiety. All phenyl signals are splitted in a doublet of doublets (dd) with $^{3}J_{HH}$ ~ 8.0 Hz and $^{4}J_{HH}$ ~ 2.1 Hz. The ROESY correlations of the *tert*-butyl group signals to assigned aromatic proton signals allow their assignment.

$^{13}$C NMR (125 MHz, $CD_2Cl_2$): δ 150.1 (2C), 149.8, 149.6 (2C), 149.3, 149.0, 147.3 (2C), 146.6, 146.4, 146.1, 145.9, 142.2, 141.4 (2C), 140.6, 140.3, 139.5, 139.3 (2C), 138.0, 137.7 (2C), 137.2 (2C), 137.1, 136.7, 136.4, 135.8, 133.9 (2C), 133.2, 132.9 (25), 132.5 (31'), 131.6 (21, 27'), 131.5 (27), 131.4, 131.3 (2', C), 131.2 (22, C), 131.1, 131.0 (22', C), 130.9, 130.8, 130.7 (2C), 130.6, 130.5 (25', C), 130.4, 130.2 (29), 130.1, 130.0, 129.9 (31), 129.6, 128.8, 128.0, 127.9, 127.4 (1), 127.2 (11), 126.2, 125.9, 125.6 (2C), 125.0, 124.7, 124.2, 124.0 (2C), 123.9, 123.5, 123.3, 123.2 (23), 123.0, 122.6 (30', C), 122.4 (26'), 122.3 (24, 28'), 122.2, 122.0, 121.9 (28), 121.8 (26), 121.7 (24'), 121.6 (23'), 121.5, 121.4 (20), 121.1 (10, 12), 121.0 (3), 120.2,

120.0 (13), 119.9 (8), 119.7 (4), 119.3 (14), 119.2 (19), 119.1 (7, 15, C), 119.0 (6, 17), 118.7 (16), 118.4 (18), 118.3 (5), 118.2 (9), 36.2, 35.9 (4C), 35.8, 35.6, 34.2, 34.0, 33.9 (3C), 33.8, 32.0 (3 x $CH_3$; $t\text{Bu}_{6/7}$, $t\text{Bu}_{13/14}$, $t\text{Bu}_{15/16}$), 31.9 (3 x $CH_3$; $t\text{Bu}_{4/5}$, $t\text{Bu}_{8/9}$, $t\text{Bu}_{17/18}$), 31.4 (2 x $CH_3$; $t\text{Bu}_{25}$, $t\text{Bu}_{31}$), 31.3 (2 x $CH_3$; $t\text{Bu}_{29}$, $t\text{Bu}_{27}$), 31.0 ($CH_3$; $t\text{Bu}_{23}$), 30.8 ($CH_3$; $t\text{Bu}_{10}$), 30.4 ppm ($CH_3$; $t\text{Bu}_{19/20}$). *Note:* The assignment of CH and $CH_3$ signals is based on the DEPT and HSQC spectra. No further signals were assigned due to the complexity of the $^{13}$C and HMBC spectra. Several (x) overlapping signals are identified as xC.

HR-MS MALDI-TOF (m/z): calculated for $C_{164}H_{158}$ $[M]^+$, 2127.2358; found, 2127.2361, error = 0.14 ppm.

**Synthesis of the HBNG1**

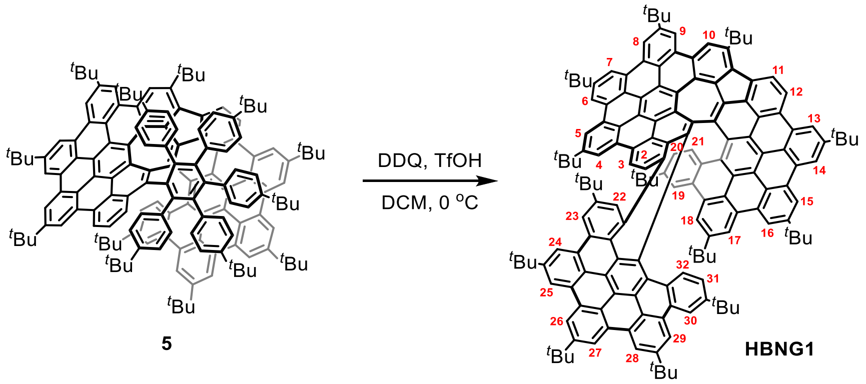


**5** (35.1 mg, 16.5 µmol) and DDQ (28.1 mg, 0.12 mmol) were dissolved in 20 mL of anhydrous DCM in a 50 mL Schlenk flask equipped with a magnetic stirrer under argon. The solution was cooled down to 0 °C with ice bath. After stirring for 5 min, 1 mL of trifluoromethanesulfonic acid (TfOH) was added dropwise through a syringe. The reaction mixture was kept stirred at 0 °C for 15 min and then quenched with 1 mL $Et_3N$. The reaction mixture was diluted with 20 mL DCM and the organic layer was washed with water, dried with anhydrous magnesium sulfate and concentrated under reduced pressure. The residue was subjected to flash chromatography (silica gel, isohexane/DCM=3/1) and used for Scholl reaction again under the same condition. Following the same procedure, compound **HBNG1** was obtained as a red solid in 31% yield.

$^1$H NMR (500 MHz, $CD_2Cl_2$): δ 9.34 (s, 1H; 4), 9.29 (s, 1H; 7), 9.28 (2H; 5, 6), 9.23 (s, 1H; 8), 9.16 (d, 8.3 Hz, 1H; 3), 9.07 (s, 1H; 17), 8.97 (s, 1H; 28), 8.90 (s, 1H; 15), 8.83 (s, 1H; 16), 8.79 (s, 1H; 14), 8.76 (s, 1H; 9), 8.63 (2H; 27, 29), 8.62 (d, 8.3 Hz, 1H; 21), 8.61 (s, 1H; 13), 8.60 (s, 1H; 10), 8.42 (s, 1H; 26), 8.33 (s, 1H; 18), 8.31 (d, 8.1 Hz, 1H; 11), 8.26 (s, 1H; 25),

8.23 (d, 8.3 Hz, 1H; 2), 8.23 (s, 1H; 30), 8.22 (d, 8.1 Hz, 1H; 12), 8.07 (s, 1H; 24), 7.83 (2H; 19, 23), 7.08 (s, 1H; 22), 6.72 (d, 8.8 Hz, 1H; 32), 5.74 (dd, 8.8 Hz, 2.0 Hz, 1H; 31), 5.16 (dd, 8.3 Hz, 2.0 Hz, 1H; 20), 2.19 (s, 9H; $t\mathrm{Bu}_{10}$), 1.95 (s, 9H; $t\mathrm{Bu}_{28/29}$), 1.91 (s, 9H; $t\mathrm{Bu}_{4/5}$), 1.89 (s, 9H; $t\mathrm{Bu}_{6/7}$), 1.78 (s, 9H; $t\mathrm{Bu}_{17/18}$), 1.77 (s, 9H; $t\mathrm{Bu}_{13/14}$), 1. 76 (s, 9H; $t\mathrm{Bu}_{8/9}$), 1.74 (s, 9H; $t\mathrm{Bu}_{15/16}$), 1.65 (s, 9H; $t\mathrm{Bu}_{22/23}$), 1.63 (s, 9H; $t\mathrm{Bu}_{26/27}$), 1.58 (s, 9H; $t\mathrm{Bu}_{24/25}$), 0.57 (s, 9H; $t\mathrm{Bu}_{30/31}$), 0.31 (s, 9H; $t\mathrm{Bu}_{19/20}$). *Note:* The signal assignment of aromatic proton signals is mainly based on sequences of ROESY and long-range COSY correlations. The ROESY correlations of the *tert*-butyl group signals to assigned aromatic proton signals allow their assignment. The ROESY correlations that are observed between protons of the core (1–21 and $t\mathrm{Bu}_{4/5}$ - $t\mathrm{Bu}_{19/20}$) and protons of the second layer (22–32 and $t\mathrm{Bu}_{22/23}$ - $t\mathrm{Bu}_{30/31}$) are in excellent agreement with the expectations from the crystal structure: 2 to 22, $t\mathrm{Bu}_{10}$ to 29 and 30, 16 and 17 to $t\mathrm{Bu}_{24/25}$, 18 to $t\mathrm{Bu}_{22/23}$ and 19 to 24.

$^{13}$C NMR (125 MHz, $CD_2Cl_2$): δ 150.2, 150.1, 149.8, 148.6, 148.5, 148.4, 148.3, 148.1, 148.0, 147.8 (2C), 147.7, 145.5, 143.4, 143.2, 141.8, 139.6, 138.6, 138.5, 138.1, 136.7, 135.4, 134.6 (22), 133.6, 132.5 (21, C), 132.2, 131.6, 130.9, 130.7 (2C), 130.6, 130.5, 130.4, 130.3, 130.2 (2C), 130.0 (3C), 129.9, 129.8, 129.6, 129.5, 129.3, 128.9 (32), 128.6 (2C), 128.1 (2), 127.7, 127.4, 127.0, 126.8 (11), 125.3, 125.2, 124.6, 124.4, 124.3, 124.1, 123.3, 123.1, 123.0, 122.9, 122.8, 122.7, 122.5 (31), 122.4, 122.1, 121.8, 121.7, 121.4 (10), 121.0 (2C), 120.8 (20), 120.2 (12), 119.9 (3), 119.7 (4), 119.6 (15), 119.5 (17), 119.4 (5 or 6), 119.3, 119.2 (5 or 6, 16), 119.1 (9), 119.0 (7), 118.8 (26), 118.7 (13), 118.6 (8), 118.5 (19 or 23), 118.4 (28), 118.2 (27 or 29), 118.1 (14), 118.0 (24), 117.9 (18, 27 or 29), 117.3 (25), 117.1 (30), 116.6 (19 or 23), 36.0, 35.9 (3C), 35.8 (3C), 35.5 (2C), 35.2 (2C), 34.3, 33.6 , 32.2 (2 x $CH_3$; $t\mathrm{Bu}_{13/14}$ , $t\mathrm{Bu}_{28/29}$), 32.1 (2 x $CH_3$; $t\mathrm{Bu}_{17/18}$, $t\mathrm{Bu}_{24/25}$), 32.0 (2 x $CH_3$; $t\mathrm{Bu}_{4/5}$, $t\mathrm{Bu}_{6/7}$) 31.9 (2 x $CH_3$; $t\mathrm{Bu}_{8/9}$, $t\mathrm{Bu}_{15/16}$), 31.8 ($CH_3$; $t\mathrm{Bu}_{26/27}$), 31.6 ($CH_3$; $t\mathrm{Bu}_{22/23}$), 31.3 ($CH_3$; $t\mathrm{Bu}_{10}$), 30.7 ($CH_3$; $t\mathrm{Bu}_{30/31}$), 30.1 ($CH_3$; $t\mathrm{Bu}_{19/20}$). *Note:* The assignment of CH and $CH_3$ signals is based on the DEPT and HSQC spectra. No further signals were assigned due to the complexity of the $^{13}$C and HMBC spectra. Several (x) overlapping signals are identified as xC.

HR-MS MALDI-TOF (m/z): calculated for $C_{164}H_{148}$ $[M]^+$, 2117.1576; found, 2117.1604, error = 1.32 ppm.

## 3. High Resolution (HR) MALDI-TOF mass spectra of HBNG1

### 3.1 MALDI-TOF mass spectra of Scholl reaction from 5

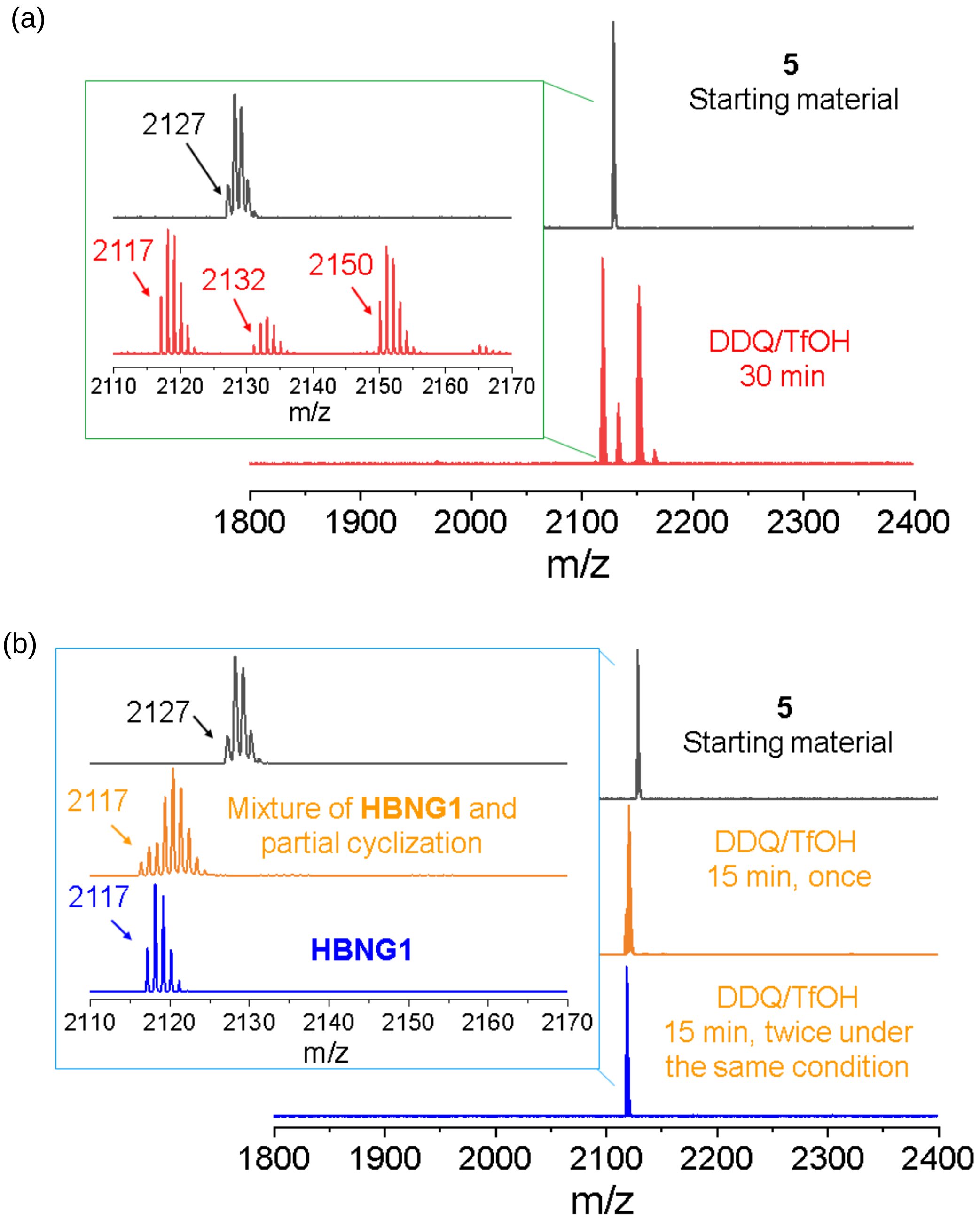


**Figure S1**. MALDI-TOF mass spectra of the Scholl reaction from **5** under different reaction conditions: (a) DDQ, TfOH, DCM, 0 ºC, 30 min; (b) DDQ, TfOH, DCM, 0 °C, 15 min, repeat two times. The targeted **HBNG1** has m/z = 2117.

### 3.2 HR-MALDI-TOF mass spectrum of HBNG1

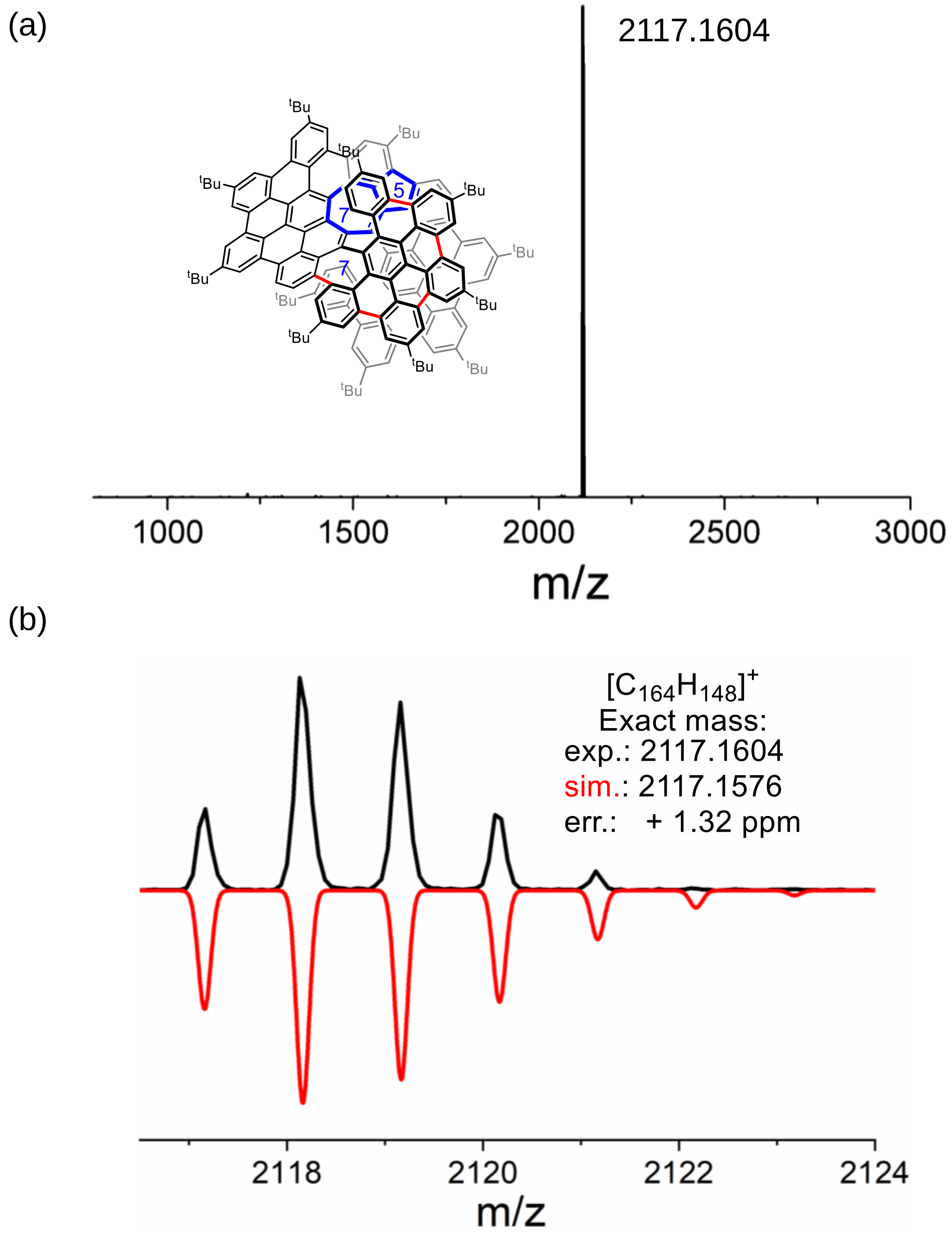


**Figure S2**. HR MALDI-TOF mass spectrum of **HBNG1**: (a) full spectrum; (b) isotope pattern.

## 4. NMR spectra of HBNG1

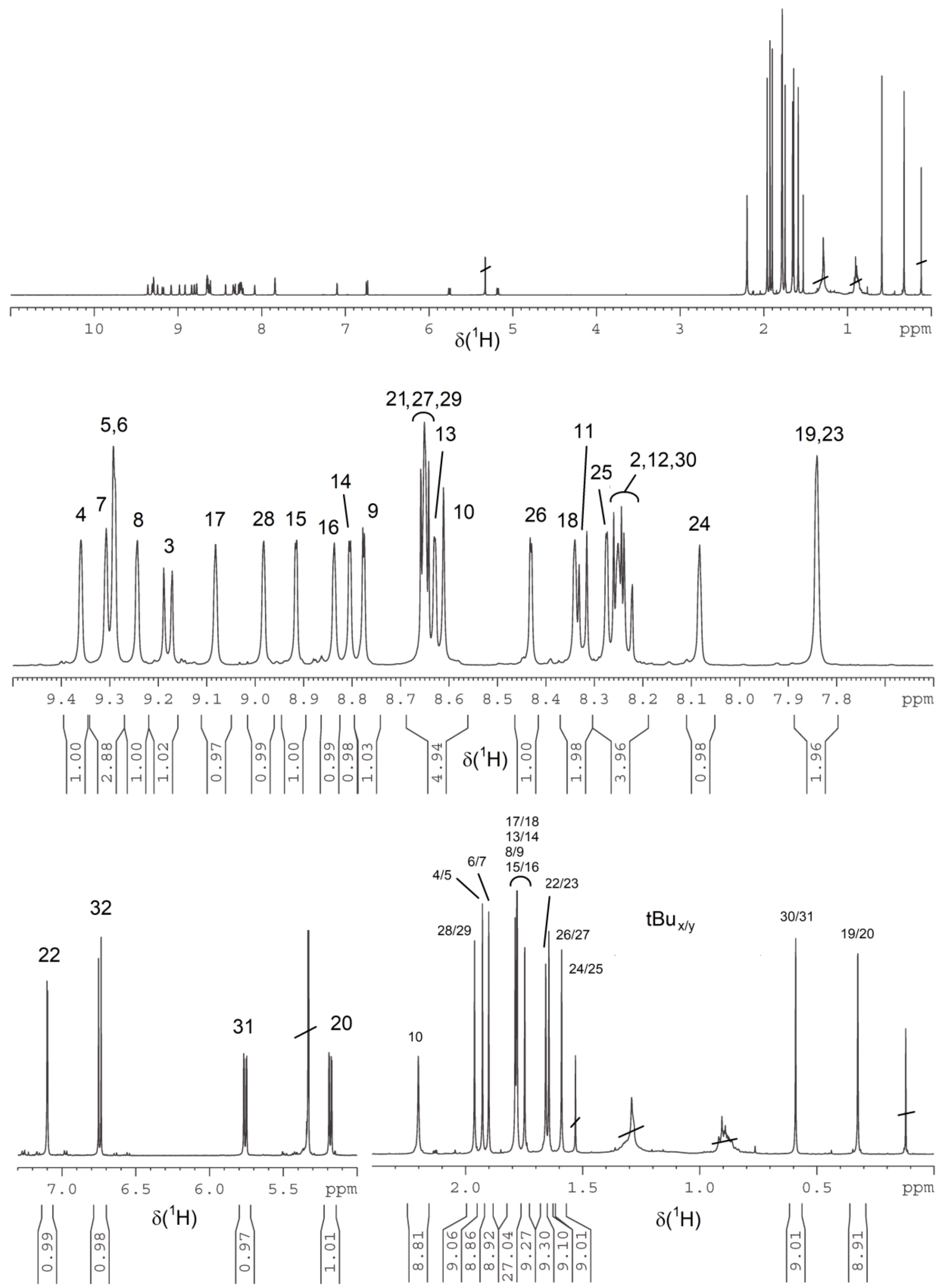


**Figure S3**. $^{1}H$ NMR spectrum (overview and regions) of **HBNG1** ($CD_2Cl_2$).

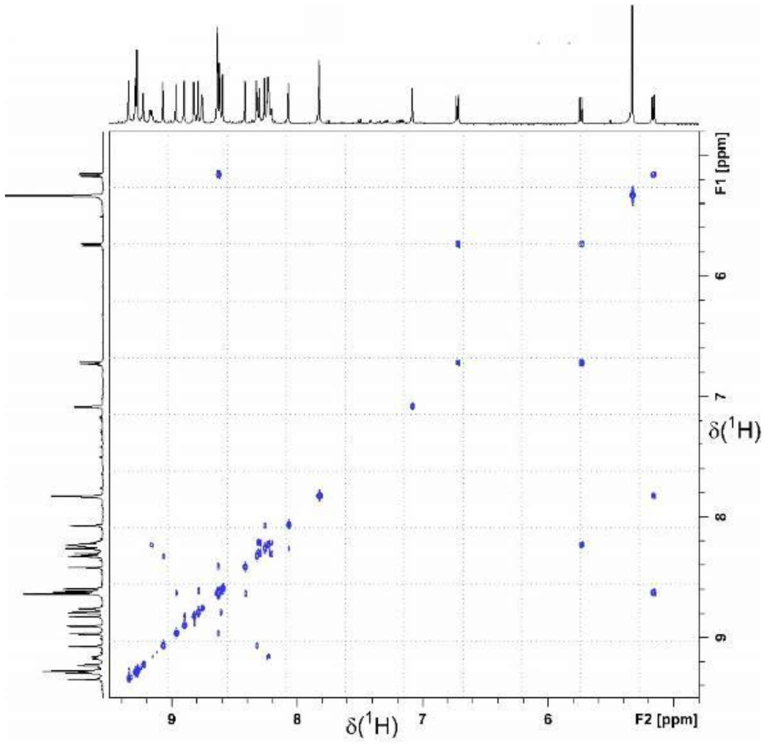


**Figure S4**. COSY spectrum (region) of **HBNG1** ($CD_2Cl_2$).

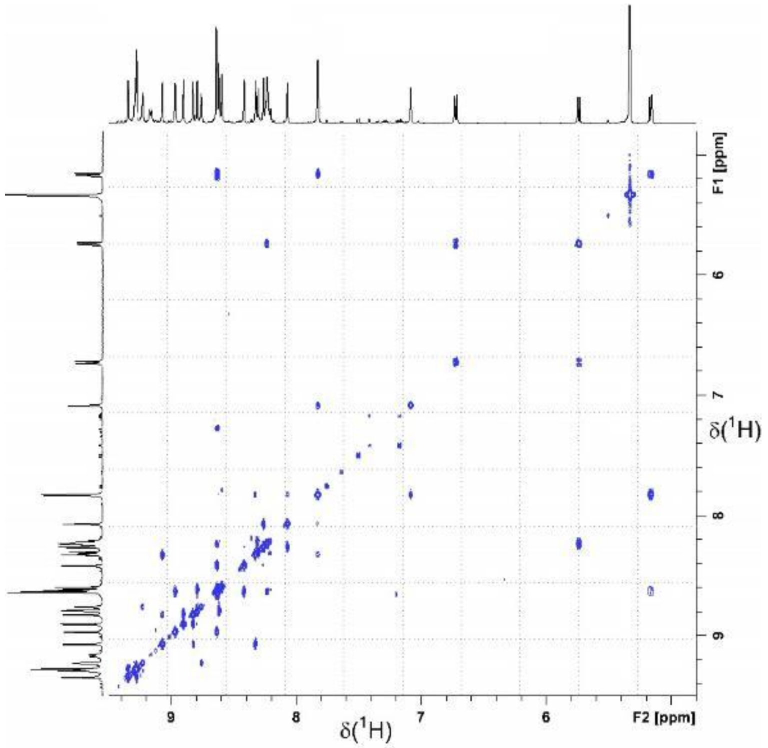


**Figure S5**. Long-range COSY spectrum (region) of **HBNG1** ($CD_2Cl_2$).

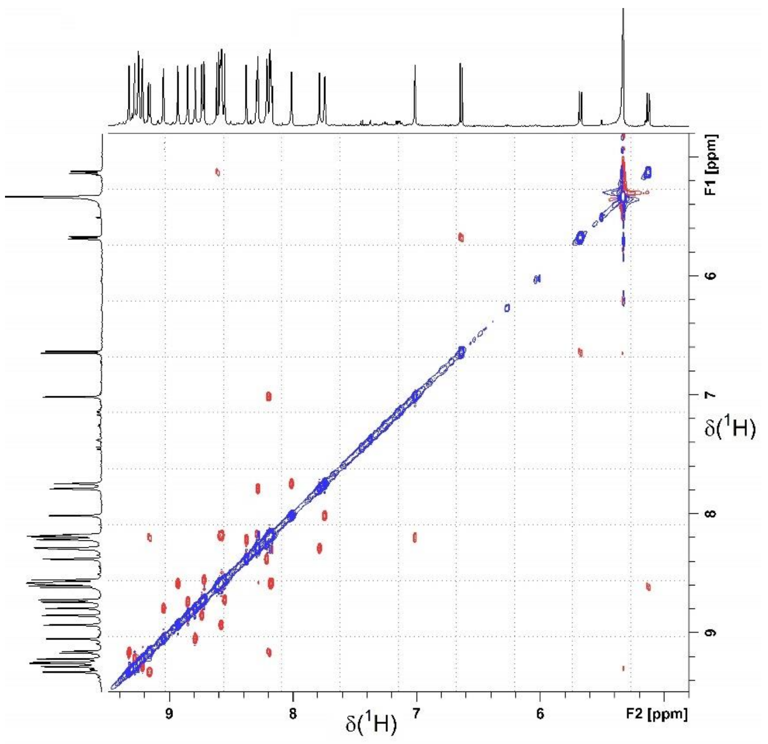


**Figure S6**. ROESY spectrum (region) of **HBNG1** ($CD_2Cl_2$).

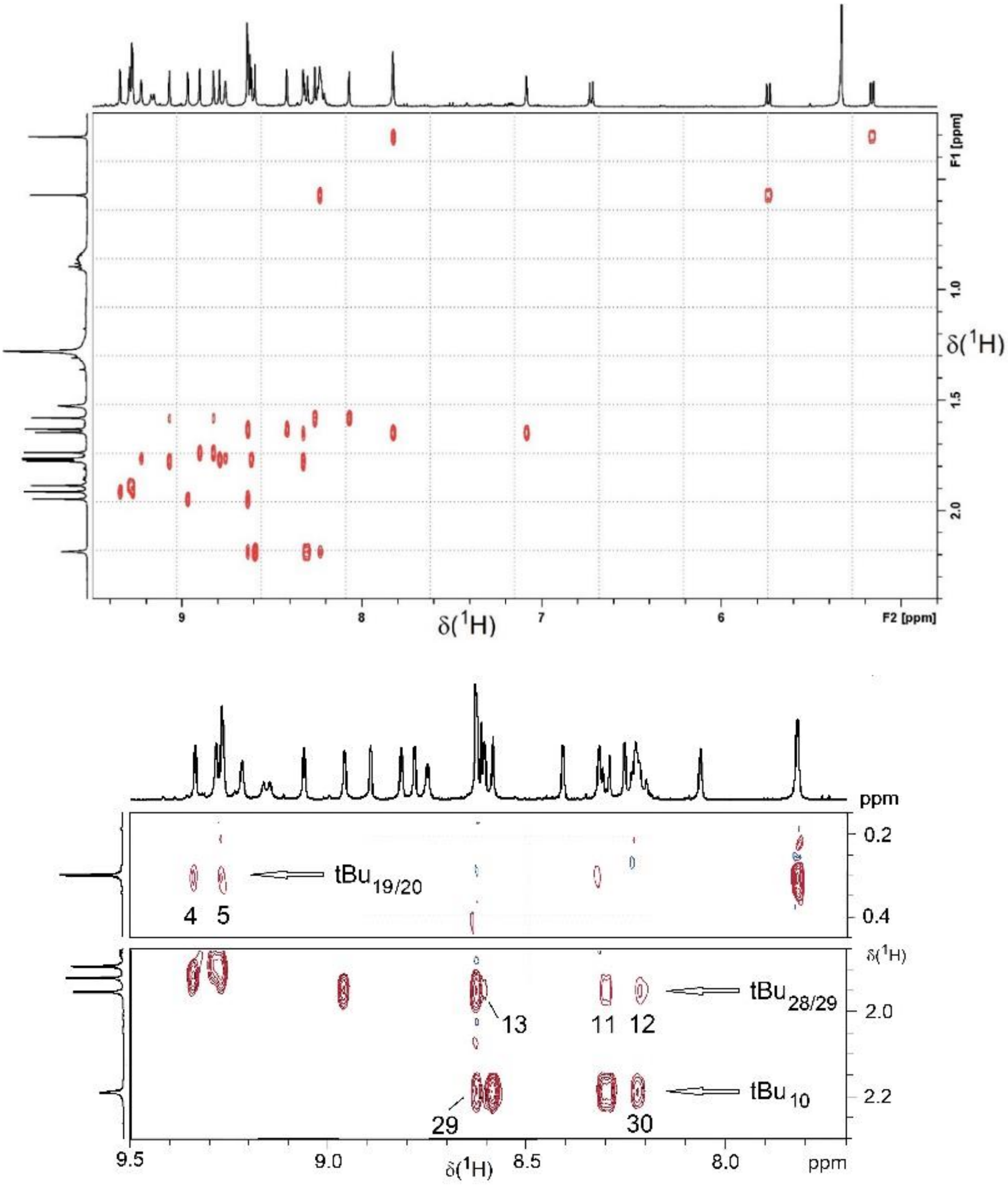


**Figure S7**. ROESY spectrum (correlations of *t*Bu groups) and regions from the ROESY spectrum of **HBNG1** ($CD_2Cl_2$) with assigned correlations between protons of spatial proximity but not structural neighbourhood. The intensities correlate well with the H-H distances determined from the X-ray structure: *t*Bu$_{19/20}$ **…** 4 (0.45 nm), **…** 5 (0.52 nm); *t*Bu$_{28/29}$ **…** 11 (0.28 nm), **…** 12 (0.31 nm), **…** 13 (0.44 nm) and *t*Bu$_{10}$ **…** 29 (0.26 nm), **…** 30 (0.27 nm).

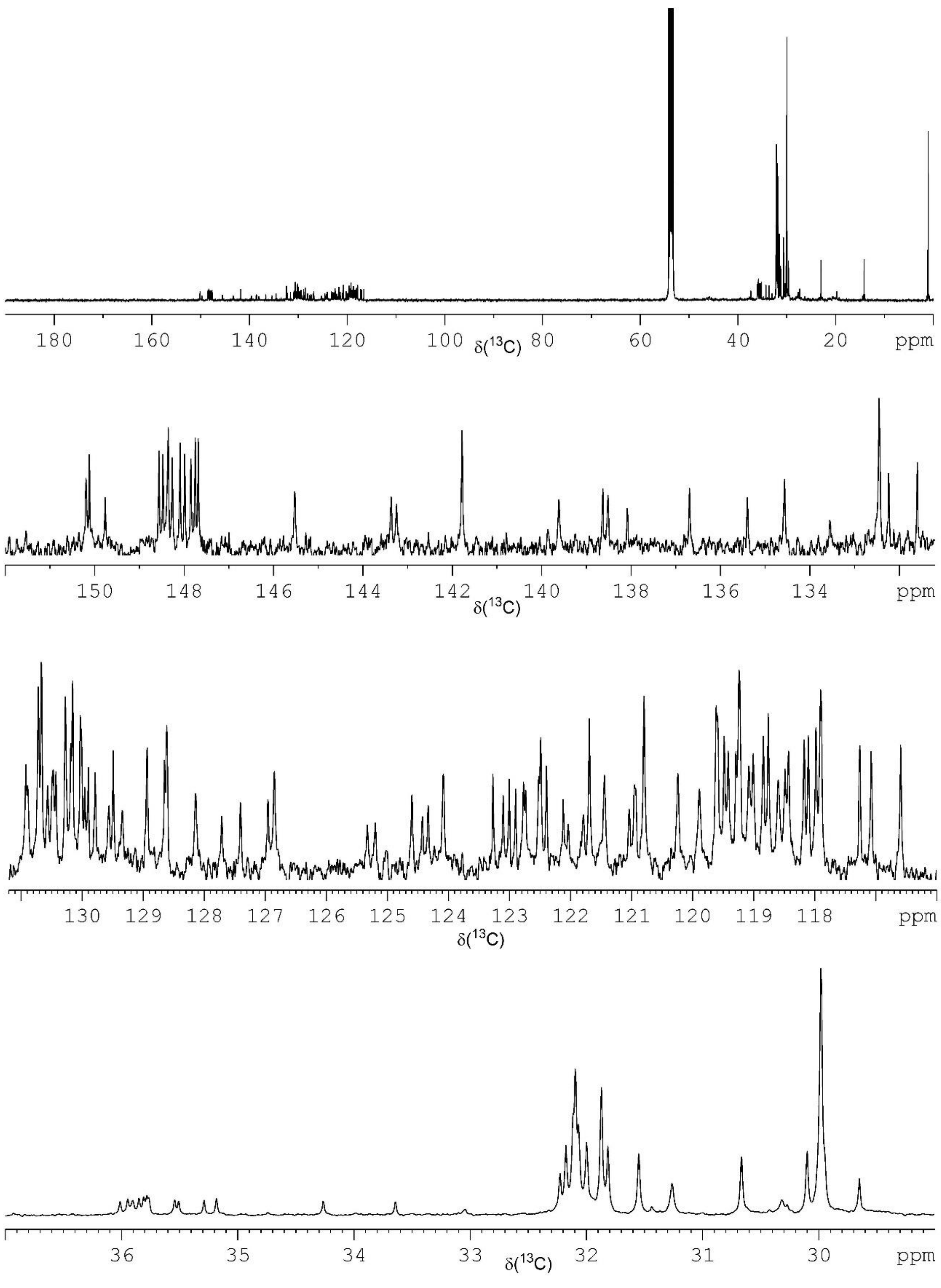


**Figure S8**. $^{13}$C NMR spectrum (overview and regions) of **HBNG1** ($CD_2Cl_2$).

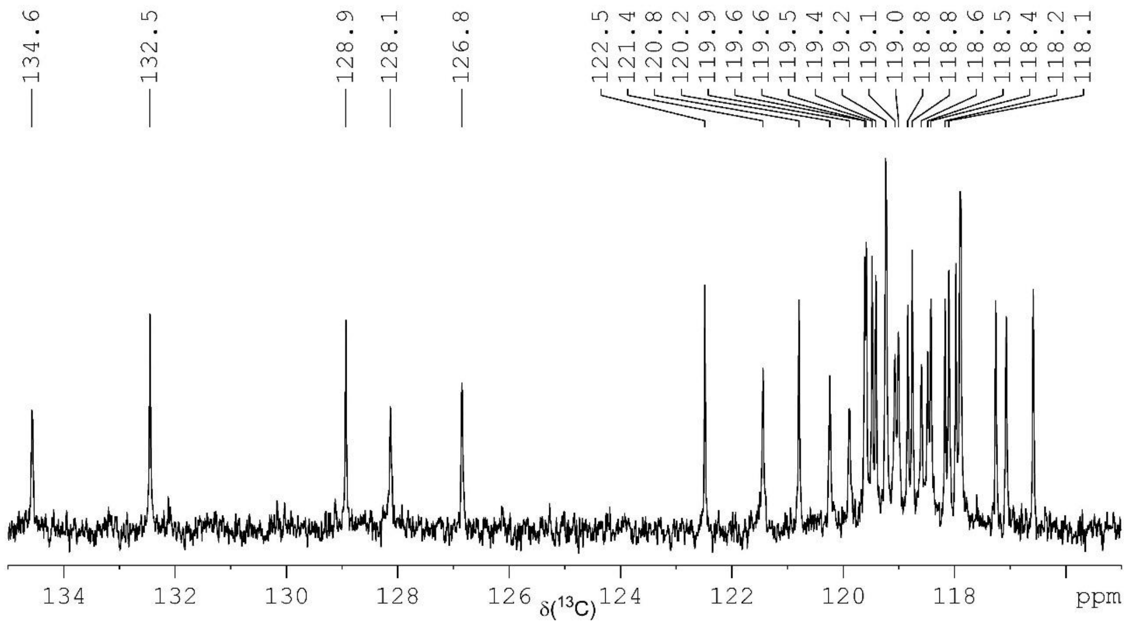


**Figure S9**. DEPT135 spectrum (region of aromatic CH groups) of **HBNG1** ($CD_2Cl_2$).

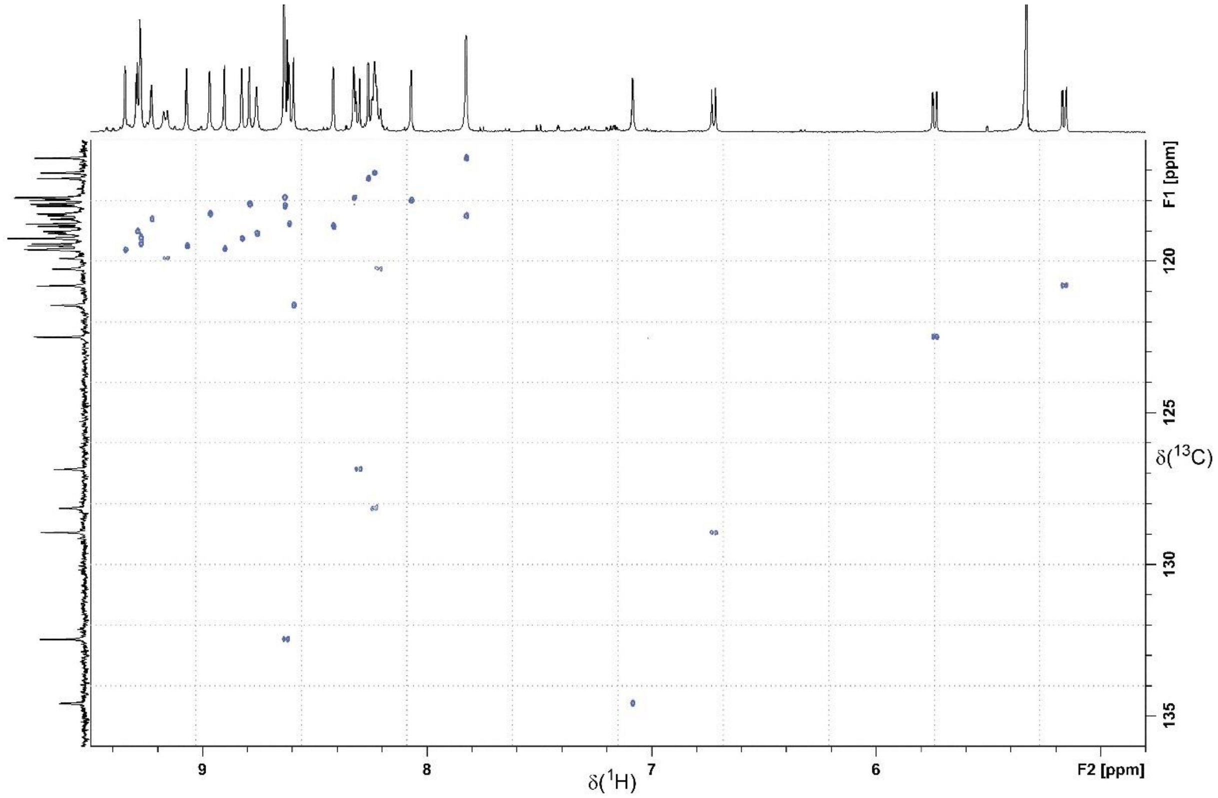


**Figure S10**. HSQC spectrum (correlations of aromatic CH groups) of **HBNG1** ($CD_2Cl_2$).

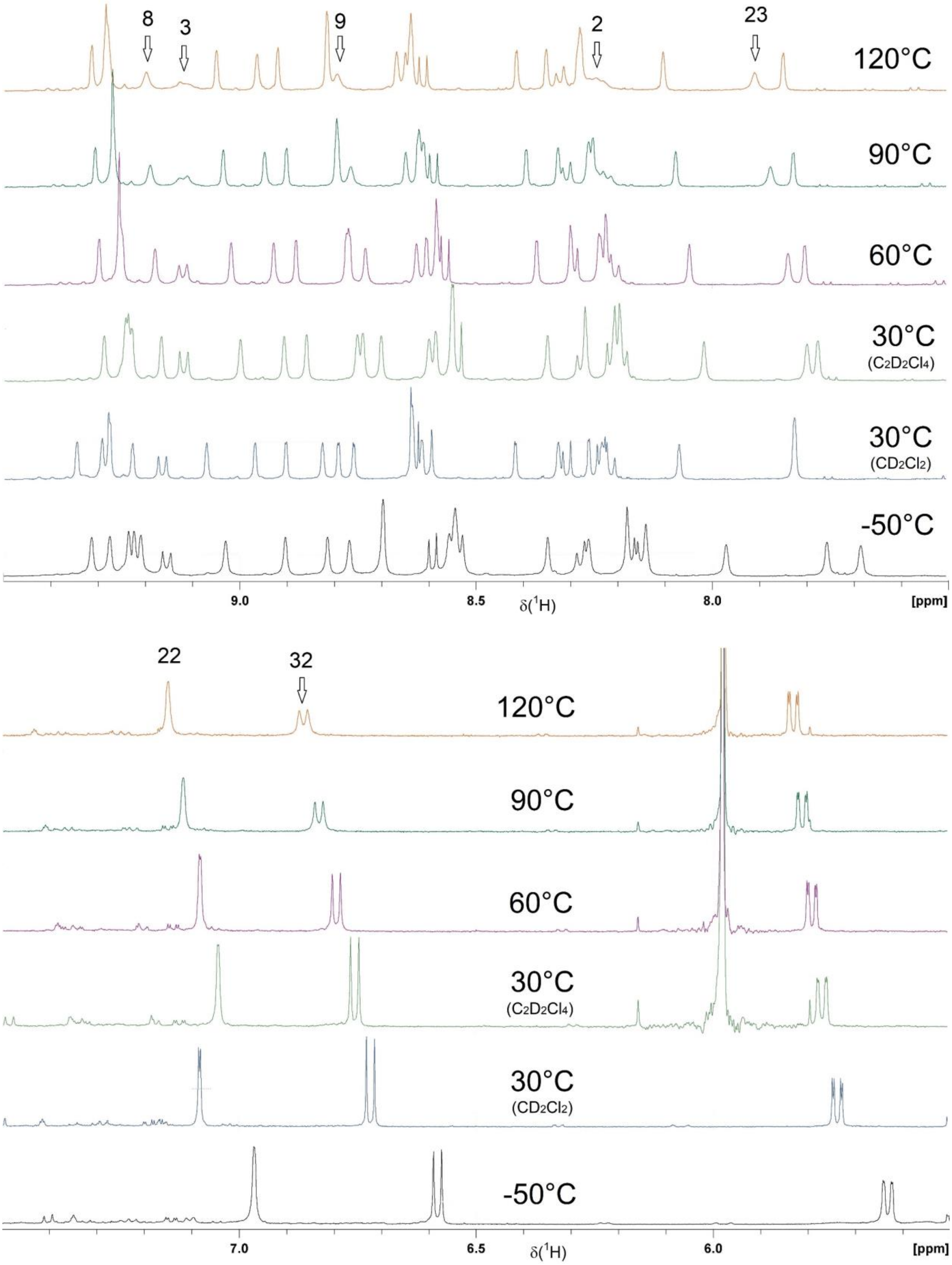


**Figure S11**. $^1$H NMR spectra (region of aromatic protons) of **HBNG1** recorded at different temperatures in $CD_2Cl_2$ (30 °C, -50 °C) and in $C_2D_2Cl_4$ (30 °C, 60 °C, 90 °C, 120 °C). *Note:* In addition to minor signal shifts due to temperature and solvent effects, line broadening with temperature increase is observed for some signals.

## 5. X-ray crystallographic analysis of HBNG1

Single crystals of **HBNG1** were obtained by slow vapor diffusion of the methanol into chlorobenzene. A suitable crystal was selected and mounted on a XtaLAB Synergy, Dualflex, HyPix diffractometer. The crystal was kept at 100.00(10) K during data collection. Using Olex2[2], the structure was solved with the ShelXT[3] structure solution program using Intrinsic Phasing and refined with the ShelXL[3] refinement package using Least Squares minimisation.

Crystal Data for **HBNG1**: tetragonal, space group $I4_1/a$, $a$ = 51.3988(5) Å, $c$ = 23.5569(7) Å, $V$ = 62233(2) $Å^3$, $Z$ = 16, $T$ = 100.00(10) K, 84809 reflections measured (6.38° ≤ 2θ ≤ 129.98°), 25640 unique ($R_{int}$ = 0.0406, $R_{sigma}$ = 0.0411) which were used in all calculations. The final $R_1$ was 0.0747 (I > 2σ(I)) and $wR_2$ was 0.2314 (all data).

The X-ray crystallographic coordinates for **HBNG1** has been deposited at the Cambridge Crystallographic Data Centre (CCDC 2191753). These data can be obtained free of charge from CCDC via http://www.ccdc.cam.ac.uk/data_request/cif

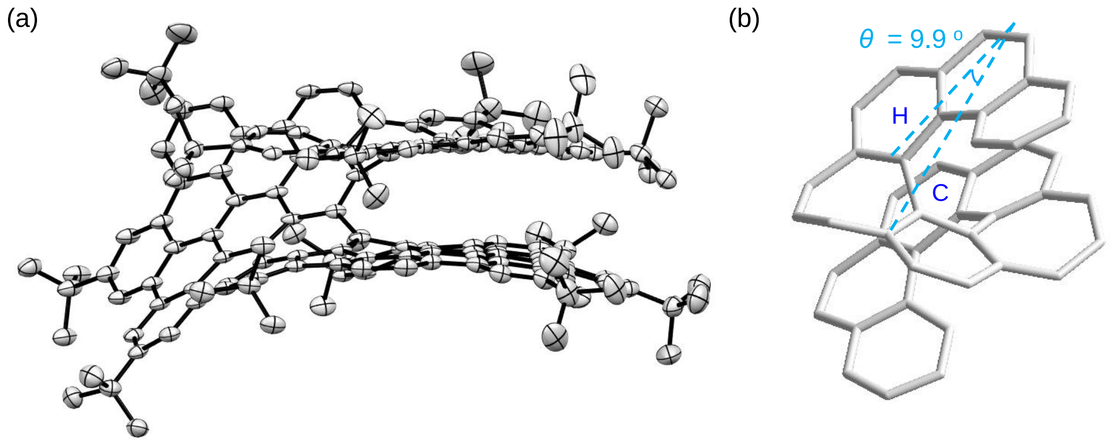


**Figure S12**. (a) X-ray crystallographic structure (ORTEP drawing with 50% thermal ellipsoids) of **HBNG1**. (b) The dihedral angle between the centroid rings C and H.

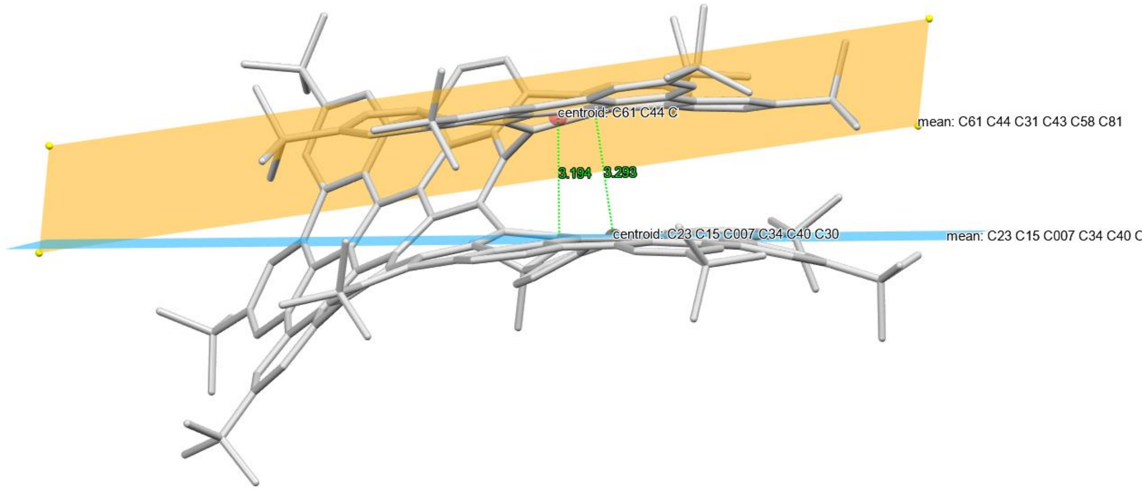


**Figure S13**. The average perpendicular distance between the centroid of rings C and H in **HBNG1**. The average perpendicular distance is the distance between the centroid of ring C and the plane of ring H, as well as the distance between the centroid of ring H and the plane of ring C.

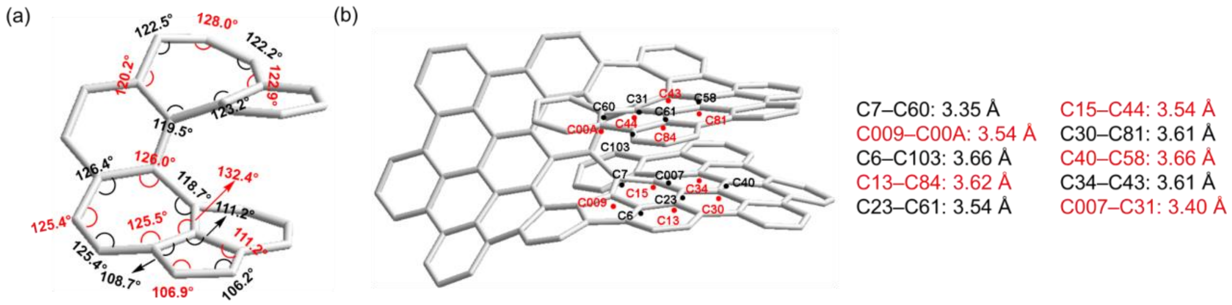


**Figure S14**. (a) The azulene and heptagon's C–C–C bond angles. (b) The C-C bond distance between the close two benzenoid rings.

## 6. Determination of Enantiopurity HBNG1 by HPLC

Many conditions were tested (Table S1). Finally, the racemic resolution of **HBNG1** was achieved using a Chiralpak® IA analytical chiral column packed with Cellulose tris(3,5-dimethylphenylcarbamate) immobilized on 5 μm silica gel. The column temperature was set at 20 °C and the flow rate was constant during operation (0.8 mL/min).

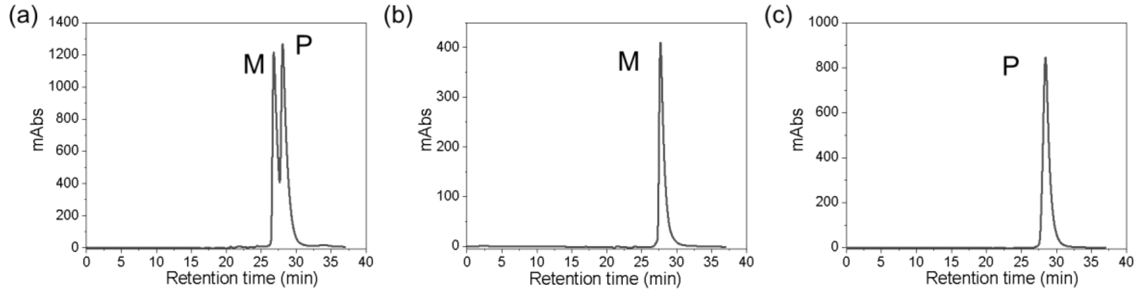


**Figure S15**. (a) Chiral HPLC trace of **HBNG1** during the separation (9:1 Hex:DCM, chiralpak® IA, 0.8 mL/min); (b, c) Chiral HPLC traces of the reinjected fractions 1 (*M*) and 2 (*P*).

**Table S1.** Detailed conditions for the chiral HPLC separation.

| Column | Entry | Mobile phase | Flow rate (mL/min) | Result |
|---|---|---|---|---|
| Chiralpak® IC (5 µm) | 1 | 95:5 Hex:EtOAc | 1 | 3.4 min, symmetric single peak |
| | 2 | 100:0->95:5->100:0 Hex:EtOAc | 1 | 6.1 min, broad peak |
| | | | | 7.5 min, tailing peak |
| | 3 | 9:1 Hex:DCM | 1 | 3.5 min, narrow tall peak |
| | | | | 4.7 min, tailing peak |
| | 4 | 9:1 Hex:DCM | 0.5 | 10.7 min, shoulder peak |
| | 5 | 100:0->7:3->100:0 Hex:DCM | 1 | 9.4 min, shoulder peak |
| | 6 | 100:0->1:1->100:0 Hex:DCM | 1 | 7.0 min, shoulder peak |
| | 7 | 100:0->9:1->100:0 Hex:DCM | 1 | 12.7 min and 13.4 min, two overlapping peaks |
| Chiralpak® IA (5 µm) | 8 | 7:3 Hex:DCM | 3.6 | 3.1 min, symmetric single peak |
| | 9 | 9:1 Hex:DCM | 3.6 | 5.1 min, symmetric single peak |
| | 10 | 100 Hex | 3.6 | 4.0 min, narrow peak |
| | | | | 25 min, broad peak |
| | 11 | 95:5 Hex:EtOAc | 3.6 | 5.7 min, tall single peak |
| | | | | 6.9 min, short single peak |
| | 12 | 9:1 Hex:EtOAc | 3.6 | 5.4 min, tailing peak |
| | 13 | 100:0->9:1->100:0 Hex:DCM | 1 | 20.1 min, symmetric single peak |
| | 14 | 100:0->9:1->100:0 Hex:DCM | 3 | 12.8 min, two merged peaks |
| | 15 | 100:0->9:1->100:0 Hex:DCM | 3.6 | 11.4 min, two merged peaks |
| | **16** | **9:1-Hex:DCM** | **0.8** | **27 min, two isolated peaks** |
| (R,R)-Whelk-O (15 µm) | 17 | 8:2 Hex:$CHCl_3$ | 10 | 11 min, two merged peaks |
| | 18 | 9:1 Hex:$CHCl_3$ | 10 | 27 min, two merged peaks |
| | 19 | 8:2 Hex:$CHCl_3$ | 8 | 13 min, two merged peaks |
| | 20 | 8:2 Hex:$CHCl_3$ | 6 | 18 min, two merged peaks |
| | 21 | 85:15 Hex:Toluene | 10 | 17 min, two merged peaks |
| | 22 | 95:5 Hex:EtOH | 10 | 13 min, two merged peaks |
| | 23 | 89:10:1 Hex:$CHCl_3$:EtOH | 10 | 16 min, two merged peaks |

## 7. Photophysical study

### 7.1 Fluorescence spectrum of HBNG1

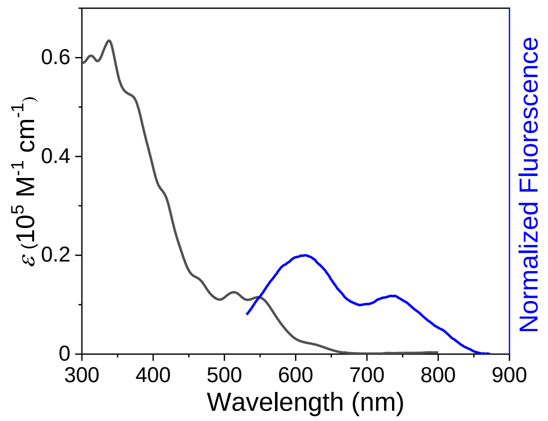


**Figure S16**. UV-vis absorption (black line) and photoluminescence emission (blue line, excitation at 510nm) spectra of **HBNG1** in DCM.

### 7.2 Molar extinction coefficient of HBNG1

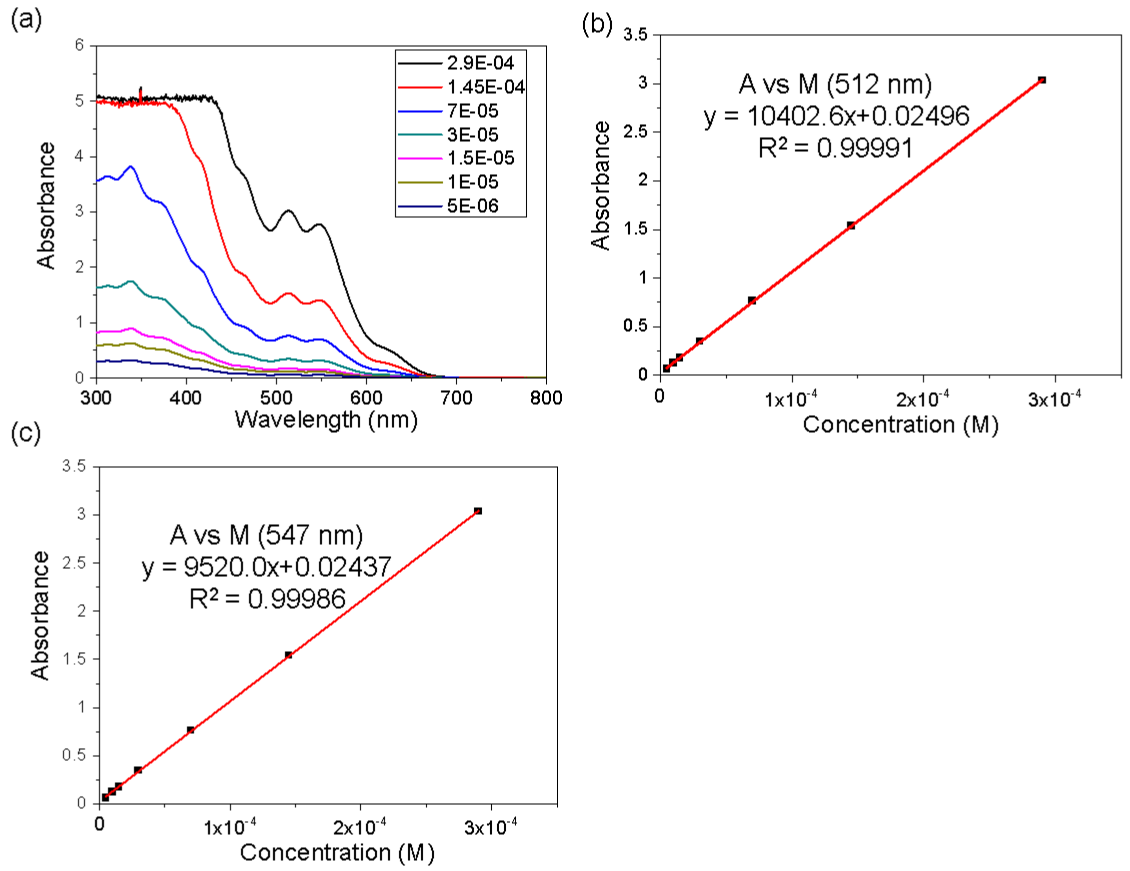


**Figure S17**. (a) Absorbance spectra of **HBNG1** at different concentrations (in M) in DCM. Absorbance vs concentration plot of compound **HBNG1** at ~512 nm (b) and ~547 nm (c), respectively.

### 7.3 Electronic circular dichroism and circularly polarized luminescence measurements of HBNG1

The spectra were recorded at *ca.* $1.6\times10^{-5}$ M (for *P*) and *ca.* $1.2\times10^{-5}$ M (for *M*) in HPLC grade DCM at 20 °C. For ECD measurements a fixed slit-width of 1 mm, 0.1 s of integration time and 50 scans were selected. For CPL measurements, a fixed wavelength of 525 nm provided by a LED source and 1.0 s of integration time was selected, the CPL spectra shown in Figure S18b are average spectra calculated after 100 scans at 0 °C.

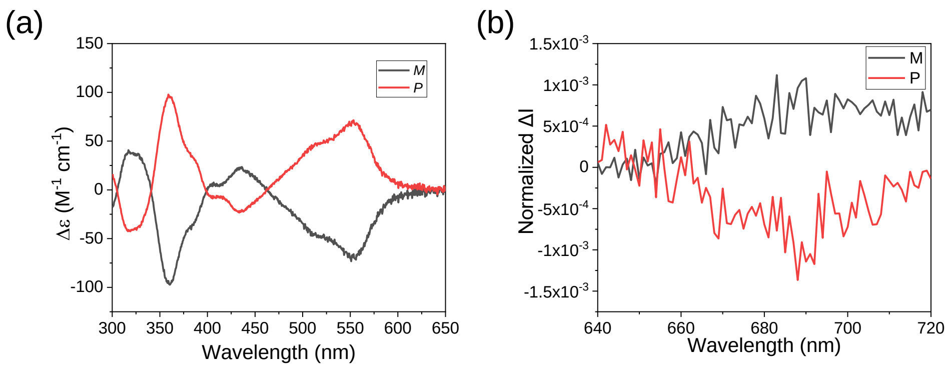


**Figure S18**. Experimental ECD spectra (a) and CPL spectra (b) of *P*- (red) and *M*- (black) **HBNG1** irradiating at 525 nm.

## 8. Configurational stability study of HBNG1 by VT-ECD and HPLC

The value of the minimum Gibbs activation energy ($\Delta G^{\ddagger}(T)$) for enantiomerization of **HBNG1** was obtained by following the decay of the ECD signal over time of the enantiopure solution of *P*-**HBNG1** in toluene at 100 ºC (± 0.2 ºC).

Remarkably, compound **HBNG1** resists racemization at 100 ºC over 4 h. No hints of racemization or decomposition were observed as neither ECD nor UV signal decreases (Figure S19a).

The *P*-**HBNG1** was heated as hexadecane solution at 473 K for 2 h and monitored by chiral HPLC. However, the decomposition of *P*-**HBNG1** is observed, as indicated by the complicated HPLC curve in Figures S19b and 19c.

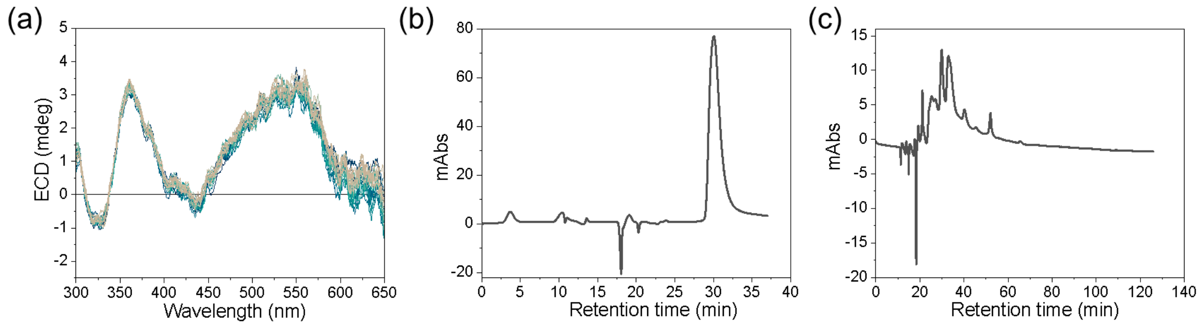


**Figure S19**. (a) ECD spectra of *P*-**HBNG1** in toluene at 100 ºC over 4 h. Chiral HPLC analysis of *P*-**HBNG1** before (b) and after (c) heated at 200 °C in hexadecane solution for 2 hours (Column: chiralpak® IA, eluent: Hex/DCM=9/1, elution rate: 0.8 mL/min).

## 9. CV and *in situ* spectroelectrochemistry of HBNG1

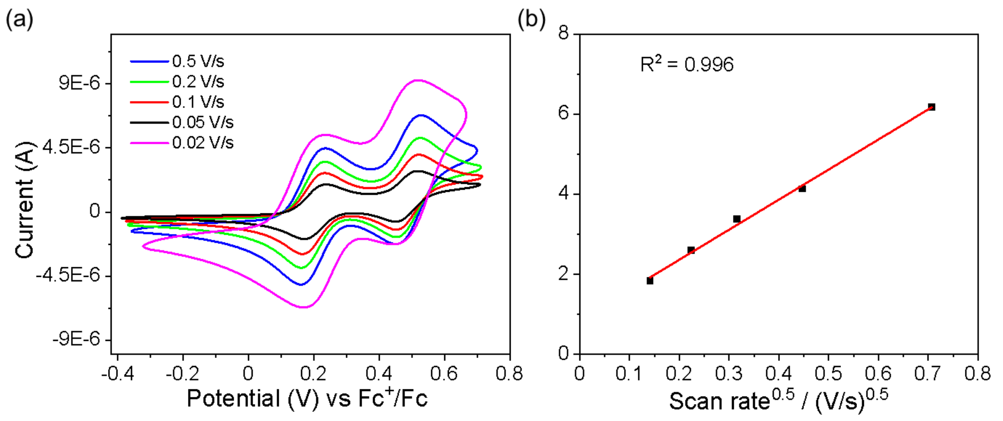


**Figure S20**. (a) CV of **HBNG1**'s partial oxidative region in DCM containing 0.1 M *n*$Bu_4NPF_6$ against $Fc^+$/Fc at different scan rates at room temperature. (b) Dependence of the peak current (first oxidation) on the square root of the scan rate.

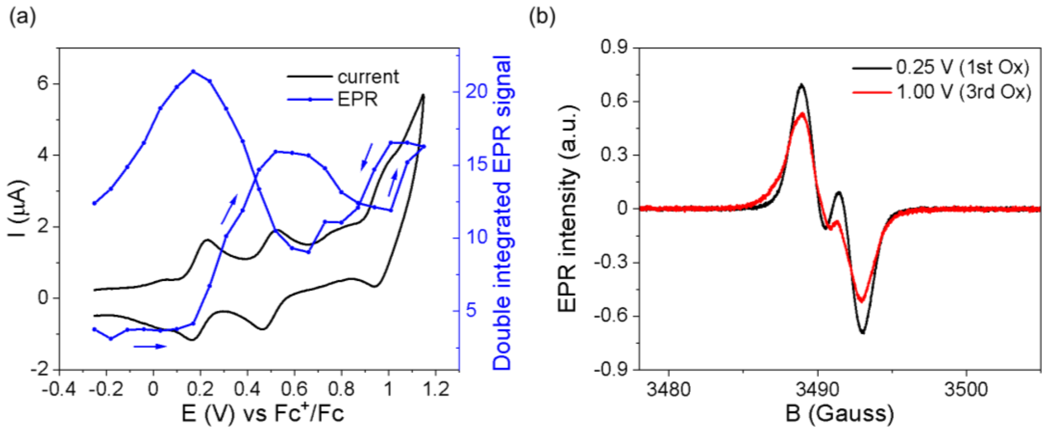


**Figure S21**. (a) The CV of **HBNG1** and the intensity of its EPR signal during the voltammetric cycle. (b) EPR spectrum of radical cation during the first oxidation and radical trication during the third oxidation of **HBNG1**.

## 10. DFT calculations

All density functional theory (DFT) calculation was performed using the Gaussian 16 program. [4] The B3LYP functional was used for geometry optimization in the ground. All the geometry optimization was done in the gas phase and based on the single crystal structure. In order to simulate the UV-Vis spectra of the molecules TD-DFT calculations using B3LYP functional. For better comparison to the experimental absorption spectra the polarity of the solvent dichloromethane was added.

Anisotropy of the induced current density (ACID) plots were calculated by Herges's method. [5] Nucleus independent chemical shifts (NICS) values were calculated using the standard gauge invariant atomic orbital (GIAO)[6] method at B3LYP functional. All NICS values were averaged by two positions (above and below the plane) of each molecule. The noncovalent interactions were investigated via reduced density gradient (RDG) using Multiwfn software.[7]

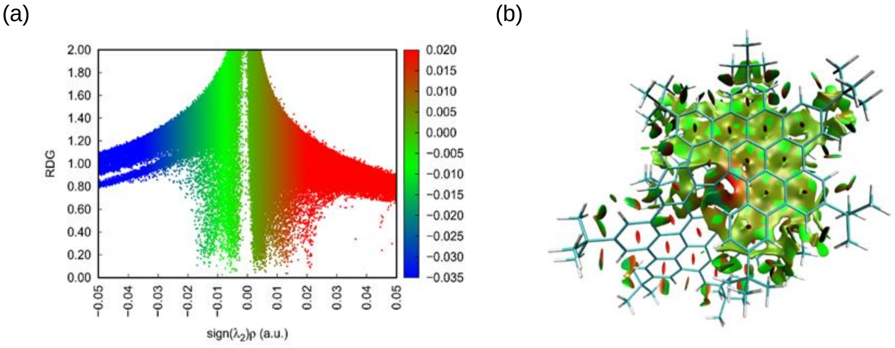


**Figure S22**. (a) Graphical representation of the reduced density gradient (RDG) *versus* sign($\lambda_2$)$\rho$, in which the blue, green, and red color of the vertical scale represent the hydrogen bonding, Van der Walls effect, and steric effect, respectively. (b) Plots of the reduced density gradient isosurfaces for **HBNG1**.

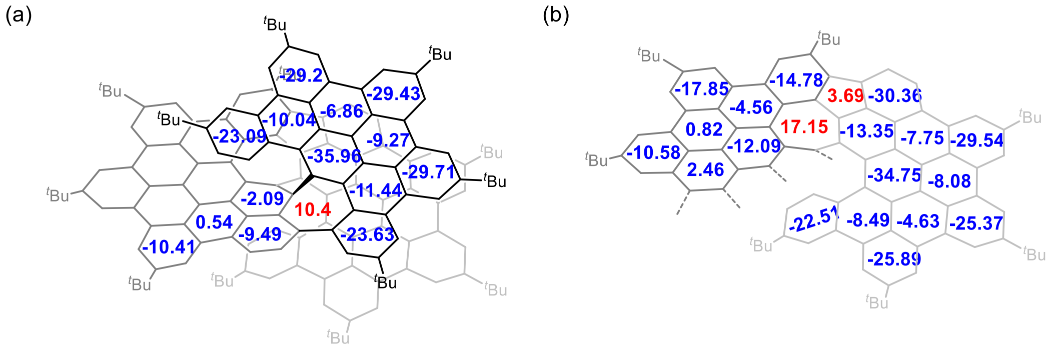


**Figure S23**. NICS(1)$_{zz}$ values of **HBNG1** at the top layer (a) and bottom layer (b), calculated at the GIAO-B3LYP/6 31+G(2d,p) level of theory.

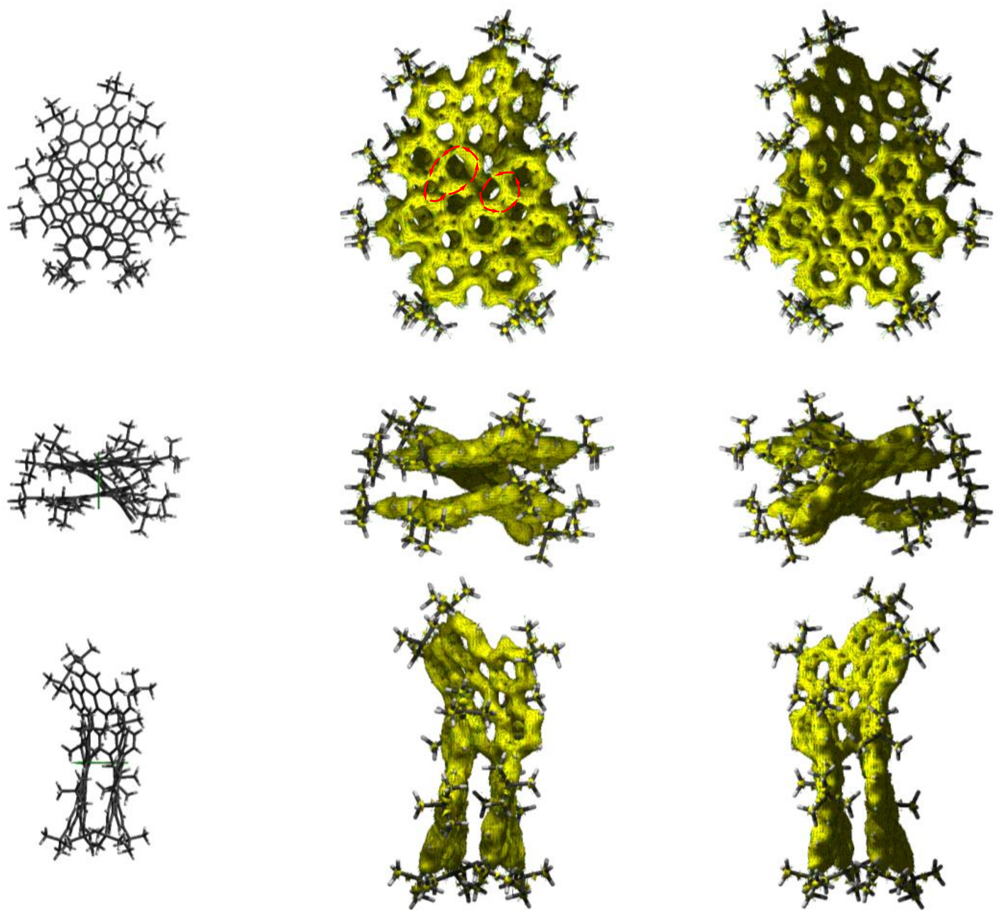

**Figure S24**. ACID plots of **HBNG1** viewed from different angles. The green arrow indicates the magnetic field. The heptagon's counter-clockwise ring current is obscured because it is blocked by the upper layer. Isosurface value is 0.05.

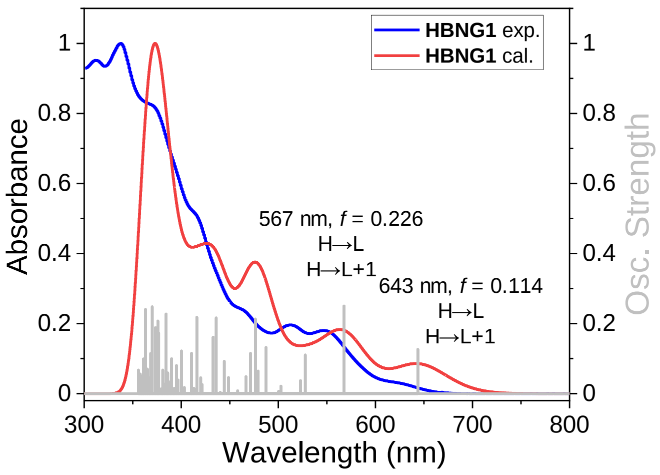


**Figure S25**. Comparison of experimental (blue), TD-DFT calculated (red) UV-Vis spectra and calculated oscillator strength (grey bar) of **HBNG1** along with assignments of key transitions at TD-B3LYP/6-31+G(2d,p) level of theory. H = HOMO, L = LUMO, *f* = oscillator strength.

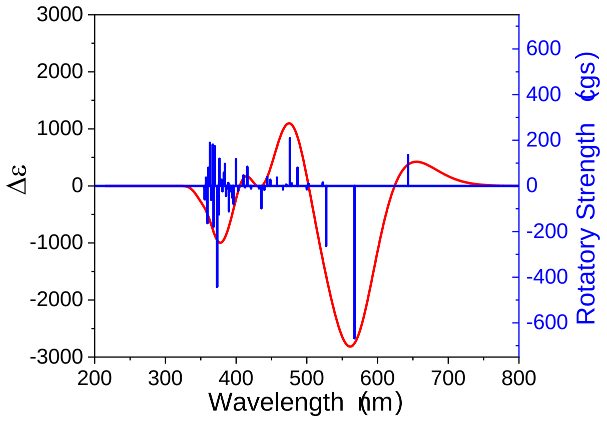


**Figure S26**. Calculated CD spectrum of ***M*-HBNG1** in DCM at TD-B3LYP/6-31+G(2d,p) level of theory.

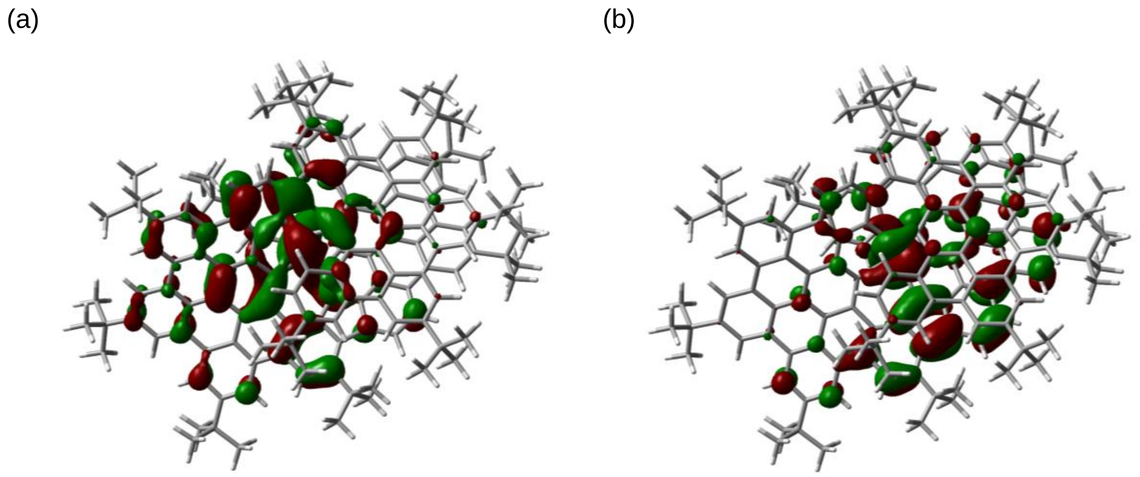


**Figure S27**. Frontier molecular orbitals of **HBNG1** (a) HOMO (b) LUMO. The calculations were performed at the B3LYP 6-31G (d) level. Notably, the calculated HOMO (−4.75 eV) and LUMO (−2.32 eV) of **HBNG1** are separated by the azulene due to the intramolecular electron transfer.[8]

**Table S2**. The ground state and intervalence charge transfer parameters for radical cation are calculated using a 35% HF-exchange contribution. TDDFT calculations were carried out in dichloromethane.

| | *E*ab / $cm^{-1}$ | $\Delta\mu$ab / D | $\mu$ab / D | $V_{12}$ / $cm^{-1}$ |
|---|---|---|---|---|
| **NHBG1** | 25805 | 3.14 | 4.03 | 13280 |

$\Delta\mu$ab: dipole moment difference between ground sate and IV-CT state, μab: projection of transition moment on dipole moment difference vector $\Delta\mu$ab.

**Table S3**. TD-DFT calculation of **HBNG1** using B3LYP functional and 6-31G(d) basis set.

| Excited state | Energy (eV) | Wavelength (nm) | Oscillator strength (f) | Description |
|---|---|---|---|---|
| 1 | 1.9274 | 643.27 | 0.1140 | HOMO->LUMO 90.7%<br>HOMO->LUMO +1 6.6% |
| 2 | 2.1854 | 567.33 | 0.2259 | HOMO->LUMO +1 89.6%<br>HOMO->LUMO 5.6% |
| 3 | 2.3508 | 527.41 | 0.0995 | HOMO-2->LUMO 90.2% |
| 4 | 2.3728 | 522.52 | 0.0336 | HOMO-1->LUMO 94.5% |
| 5 | 2.4681 | 502.35 | 0.0191 | HOMO-3->LUMO 35.7%<br>HOMO->LUMO +2 26.1%<br>HOMO-5->LUMO 17.2%<br>HOMO-4->LUMO 7.8% |
| 6 | 2.4800 | 499.94 | 0.0061 | HOMO->LUMO +2 55.1%<br>HOMO-3->LUMO 21.5%<br>HOMO->LUMO +3 5.6% |
| 7 | 2.5459 | 487.00 | 0.1192 | HOMO-5->LUMO 55.4%<br>HOMO-3->LUMO 29.2%<br>HOMO-4->LUMO 5.1% |
| 8 | 2.5893 | 478.83 | 0.0582 | HOMO-1->LUMO+1 41.5%<br>HOMO-4->LUMO 30.7%<br>HOMO->LUMO +3 11.9% |
| 9 | 2.6045 | 476.04 | 0.1925 | HOMO-1->LUMO+1 33.1%<br>HOMO-2->LUMO+1 30.2%<br>HOMO-4->LUMO 23.1% |
| 10 | 2.6324 | 470.99 | 0.1039 | HOMO-2->LUMO+1 63.7%<br>HOMO-4->LUMO 12.3%<br>HOMO-1->LUMO+1 7.8% |

## 11. Structure and property comparison of 2 and HBNG1

Compared to compound **2**, the UV-Vis absorption of **HBNG1** showed a slightly red-shift absorption band around 628 nm and a long tail up to 680 nm (Figure S28) due to the extended π-conjugation or the possible interlayer charge transfer. Consequently, the optical energy gap of **HBNG1** (1.86 eV) is smaller than compound **2** (2.03 eV). Apart from the optical properties, the introduction of an additional QHBC unit in **HBNG1** induced a very different electrochemical behavior in comparison to **2**, and two more reversible oxidation waves are observed in **HBNG1**, which probably come from the QHBC unit. In addition, the **HBNG1** has higher HOMO/LUMO (−4.9/−3.01 eV) levels than that of compound **2** (−5.18/−3.24 eV), giving the **HBNG1** (1.89 eV) with a smaller electrochemical energy gap than compound **2** (1.94 eV), in agreement with the trend of the optical energy gap. Regarding the ring current tropicities, for both compounds, ACID calculations exhibit clockwise ring current for hexagonal rings, counter-clockwise ring current for the embedded azulene subunits and the heptagon (Figure S29a and S29b). NICS(1)$_{zz}$ calculations show that all of the hexagonal rings show aromatic or non-aromatic characters for both compounds **2** and **HBNG1**. The non-benzenoid rings of **HBNG1** have stronger anti-aromatic character than that of compound **2**, which is consistent with the nonplanarity value of these rings (Figure S29c and S29d). This character might be induced by the twisted geometry and intramolecular interaction of the two layers in **HBNG1**. Moreover, the SEC measurements revealed the unique interlayer through-space conjugation in **HBNG1** (Figure 4c), while it's absent in compound **2**.

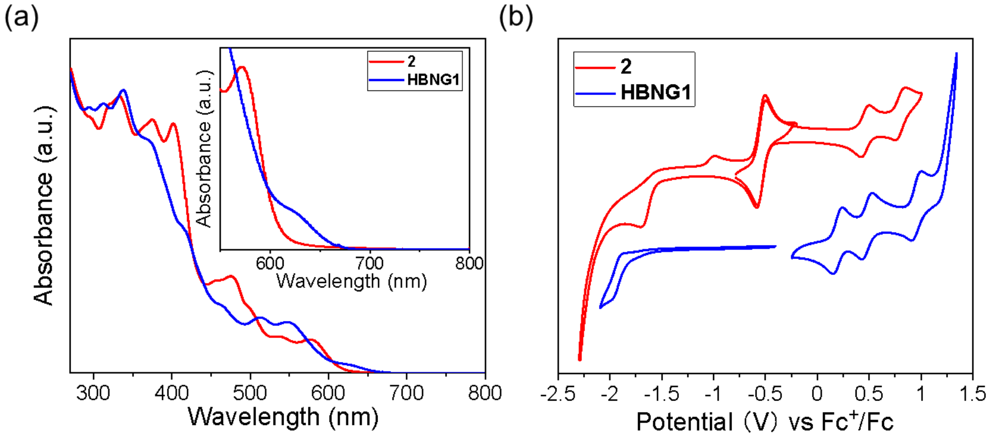


**Figure S28**. (a) UV-Vis absorption spectra of **2** and **HBNG1** in DCM (1×10$^{-5}$ M). (b) CV of **2** and **HBNG1** in DCM containing 0.1 M *n*Bu$_4$NPF$_6$ against Fc$^+$/Fc at a scan rate of 50 mVs$^{-1}$ at room temperature.

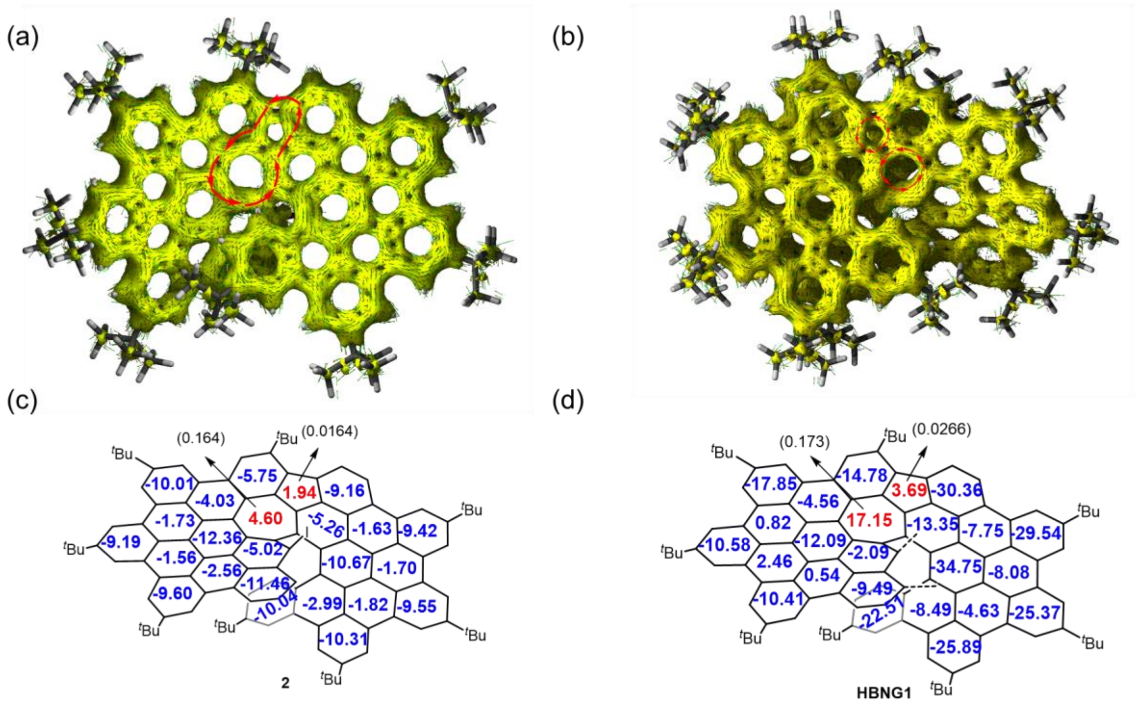


**Figure S29**. Calculated ACID plots for **2** (a).and **HBNG1** (b), NICS(1)$_{zz}$ values of the identical rings of **2** (c) and **HBNG1** (d), calculated at the GIAO-B3LYP/6 31+G(2d,p) level of theory, and the nonplanarity value of the pentagon and heptagons in parentheses.

## 12. NMR spectra

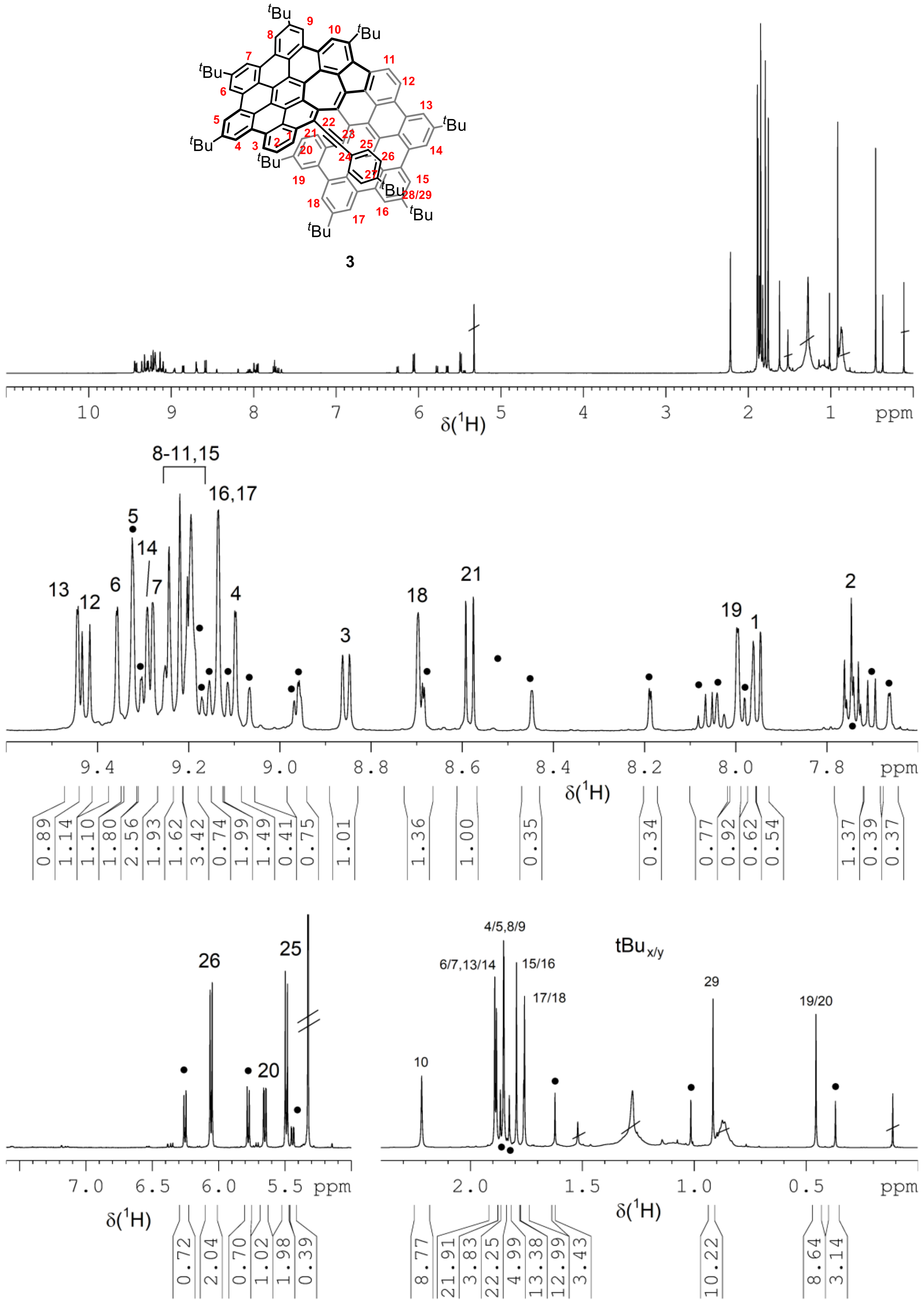

**Figure S30**. $^{1}$H NMR spectrum (overview and regions) of **3** ($CD_2Cl_2$). The signals of the minor isomer are marked with dots. The assignment is reported in the synthesis of **3**.

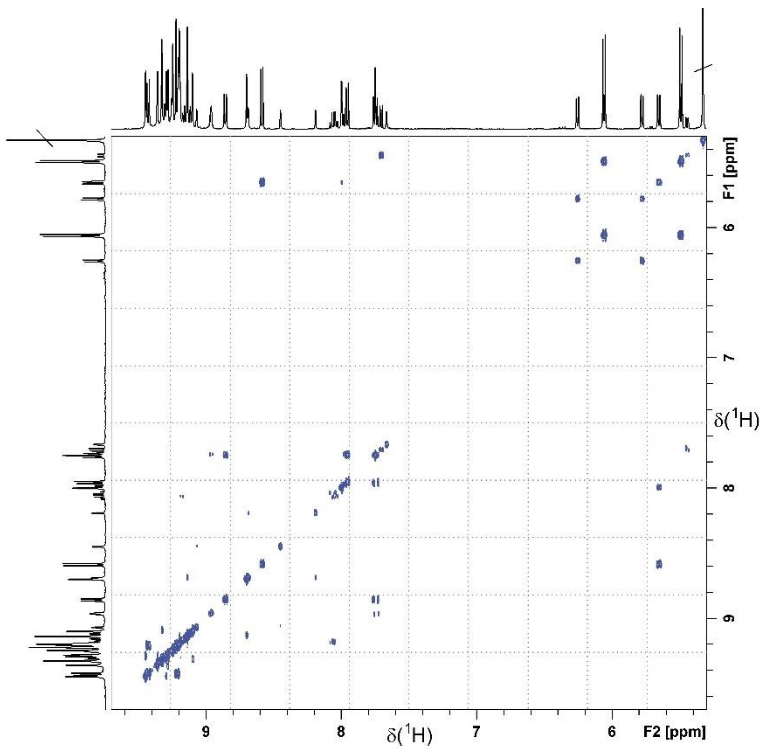


**Figure S31**. COSY spectrum (region) of **3** ($CD_2Cl_2$).

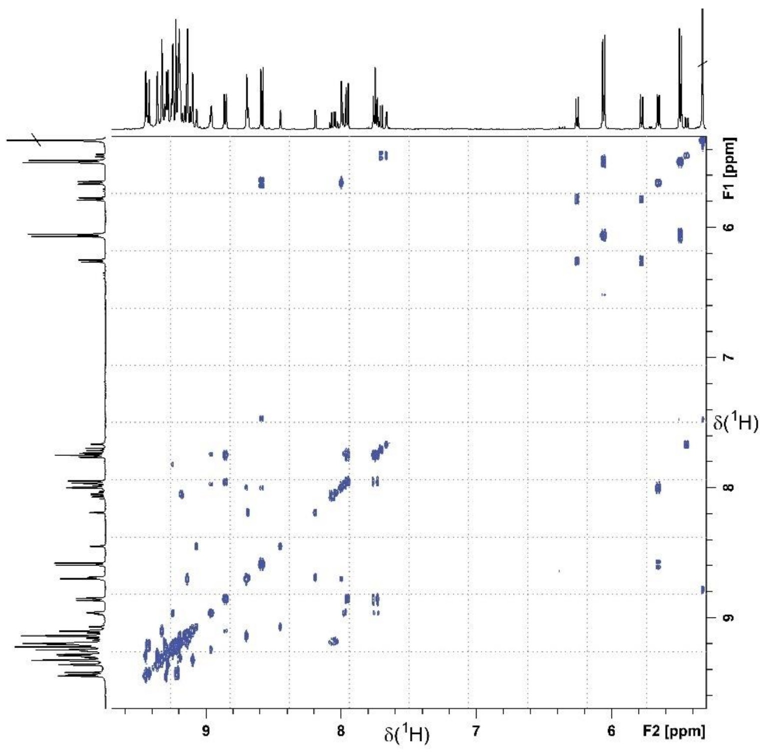


**Figure S32**. Long-range COSY spectrum (region) of **3** ($CD_2Cl_2$).

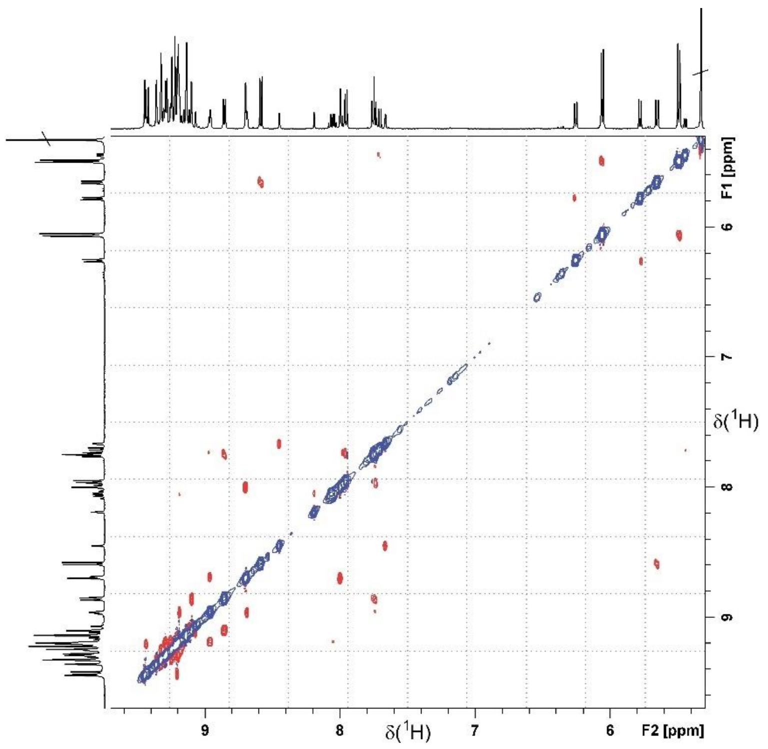


**Figure S33**. ROESY spectrum (region) of **3** ($CD_2Cl_2$).

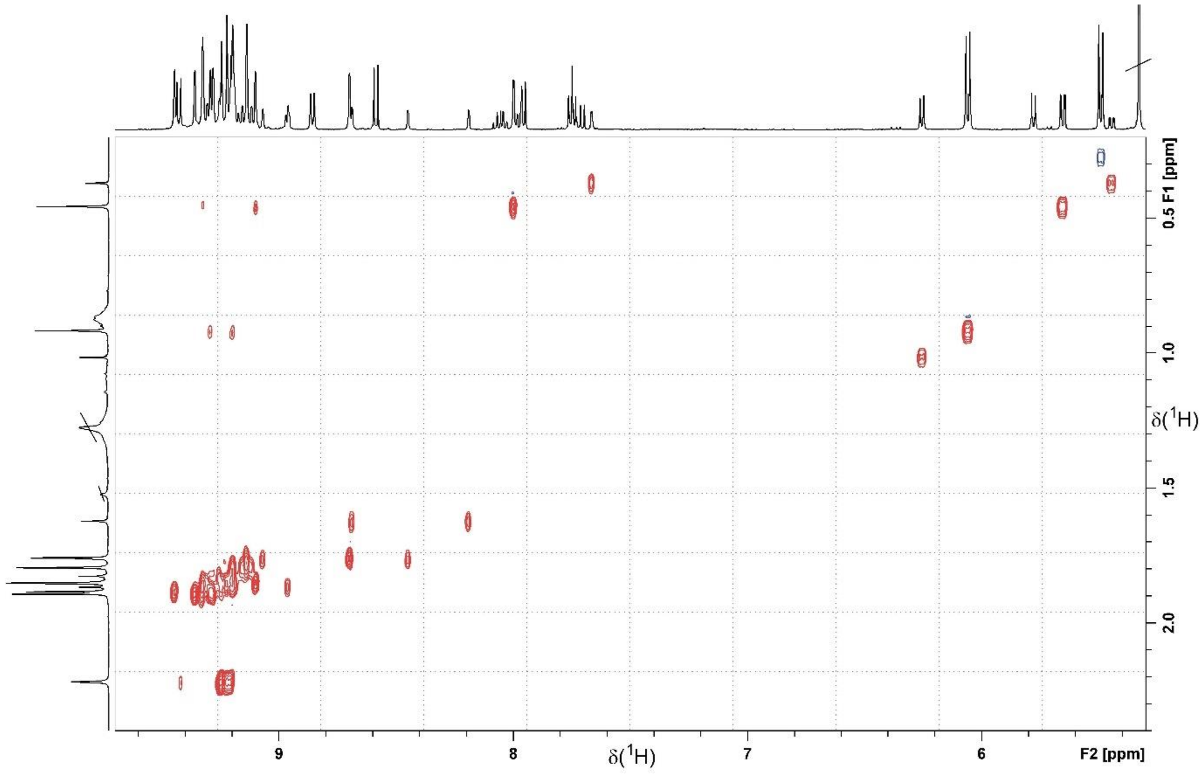


**Figure S34**. ROESY spectrum (correlations of *t*Bu groups) of **3** ($CD_2Cl_2$).

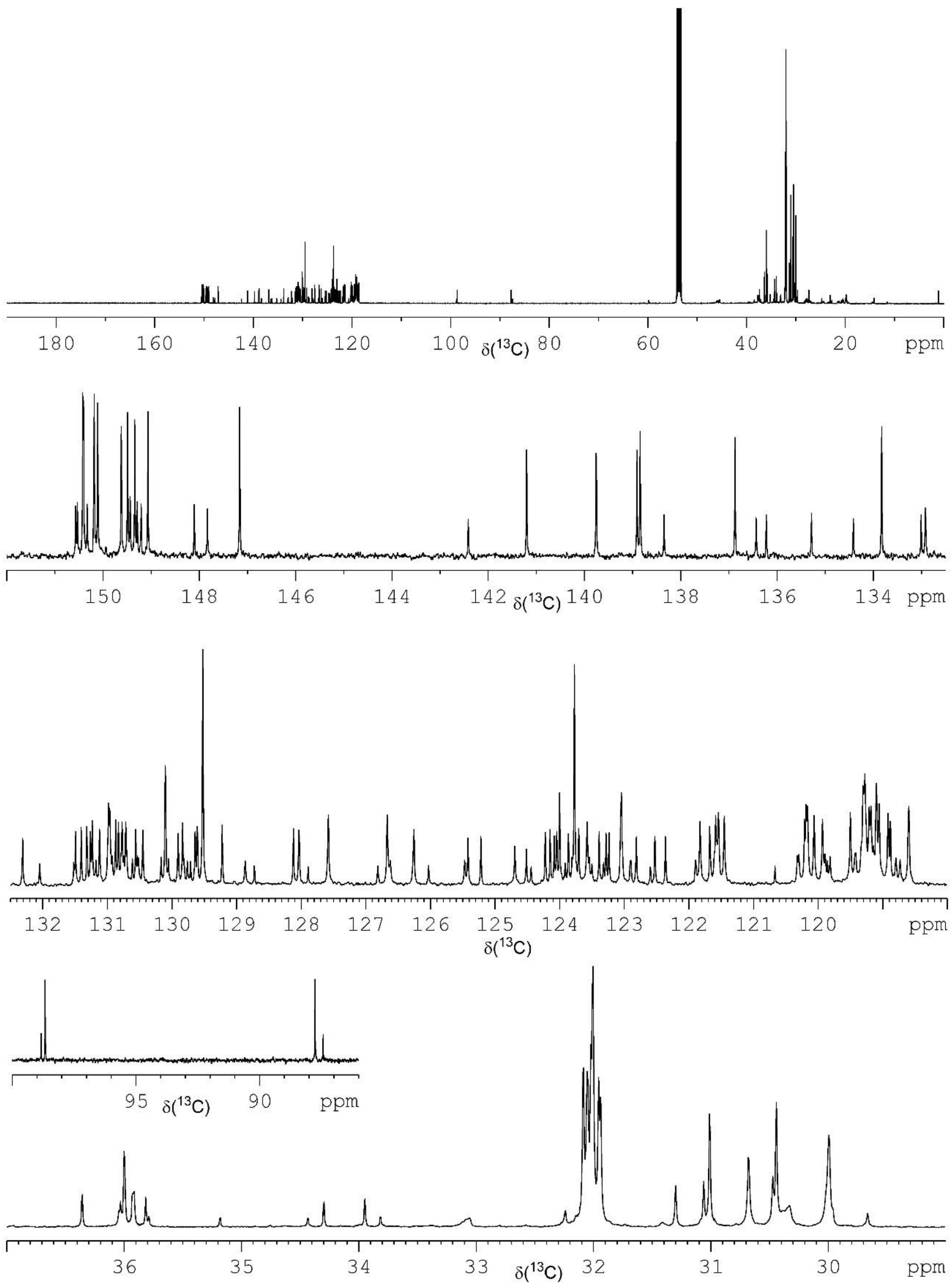


**Figure S35**. $^{13}C$ NMR spectrum (overview and regions) of **3** ($CD_2Cl_2$).

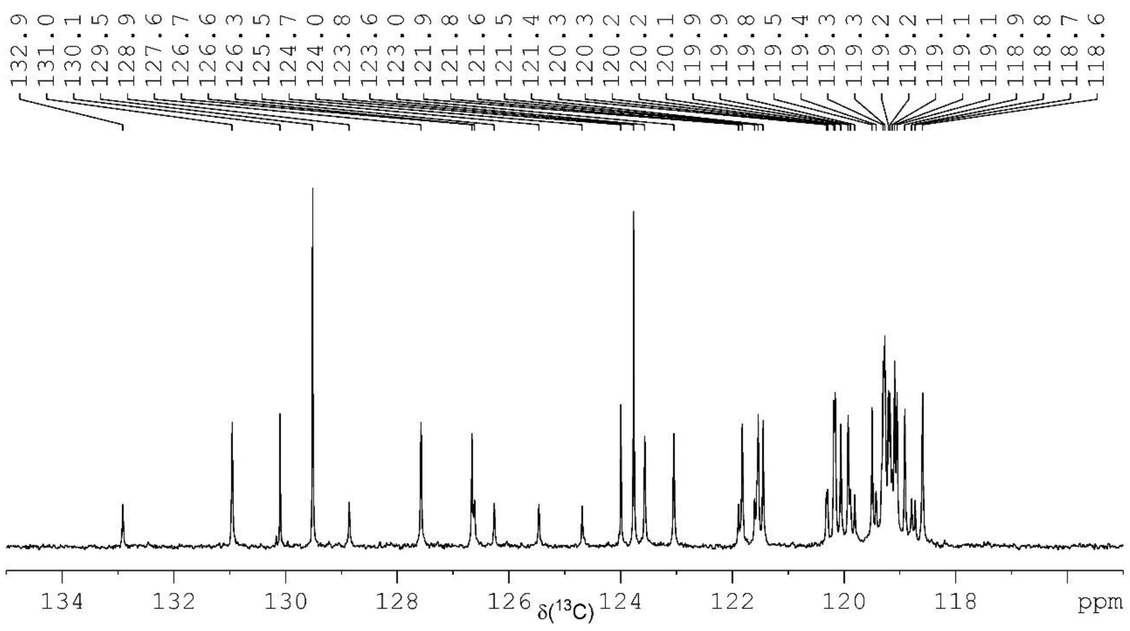


**Figure S36**. DEPT135 spectrum (region of aromatic CH groups) of **3** ($CD_2Cl_2$).

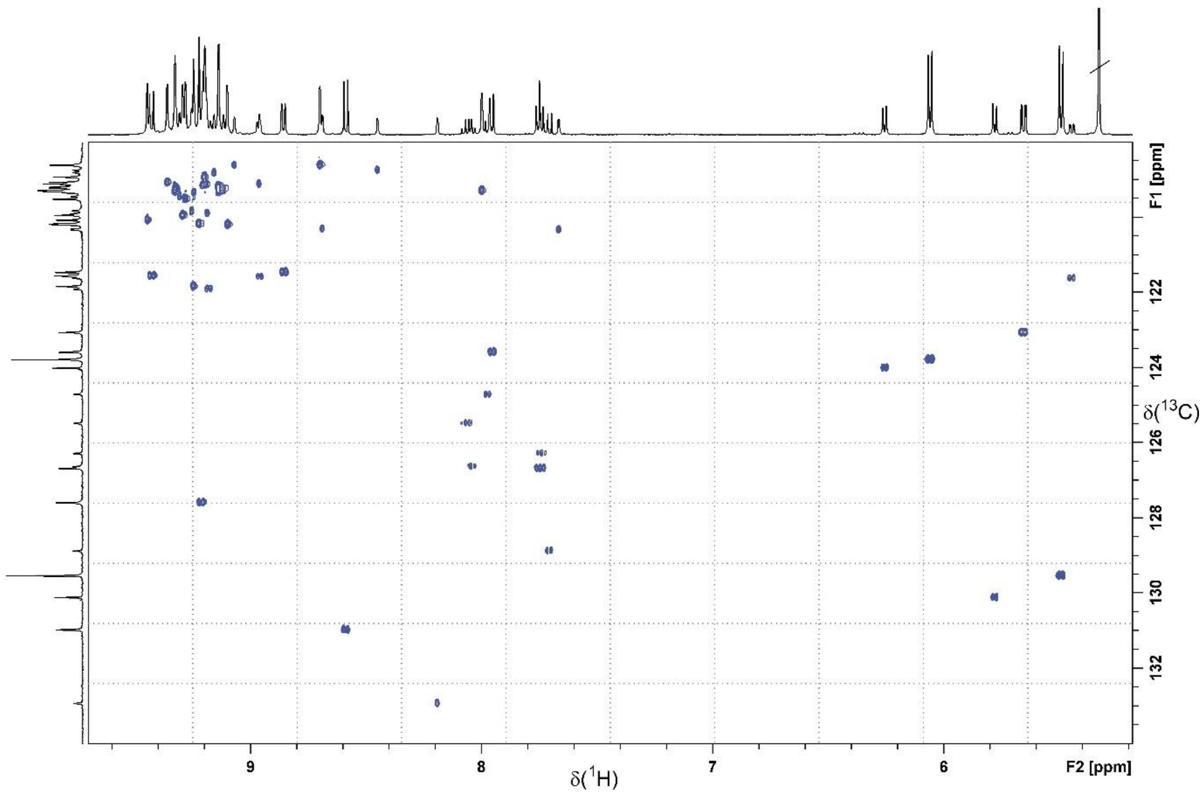


**Figure S37**. HSQC spectrum (correlations of aromatic CH groups) of **3** ($CD_2Cl_2$).

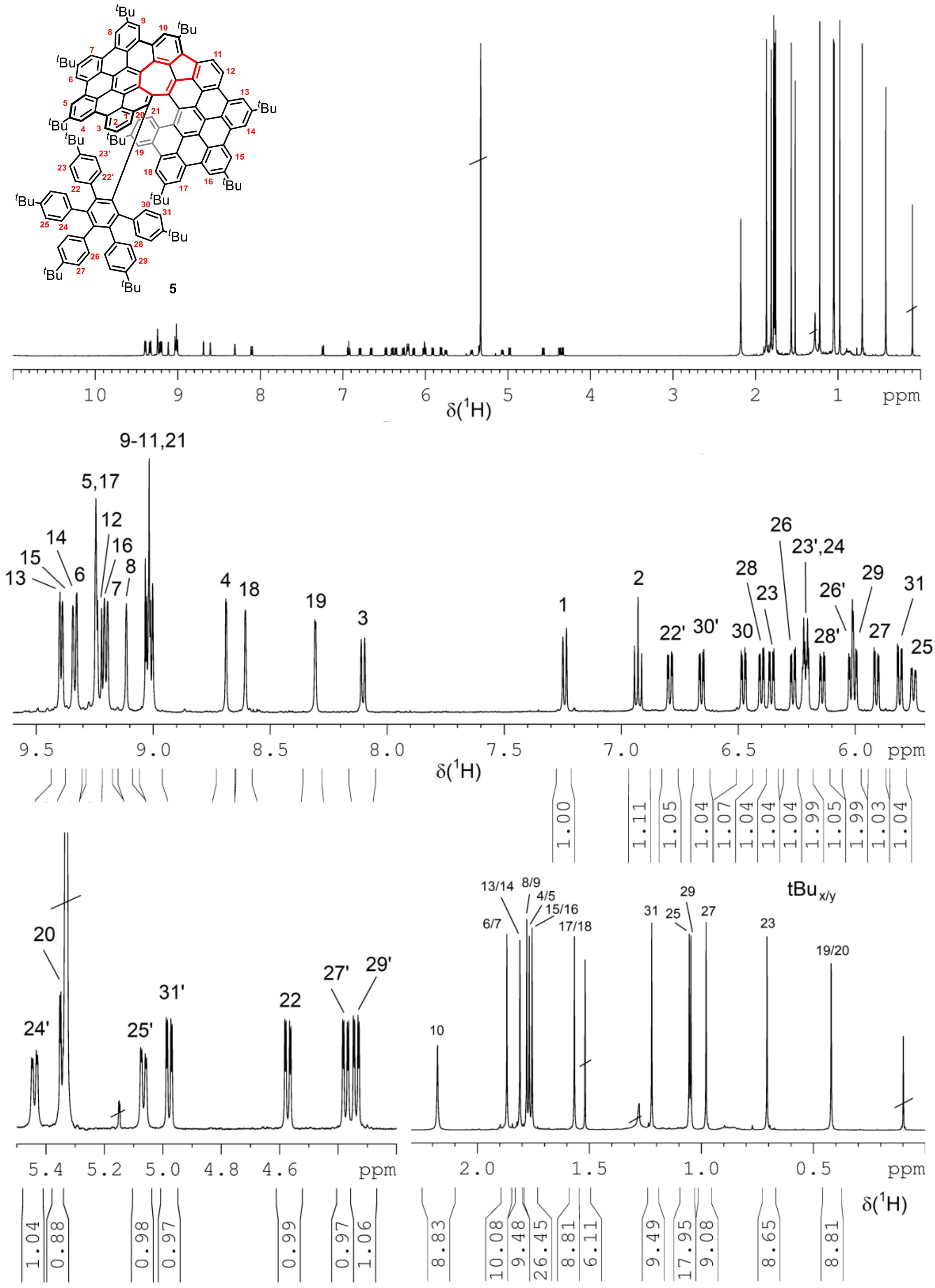

**Figure S38**. $^1$H NMR spectrum (overview and regions) of **5** ($CD_2Cl_2$).

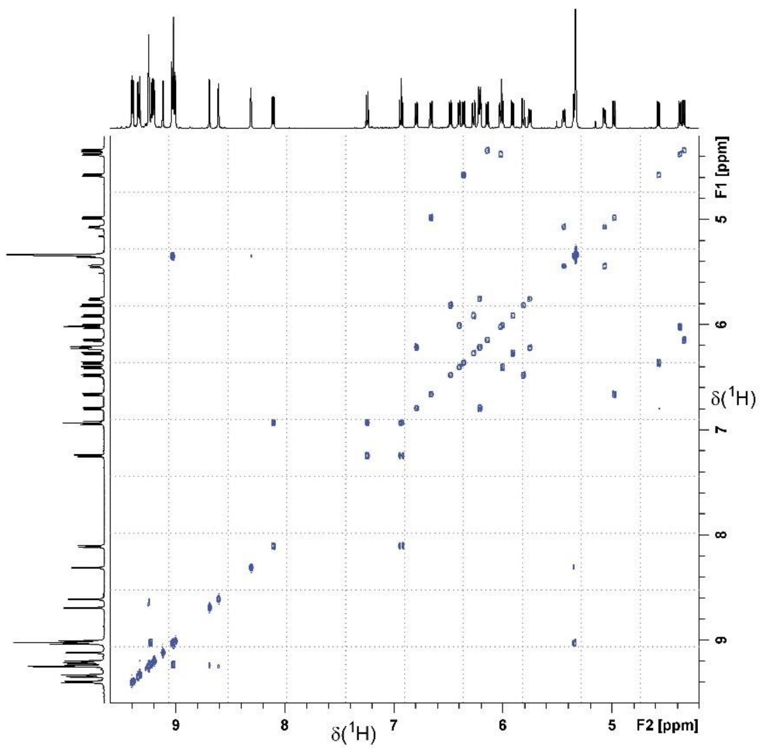


**Figure S39**. COSY spectrum (region) of **5** ($CD_2Cl_2$).

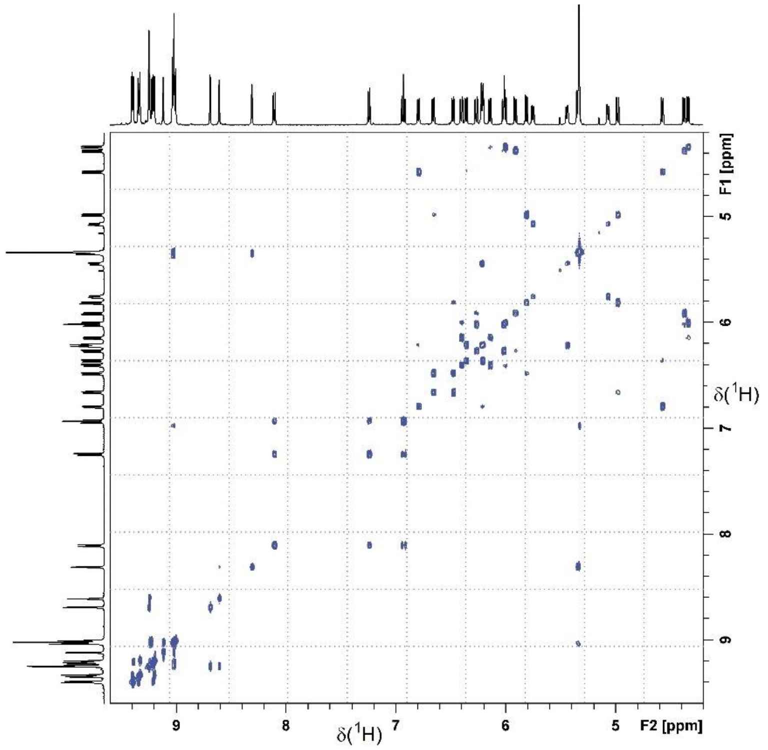


**Figure S40**. Long-range COSY spectrum (region) of **5** ($CD_2Cl_2$).

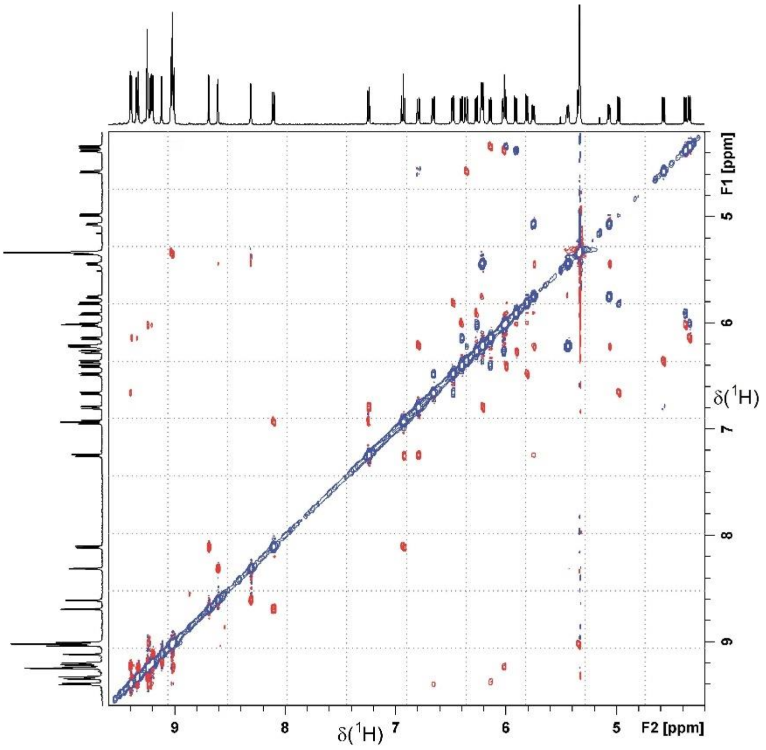


**Figure S41**. ROESY/EXSY spectrum (region) of **5** ($CD_2Cl_2$). The red correlation peaks result from spatial proximity and the blue correlation peaks from exchange processes (phenyl ring rotation).

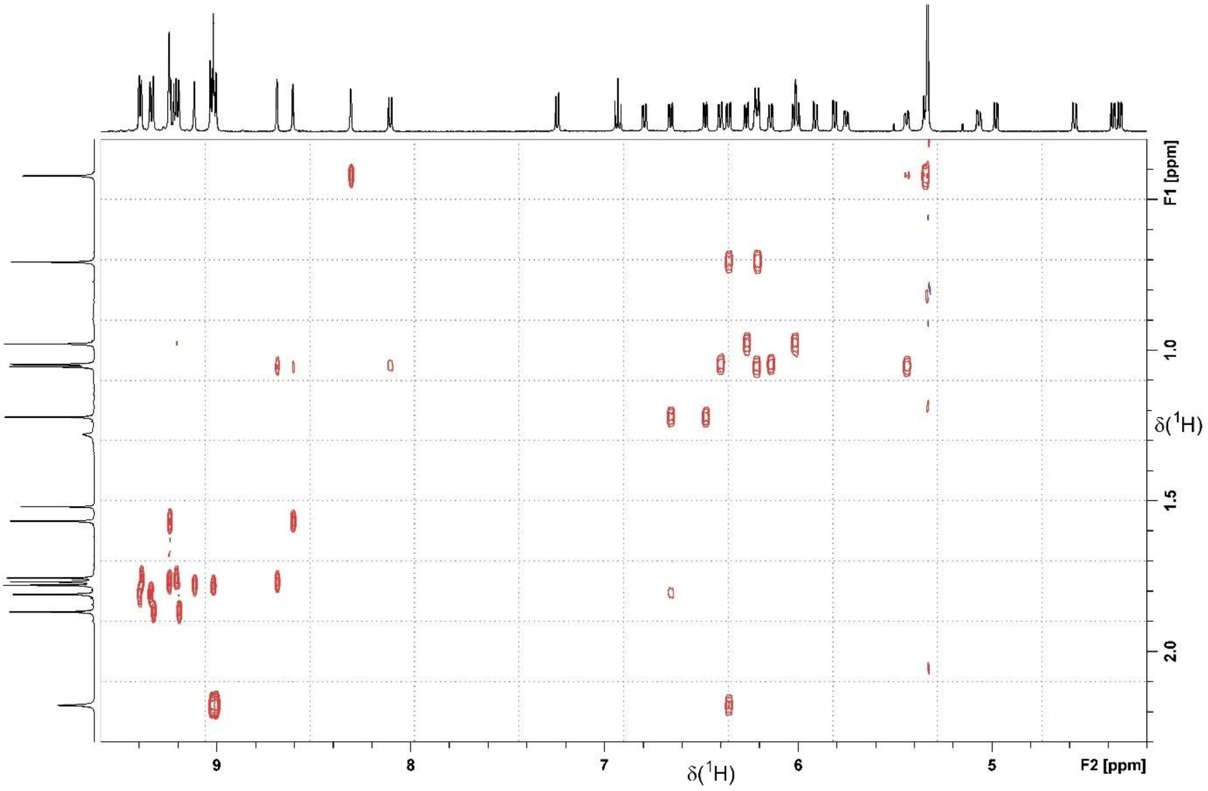


**Figure S42**. ROESY spectrum (correlations of *t*Bu groups) of **5** ($CD_2Cl_2$).

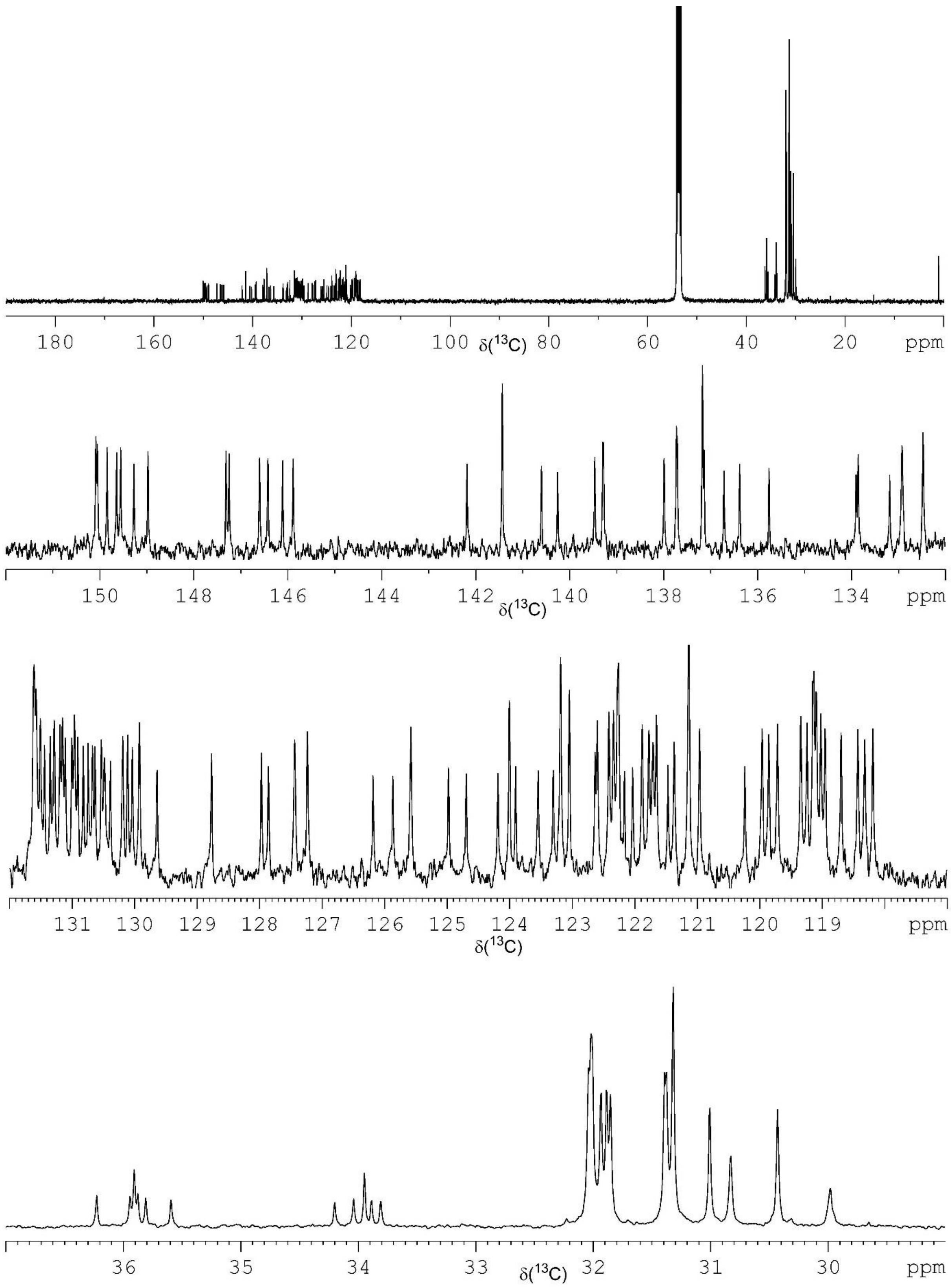


**Figure S43**. $^{13}$C NMR spectrum (overview and regions) of **5** ($CD_2Cl_2$).

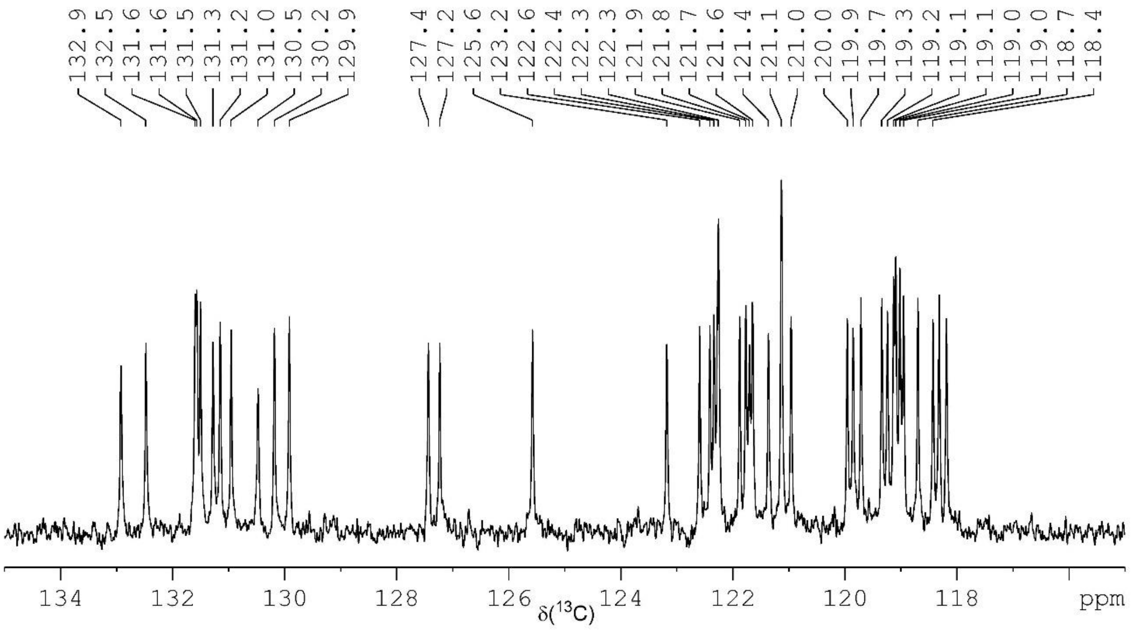


**Figure S44**. DEPT135 spectrum (region of aromatic CH groups) of **5** ($CD_2Cl_2$).

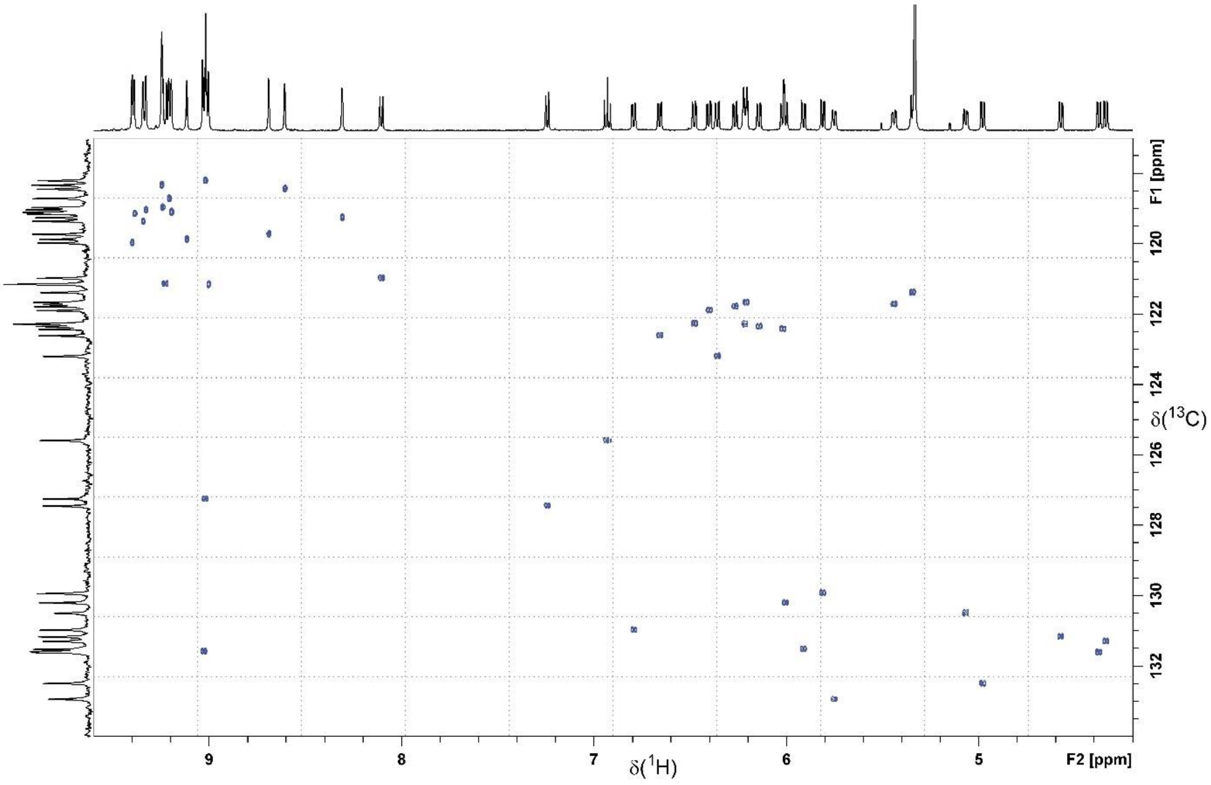


**Figure S45**. HSQC spectrum (correlations of aromatic CH groups) of **5** ($CD_2Cl_2$).

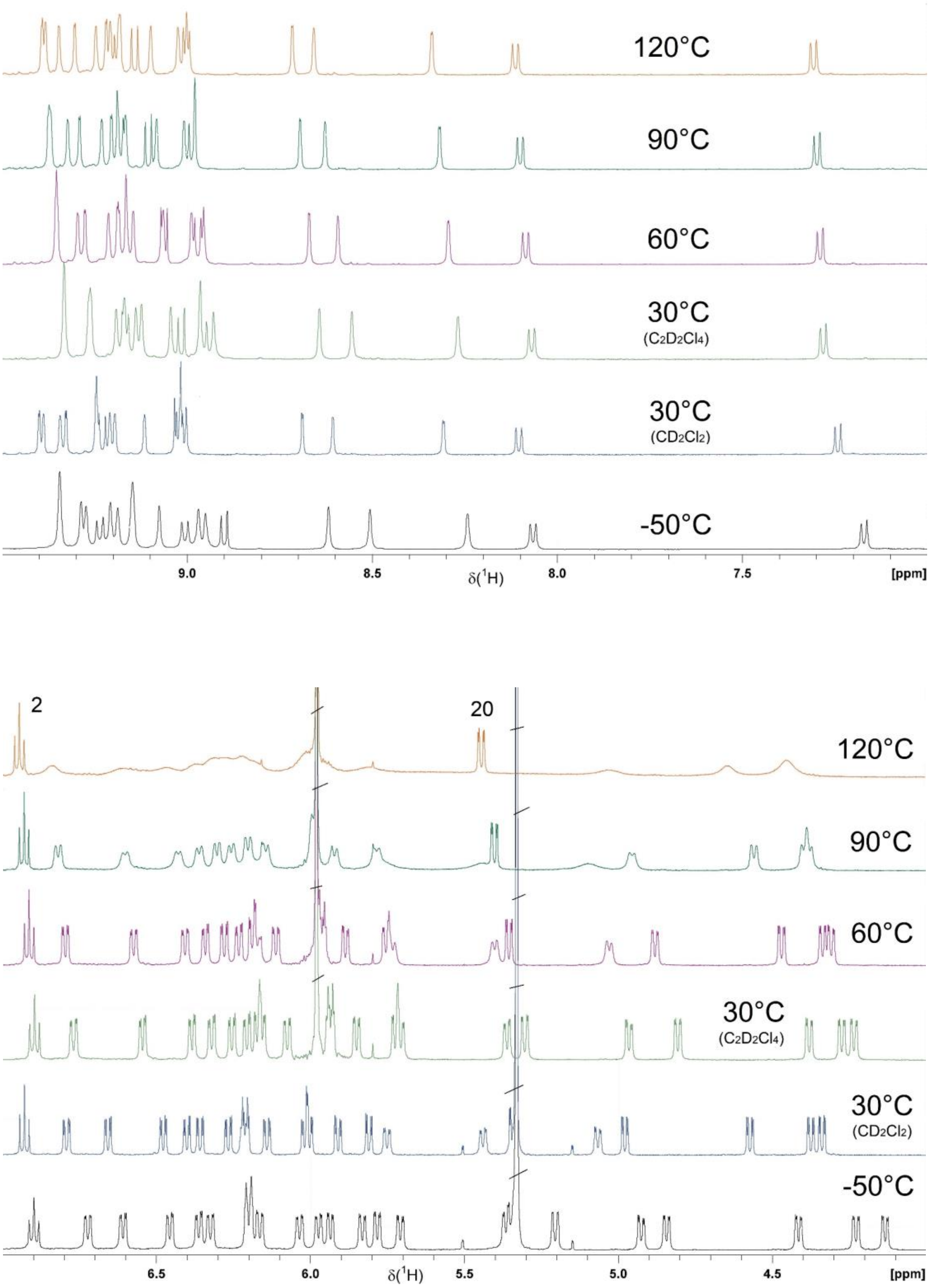


**Figure S46**. $^{1}H$ NMR spectra (region of aromatic protons) of **5** recorded at different temperatures in $CD_2Cl_2$ (30 °C, -50 °C) and in $C_2D_2Cl_4$ (30 °C, 60 °C, 90 °C, 120 °C). *Note:* In addition to minor signal shifts due to temperature and solvent effects, the increased rotational rate of the *para*-substituted phenyl groups results in signal broadening and signal coalescence for the corresponding signals in the 7–4 ppm region.

## 13. High-resolution mass spectrometry (HRMS)

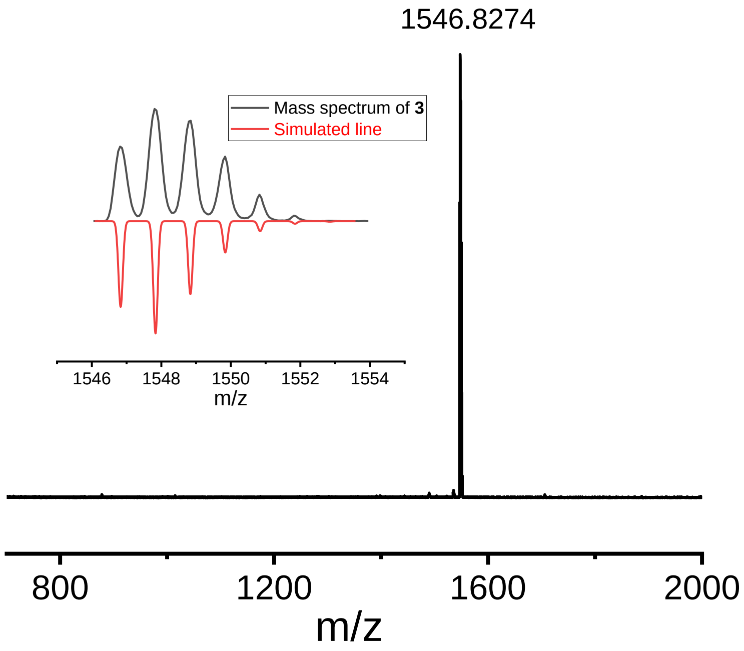


**Figure S47**. HR-MALDI-TOF mass spectrum of compound **3**.

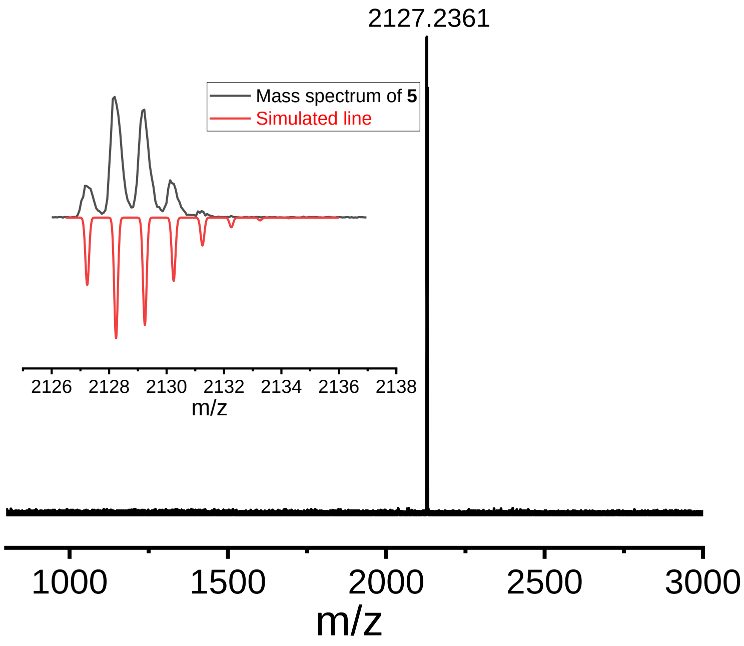


**Figure S48**. HR-MALDI-TOF mass spectrum of compound **5**.

## 14. References

1. J. Ma, Y. Fu, E. Dmitrieva, F. Liu, H. Komber, F. Hennersdorf, A. A. Popov, J. J. Weigand, J. Liu, X. Feng, *Angew. Chem. Int. Ed.* **2020**, *59*, 5637-5642; *Angew. Chem.* **2020**, *132*, 5686-5691

2. O.V. Dolomanov, L.J. Bourhis, R.J Gildea, J.A.K. Howard, H. Puschmann, *J. Appl. Cryst.* **2009**, *42*, 339-341.

3. G.M. Sheldrick, *Acta Cryst.* **2015**, *A71*, 3-8.

4. Gaussian 16, Revision D.01, M. J. Frisch, G. W. Trucks, H. B. Schlegel, G. E. Scuseria, M. A. Robb, J. R. Cheeseman, G. Scalmani, V. Barone, B. Mennucci, G. A. Petersson, H. Nakatsuji, M. Caricato, X. Li, H. P. Hratchian, A. F. Izmaylov, J. Bloino, G. Zheng, J. L. Sonnenberg, M. Hada, M. Ehara, K. Toyota, R. Fukuda, J. Hasegawa, M. Ishida, T. Nakajima, Y. Honda, O. Kitao, H. Nakai, T. Vreven, J. A. Montgomery, Jr., J. E. Peralta, F. Ogliaro, M. Bearpark, J. J. Heyd, E. Brothers, K. N. Kudin, V. N. Staroverov, R. Kobayashi, J. Normand, K. Raghavachari, A. Rendell, J. C. Burant, S. S. Iyengar, J. Tomasi, M. Cossi, N. Rega, J. M. Millam, M. Klene, J. E. Knox, J. B. Cross, V. Bakken, C. Adamo, J. Jaramillo, R. Gomperts, R. E. Stratmann, O. Yazyev, A. J. Austin, R. Cammi, C. Pomelli, J. W. Ochterski, R. L. Martin, K. Morokuma, V. G. Zakrzewski, G. A. Voth, P. Salvador, J. J. Dannenberg, S. Dapprich, A. D. Daniels, Ö. Farkas, J. B. Foresman, J. V. Ortiz, J. Cioslowski, and D. J. Fox, Gaussian, Inc., Wallingford CT, **2016.**

5. D. Geuenich, K. Hess, F. Köhler, R. Herges, *Chem. Rev.* **2005**, *105*, 3758.

6. a) Z. Chen, C. S. Wannere, C. Corminboeuf, R. Puchta, P. V. R. Schleyer, *Chem. Rev.* **2005,** *105*, 3842-3888. b) P. V. R. Schleyer, C. Maerker, A. Dransfeld, H. Jiao, N. J. R. van Eikema Hommes,. *J. Am. Chem. Soc.* **1996,** *118*, 6317-6318.

7. a) E. R. Johnson, S. Keinan, P. Mori-Sánchez, J. Contreras-García, A. J. Cohen, W. Yang,. *J. Am. Chem. Soc.* **2010,** *132*, 6498-6506. b) T. Lu, F. Chen, *J. Comput. Chem.* **2012,** *33*, 580-592.

8. T. Biet, A. Fihey, T. Cauchy, N. Vanthuyne, C. Roussel, J. Crassous, N. Avarvari, *Chem. Eur. J.* **2013,** *19*, 13160-13167.
