# Supplementary material for: Helical Bilayer Nonbenzenoid Nanographene Bearing a [10]Helicene with Two Embedded Heptagons
Source: Angew Chem Int Ed Engl. 2022 Dec 20;62(4):e202216193. doi: 10.1002/anie.202216193 (PMC10107200; doi:10.1002/anie.202216193)

checkCIF/PLATON report

Structure factors have been supplied for datablock(s) exp_3079

THIS REPORT IS FOR GUIDANCE ONLY. IF USED AS PART OF A REVIEW PROCEDURE FOR PUBLICATION, IT SHOULD NOT REPLACE THE EXPERTISE OF AN EXPERIENCED CRYSTALLOGRAPHIC REFEREE.

No syntax errors found. CIF dictionary Interpreting this report

Datablock: exp_3079

Bond precision:	C-C = 0.0044 A		Wavelength=1.54184
Cell:	a=51.3988(5)	b=51.3988(5)	c=23.5569(7)
	alpha=90	beta=90	gamma=90
Temperature:	100 K		

	Calculated	Reported
Volume	62234(2)	62233(2)
Space group	I 41/a	I 41/a
Hall group	-I 4ad	-I 4ad
Moiety formula	C164 H148, C6 H5 Cl [+ solvent]	C164 H148, C6 H5 Cl
Sum formula	C170 H153 Cl [+ solvent]	C170 H153 Cl
Mr	2231.38	2231.36
Dx,g cm-3	0.953	0.953
Z	16	16
Mu (mm-1)	0.556	0.556
F000	19040.0	19040.0
F000'	19092.80	
h,k,lmax	60,60,27	60,60,27
Nref	26459	25640
Tmin,Tmax	0.846,0.946	0.825,1.000
Tmin'	0.846	

Correction method= # Reported T Limits: Tmin=0.825 Tmax=1.000
AbsCorr = MULTI-SCAN

Data completeness= 0.969 Theta(max)= 64.990

R(reflections)= 0.0747(19138) wR2(reflections)= 0.2314(25640)

S = 1.068 Npar= 1558

The following ALERTS were generated. Each ALERT has the format
test-name_ALERT_alert-type_alert-level.
Click on the hyperlinks for more details of the test.

Alert level C

```
THETM01_ALERT_3_C  The value of sine(theta_max)/wavelength is less than 0.590
            Calculated sin(theta_max)/wavelength =    0.5878
PLAT018_ALERT_1_C _diffrn_measured_fraction_theta_max .NE. *_full           ! Check
PLAT220_ALERT_2_C NonSolvent   Resd 1  C   Ueq(max)/Ueq(min) Range        3.5 Ratio
PLAT222_ALERT_3_C NonSolvent Resd 1  H   Uiso(max)/Uiso(min) Range        4.1 Ratio
PLAT242_ALERT_2_C Low    'MainMol' Ueq as Compared to Neighbors of       C126 Check
PLAT250_ALERT_2_C Large U3/U1 Ratio for Average U(i,j) Tensor ....        2.2 Note
PLAT250_ALERT_2_C Large U3/U1 Ratio for Average U(i,j) Tensor ....        3.3 Note
PLAT260_ALERT_2_C Large Average Ueq of Residue Including       Cl2      0.121 Check
PLAT340_ALERT_3_C Low Bond Precision on  C-C Bonds ...............    0.00444 Ang.
PLAT410_ALERT_2_C Short Intra H...H Contact  H100     ..H111     .       1.97 Ang.
                                                 x,y,z  =      1_555 Check
PLAT410_ALERT_2_C Short Intra H...H Contact  H117     ..H130     .       1.97 Ang.
                                                 x,y,z  =      1_555 Check
PLAT906_ALERT_3_C Large K Value in the Analysis of Variance ......      2.599 Check
PLAT910_ALERT_3_C Missing # of FCF Reflection(s) Below Theta(Min).          5 Note
PLAT911_ALERT_3_C Missing FCF Refl Between Thmin & STh/L=    0.588        814 Report
PLAT918_ALERT_3_C Reflection(s) with I(obs) much Smaller I(calc) .          1 Check
PLAT977_ALERT_2_C Check Negative Difference Density on H05D      .      -0.34 eA-3
```

Alert level G

```
PLAT012_ALERT_1_G No       _shelx_res_checksum Found in CIF ......     Please Check
PLAT014_ALERT_1_G No       _shelx_fab_checksum Found in CIF ......     Please Check
PLAT072_ALERT_2_G SHELXL First  Parameter in WGHT  Unusually Large       0.13 Report
PLAT083_ALERT_2_G SHELXL Second Parameter in WGHT  Unusually Large      74.45 Why ?
PLAT171_ALERT_4_G The CIF-Embedded .res File Contains EADP Records          1 Report
PLAT231_ALERT_4_G Hirshfeld Test (Solvent)   Cl2      --C05K     .       42.7 s.u.
PLAT231_ALERT_4_G Hirshfeld Test (Solvent)   Cl3      --C05D     .       26.3 s.u.
PLAT300_ALERT_4_G Atom Site Occupancy of Cl2        Constrained at        0.5 Check
PLAT300_ALERT_4_G Atom Site Occupancy of Cl3        Constrained at        0.5 Check
PLAT300_ALERT_4_G Atom Site Occupancy of H05D       Constrained at        0.5 Check
PLAT300_ALERT_4_G Atom Site Occupancy of H05K       Constrained at        0.5 Check
PLAT302_ALERT_4_G Anion/Solvent/Minor-Residue Disorder (Resd  2  )        14% Note
PLAT333_ALERT_2_G Large Aver C6-Ring C-C Dist C1       -C41      .       1.44 Ang.
PLAT333_ALERT_2_G Large Aver C6-Ring C-C Dist C30      -C41      .       1.43 Ang.
PLAT606_ALERT_4_G Solvent Accessible VOID(S) in Structure ........          ! Info
PLAT720_ALERT_4_G Number of Unusual/Non-Standard Labels ..........         16 Note
PLAT909_ALERT_3_G Percentage of I>2sig(I) Data at Theta(Max) Still        60% Note
PLAT913_ALERT_3_G Missing # of Very Strong Reflections in FCF ....          1 Note
PLAT933_ALERT_2_G Number of HKL-OMIT Records in Embedded .res File        150 Note
PLAT941_ALERT_3_G Average HKL Measurement Multiplicity ...........        3.3 Low
PLAT967_ALERT_5_G Note: Two-Theta Cutoff Value in Embedded .res ..      130.0 Degree
PLAT978_ALERT_2_G Number C-C Bonds with Positive Residual Density.          2 Info
PLAT992_ALERT_5_G Repd & Actual _reflns_number_gt Values Differ by          2 Check
```

0 **ALERT level A** = Most likely a serious problem - resolve or explain
0 **ALERT level B** = A potentially serious problem, consider carefully
16 **ALERT level C** = Check. Ensure it is not caused by an omission or oversight

```
23 ALERT level G = General information/check it is not something unexpected

 3 ALERT type 1 CIF construction/syntax error, inconsistent or missing data
14 ALERT type 2 Indicator that the structure model may be wrong or deficient
10 ALERT type 3 Indicator that the structure quality may be low
10 ALERT type 4 Improvement, methodology, query or suggestion
 2 ALERT type 5 Informative message, check
```

It is advisable to attempt to resolve as many as possible of the alerts in all categories. Often the minor alerts point to easily fixed oversights, errors and omissions in your CIF or refinement strategy, so attention to these fine details can be worthwhile. In order to resolve some of the more serious problems it may be necessary to carry out additional measurements or structure refinements. However, the purpose of your study may justify the reported deviations and the more serious of these should normally be commented upon in the discussion or experimental section of a paper or in the "special_details" fields of the CIF. checkCIF was carefully designed to identify outliers and unusual parameters, but every test has its limitations and alerts that are not important in a particular case may appear. Conversely, the absence of alerts does not guarantee there are no aspects of the results needing attention. It is up to the individual to critically assess their own results and, if necessary, seek expert advice.

Publication of your CIF in IUCr journals

A basic structural check has been run on your CIF. These basic checks will be run on all CIFs submitted for publication in IUCr journals (*Acta Crystallographica*, *Journal of Applied Crystallography*, *Journal of Synchrotron Radiation*); however, if you intend to submit to *Acta Crystallographica Section C* or *E* or *IUCrData*, you should make sure that full publication checks are run on the final version of your CIF prior to submission.

Publication of your CIF in other journals

Please refer to the *Notes for Authors* of the relevant journal for any special instructions relating to CIF submission.

PLATON version of 18/05/2022; check.def file version of 17/05/2022

Datablock exp_3079 - ellipsoid plot

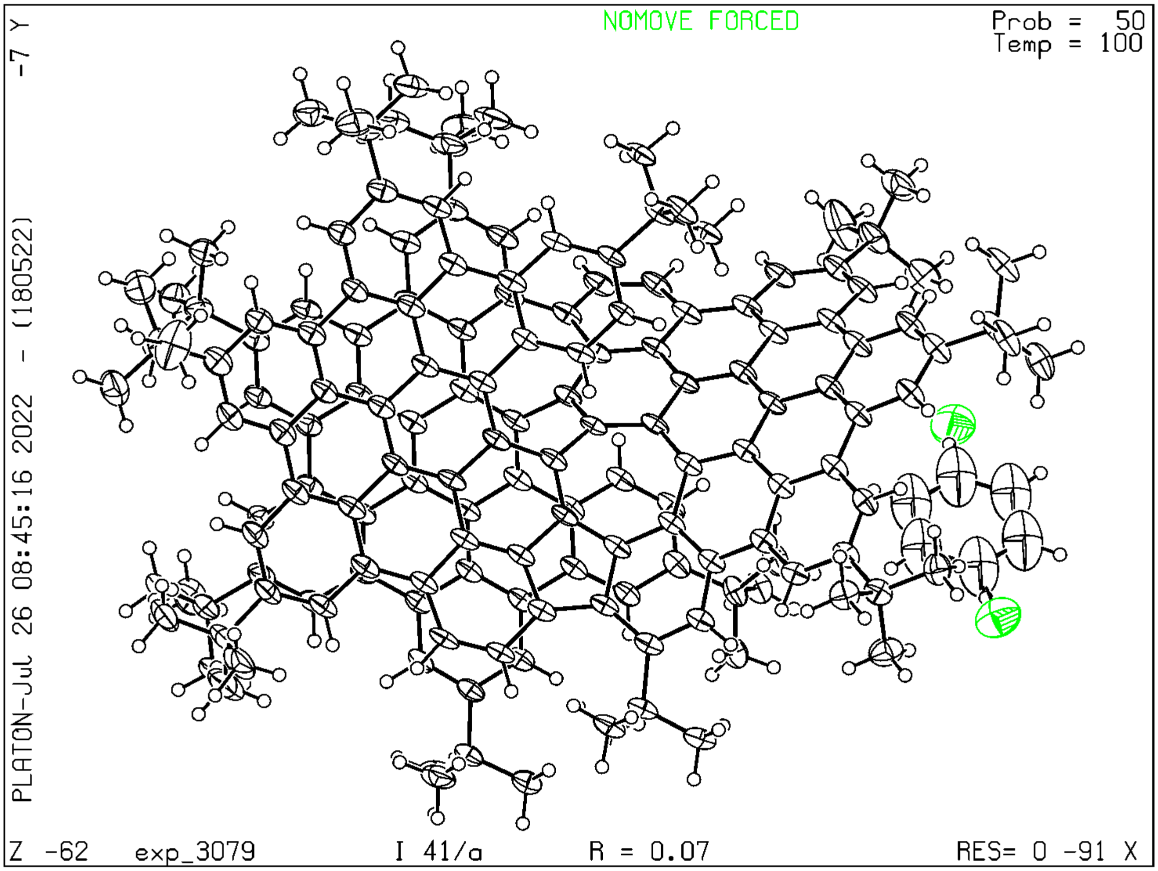

Supplement: Supplementary file 2 — Supporting Information [file ANIE-62-0-s002.pdf]
